# Development and optimization of Moxifloxacin solid lipid nanoparticles via double emulsion organic solvent free technique applying Box–Behnken experimental design

**DOI:** 10.1038/s41598-025-26860-x

**Published:** 2025-11-26

**Authors:** Esraa M. Elshazly, Mona G. Arafa, Samia A. Nour

**Affiliations:** 1 Department of Pharmaceutics and Pharmaceutical Technology, Faculty of Pharmacy, The British University in Egypt, Cairo, Egypt; 2 Department of Pharmaceutics and Industrial Pharmacy, Faculty of Pharmacy, Cairo University, Cairo, Egypt; 3 Chemotherapeutic Unit, Mansoura University Hospitals, Mansoura, Egypt; 4 Nanotechnology Research Center, The British University in Egypt, Cairo, Egypt

**Keywords:** Chronic wounds, Moxifloxacin, Nanotechnology, Solid lipid nanoparticles, Double emulsion technique, Box–Behnken design, Optimization, Biotechnology, Chemistry, Drug discovery, Materials science, Nanoscience and technology

## Abstract

The current research proposes the preparation of Moxifloxacin-loaded solid lipid nanoparticles through a solvent-free double emulsion technique, to overcome chronic wound healing limitation. The independent variables: stearic acid, Span 80, and Tween 80 were optimized via Box–Behnken design, based on entrapment efficiency (EE%), zeta potential (ZP), and particle size (PS). To statistically optimize the formulation variables, a three-factor, three-level (3³) design involving 15 runs was employed and optimization was carried out to attain maximum EE% and ZP and minimum PS yielding an overall desirability of 0.851. Results indicated that increasing stearic acid enhanced EE%, Span 80 increase PS, and Tween 80 improved ZP. The Optimized SLNs formula had a ZP of − 52.4 mV, EE% of ~ 79.6% and PS of 257 nm. Chemical compatibility was emphasized using FTIR, and XRD studies revealed reduced drug crystallinity within the lipid matrix, which confirmed successful encapsulation. Transmission electron microscopy (TEM) and scanning electron microscopy (SEM) imaging revealed nanoparticles with defined spherical shape and smooth surface. The optimized formula is anticipated to enhance dermal distribution and prolonged antibiotic release, thereby offering an efficient therapeutic strategy for the management of chronic wounds.

**Supplementary Information:**

The online version contains supplementary material available at 10.1038/s41598-025-26860-x.

## Introduction

 The process of wound healing in healthy individuals is highly precise and integrated. Any interruption in this precise and methodical process can result in the formation of chronic wounds^1^. Diabetes mellitus (DM) is a principal factor that hinders wound healing, recognized as the most common chronic disease^2,3^. Approximately 15% of diabetic patients develop foot ulcers, which are particularly prone to infections. Infected wounds considerably hinder normal healing. The International Working Group on the Diabetic Foot (IWGDF) 2019 guidelines^4^ recommended the empirical administration of broad-spectrum antibiotics such as penicillins, fluoroquinolones, and cephalosporins, either orally or intravenously, to prevent additional problems. Moxifloxacin (MOX), a broad-spectrum hydrophilic fluoroquinolone antibiotic^5^, is recognized for its strong antibacterial efficacy and effectiveness in treating infections. Nonetheless, the transdermal administration of hydrophilic pharmaceuticals presents considerable obstacles owing to their limited permeability across the lipid-dense stratum corneum. Nanotechnology has emerged as a promising approach to address drawbacks of traditional dosage forms by enhancing medicinal efficacy^6,7^. Nanoparticles provide multiple methods to improve wound healing, as various studies have shown their capacity to accelerate the healing^8–10^. Expanding upon these recent advances, current nanomedicines have explored multifunctional nanogels and hydrogel-based systems for stimuli-controlled delivery of drugs or biomolecules for infected wound healing. For instance, carboxymethyl chitosan–lysozyme nanogels with antibacterial loading have activated remineralization and inhibited microbes in primary enamel lesions^11^, and DNA hydrogels supporting light-activated gas therapy and on-demand release of exosomes have shown substantial antibacterial and tissue-regenerative activities^12^. These examples highlight the growing potential of nanostructured systems for prolonged, targeted, biocompatible antibacterial therapy, to justify the idea of developing lipid nanoparticles with MOX for the treatment of diabetic wounds. The incorporation of hydrophilic pharmaceuticals such as MOX into solid lipid nanoparticles (SLNs) has demonstrated an enhancement in skin penetration and an improvement in drug delivery. SLNs enhance drug stability and bioavailability while facilitating regulated and sustained release, which is especially advantageous for wound healing^13^. The therapeutic potential of MOX-loaded SLNs formulation in wound treatment was supported with previous studies exhibiting enhanced antibacterial activity and enhanced wound healing with similar formulation (fluoroquinolone-loaded SLNs). For instance, Ciprofloxacin, one of fluoroquinolone drugs, incorporated into SLNs via a double-emulsion process led to enhanced epithelial thickness and enhanced wound healing in comparison to free drug in both in vitro and in vivo mouse models of wound healing^14^. In another example^15^, Ciprofloxacin encapsulated in cationic SLNs exhibited more than 85% entrapment efficiency. This nanosystem presented significantly enhanced antibacterial activity towards Gram-negative and Gram-positive bacteria and exhibited a sustained release. Furthermore, local MOX was found clinically to accelerate wound closure^16^ as in one study^17^ mentioned that the application of topical MOX significantly enhanced the healing of the wound, enhanced granulation tissue formation, and reduced infection compared to placebo, testifying to the efficiency of the fluoroquinolone antibiotic in wound healing when used topically. Despite these advantages, encapsulating hydrophilic medicines into SLNs remains challenging due to the inadequate interaction between hydrophilic and lipophilic components, which often leads to phase separation. This separation compromises drug delivery efficiency and results in poor release profiles. The double emulsion method is the optimal choice for hydrophilic drugs, as it prevents drug dispersion and leakage into the external aqueous layer of the emulsion^18^. Studies have suggested that the double-emulsion technique is an effective approach to address this issue, facilitating better incorporation efficiency and enhanced performance^19^, as the drug is confined within the inner aqueous core. The multilayer structure minimizes drug leakage, enhances stability, and improves both the shelf life and effectiveness of SLNs. Moreover, employing this technique without organic solvents without using any organic solvent further increases the advantages of the method, making it safer and more environmentally friendly^20^. The Box-Behnken design (BBD) is utilized as a statistical tool to evaluate the impact of various independent factors on the targeted responses, hence optimizing the preparation process. Compared to full factorial design, BBD facilitates an effective optimization process with fewer trials. This is especially beneficial for examining the relationships between independent variables and their effects on outcomes such as encapsulation efficiency EE%, PS, and ZP^21^. Although previous researches have explored the integration of double-emulsion techniques with optimization using BBD and comprehensive physicochemical evaluation for localized or controlled drug delivery systems^22–27^, some of these reports used the conventional solvent^23–27^ based process or used other antibiotic model^23,25,27^, in the present study, a green organic-solvent-free double-emulsion–melt-dispersion approach was applied to encapsulate the hydrophilic fluoroquinolone MOX loaded with SLNs, representing a distinctive formulation strategy that enhances drug entrapment efficiency while eliminating residual solvent concerns. Based on this rationale, the present research aimed to develop and evaluate SLNs containing MOX through the double-emulsion method to boost skin absorption, improve drug stability, and promote wound healing. The study utilized thorough analysis through the BBD, examining the impact of several independent parameters: amount of used solid lipid and amounts of utilized surfactants on EE%, PS, and ZP. The study further examined the interaction among individual variables by infrared (IR) spectroscopy and X-ray diffraction (XRD) to verify compatibility and to evaluate crystalline characteristics. SEM and TEM were employed for microscopic examinations to check the morphology of the optimized SLNs.

## Materials

Moxifloxacin was generously provided by Eva Pharmaceutics, Cairo, Egypt. Stearic acid (reagent grade, 95%) and Poloxamer 80 were obtained from Sigma-Aldrich, St. Louis, MO, USA. Tween 80 (Polysorbate 80) was purchased from Fisher Scientific, Loughborough, UK. Span 80 (Sorbitan monooleate) was purchased from Sigma-Aldrich, USA. L-α-Lecithin, granular (from soybean oil) was purchased from ACROS Organics (Geel, Belgium), and lecithin (90%, from soybean, solid) was obtained from Alfa Aesar (Thermo Fisher Scientific, Germany). All chemicals and reagents used were of analytical reagent grade to ensure reliable results.

## Methods

### Ultraviolet (UV) spectrophotometry evaluation of MOX

MOX solution was prepared in 100 ml volumetric flask of 100 µg MOX in 100 ml distilled water. Then the solution was scanned using a spectrophotometer to determine the maximum wavelength of absorbance (λ _max)_ in the ultraviolet range of 200 to 400 nm using the UV/ Visible Spectrophotometer (Jasco – V-630- Japan).

### Preparation of MOX SLNs

#### Selection of independent variables

Critical independent parameters in this method include the type and quantity of lipid (single or multiple), type and concentration of surfactants, the addition of cosurfactants, processing temperature, the ratio of double emulsion to cold water, stirring time and amplitude^28^. Optimizing these parameters helps overcome the major drawbacks of this technique, such as instability due to coalescence of aqueous droplets within the oily phase, aggregation tendencies, and larger particle sizes^29^. Prior to designing complete experimental study, an initial empirical screening was conducted in the laboratory to inform the choice of preparation components, evaluating different reagents for the preparation of SLNs containing a hydrophilic drug (MOX). The SLNs were prepared using the double emulsion method (w/o/w), which requires the use of solid lipid and two surfactants: one in the primary (w/o) emulsion phase and another in the secondary (w/o/w) phase, to ensure emulsion stability. In the primary phase, the surfactant was mixed with the melted lipid and should possess a low hydrophilic-lipophilic balance (HLB) value, indicating lipophilic properties^30^ .For the primary emulsion, two different lecithins with varying molecular weights were evaluated. L-α-Lecithin granular and lecithin 90% solid), Subsequently, Span 80 was evaluated. For the secondary phase, Poloxamer 80 and Tween 80 were tested at low concentrations. The selection of such excipients and screening was based on previous literature findings^22,28,31–33^ and preliminary laboratory experiments^34^. For example, nonionic surfactants Tween 80 and Span 80 have been widely utilized in SLN systems to prevent coalescence and facilitate stable droplet formation^35,36^. Lecithin was utilized as a surfactants and co-surfactant to improve the homogeneity of emulsion and biocompatibility^37^, while Poloxamer 188 was utilized for the improvement of nanoparticle dispersion and aggregation reduction^38,39^.

####  Experimental design and statistical analysis

A three-factor, three-level (3^3^) BBD was applied to statistically optimize the preparation of MOX SLNs. Fifteen trials of SLNs were conducted, involving three center points, using Design-Expert software (version 13.0.5.0). The BBD was used to study the response surface methodology involving three independent variables: the amount of lipid stearic acid, the amount of the first surfactant (Span 80, B), and the percent of the second surfactant solution (Tween 80, C). The evaluation of each variable was carried out at three levels (− 1,0,1) the actual values of these levels were shown in Table 1. These ranges were chosen according to pervious literatures with some modification^22,40,41^ as shown in Table 2. The three dependent variables encompass EE%, PS, and ZP, as detailed in Table 2. The optimization was performed using a multi-response desirability function considering, EE%, PS, and ZP simultaneously to determine the most desirable formulation. The experimental design was evaluated using ANOVA, with *p*-values below 0.05 and F-values greater than 0.05 consider significant^42^. Preparation responses were analyzed via three mathematical polynomial models: linear, two-factor interaction (2FI), and quadratic models. The optimal model was identified through ANOVA metrics, particularly R² values (predicted and adjusted) and adequate precision (> 4). A close correspondence between predicted and adjusted R² (difference < 0.2) validated the model, while a coefficient of variation (CV% < 10%) confirmed reproducibility^43^.

**Table 1:** Composition of SLNs developed using 3^3^ Box–Behnken design (BBD).

Variables in their actual values			
Trials	A: Stearic acid	B: Span 80	C: Tween 80
	Grams	Grams	Grams
T1	2.45	2.45	0.25
T2	2.45	0.9	0.1
T3	4	2.45	0.4
T4	2.45	4	0.1
T5	2.45	2.45	0.25
T6	4	4	0.25
T7	0.9	0.9	0.25
T8	2.45	2.45	0.25
T9	0.9	2.45	0.4
T10	4	2.45	0.1
T11	2.45	0.9	0.4
T12	4	0.9	0.25
T13	0.9	2.45	0.1
T14	0.9	4	0.25
T15	2.45	4	0.4

**Table 2:** Independent and dependent variables for the BBD methodology utilized for the preparation of MOX SLNs.

Independent variable	Levels			Dependent variable
	Low (-1)	Medium (0)	High (1)	
A: Stearic acid (grams)	0.9	2.25	4	Y1: EE%
B: Span 80 (grams)	0.9	2.25	4	Y2: PS
C: Tween 80 (grams)	0.1	0.25	0.4	Y3: ZP

#### Development of MOX-SLNs through double emulsion method

**Fig. 1:**
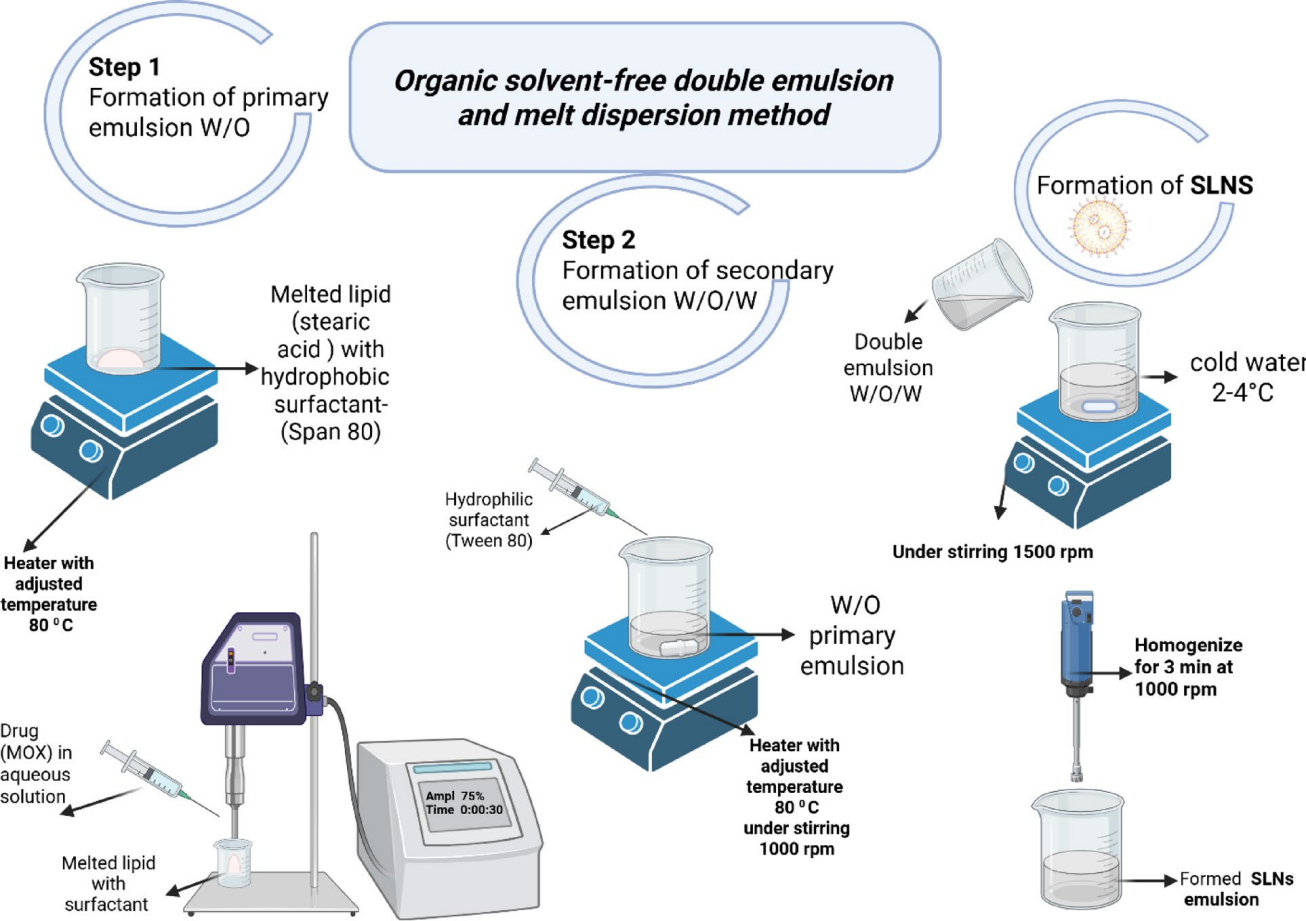
Schematic diagram illustrating the double emulsion organic solvent-free method for SLNs preparation.

To effectively encapsulate MOX’s aqueous phase in a lipid matrix under safe and mild processing conditions, a solvent-free double emulsion process was employed. Based on known limitations of other methods like solvent evaporation and single-emulsion approaches to hydrophilic drugs such as low recovery and phase separation prompted this choice. The solvent-free nature decreases residual solvent toxicity and reduces drug loss due to solubility in organic phases, making it more suitable for a topical delivery system employed in the treatment of chronic wounds^44^. Furthermore, the process enhances reproducibility and nanoparticle stability as well as the application of green formulation principles^22,45,46^. Accordingly, double emulsion organic solvent-free technique was applied to prepare the fifteen trials with the following detailed steps. Initially the first emulsion (w/o) was prepared by melting the calculated amount of solid lipid (stearic acid) above its melting point at 70°c and mixed with the determined amount of Span 80^30^. Then the aqueous phase containing the drug was added dropwise to this mixture under probe sonicator (Sonics Vibra Cell power 130 W, frequency 20 kHz, made by Sonics & Materials Inc, Newtown, CT) with amplitude 75% for 30 seconds. The amount of drug was fixed across all trials, and a primary w/o emulsion was formed. The secondary emulsion was then prepared through adding a predetermined amount of Tween 80 ,in form of aqueous solution^47^, dropwise under stirring at 1000 rpm for 30 min using hot plate magnetic stirrer (MSH-20D, DAIHAN Scientific, Korea). Finally, this formed w/o/w emulsion was poured into cold water (2–5 °C) with a ratio of 1:20 under stirring then this mixture was homogenized for 3 min at 1000 rpm using homogenizer (WiseTis HG-15D, Daihan Scientific, Korea)^48^. The formed SLNs went through centrifugation in a cooling centrifuge (Centurion Ltd., PRO-Research K241R, United Kingdom) at 15,000 rpm and 4 °C for a duration of 2 h. The supernatant was collected to further assess drug entrapment efficiency. Figure 1 illustrates the schematic representation of the method mentioned above.

### Characterization of formed MOX-SLNs

#### Entrapment efficiency (EE%)

An indirect method was used to determine the amount of unentrapped drug in the supernatant after centrifugation using cooling centrifuge (Centurion Ltd., PRO-Research K241R, United Kingdom). The absorbance of the supernatant was measured at predetermined MOX λ_max_ (293 nm), and the resulted absorbance was substituted into the predetermined calibration equation (y = 0.0985x − 0.0507) to calculate the drug concentration. The exact conditions were applied to develop blank nanoparticles, and their supernatant layer was used as control. The EE % was then calculated using the following equation^49^.

1 $${\text{EE }}\% = \frac{{{\text{Total amount of drug}} - {\text{unentrapped}}}}{{{\text{Total amount of drug}}}} \times 100$$

#### Analysis of particle size and zeta potential

Zetasizer (Nano ZS, Malvern, UK) was used to measure the average PS &ZP. These were essential features to detect the physical stability of the system^50^. All measurements were performed at room temperature (25 °C) and sufficient dilution was done using water for all dispersions^51^. Size measurements were conducted using Dynamic Light Scattering (DLS) technique, which involves assessing Brownian motion and correlating it to the dimensions of the particles. The ZP was determined by measuring Electrophoretic Mobility, which means the observed rate of migration of a component divided by the electric field strength in the specified medium.

### Optimization

All outcomes were optimized using Design Expert^®^ software (version 13) by applying certain constraints on the selected dependent variables. The optimization is based on maximizing ZP, EE% and minimizing PS. For predictive validation of the derived model, the optimized formula (F-opt) was again prepared then Freeze-dried in a lyophilizer (Alpha 1–2 LDplus, CHRIST, Germany) with the condenser maintained at -45 °C for 24 h to be analyzed for the above-mentioned parameters.

#### Assessment of F-opt

##### Entrapment efficiency %

The encapsulation efficiency was calculated for the prepared F-opt using the previously stated indirect method. Cold centrifugation was performed to separate the supernatant which was analyzed through UV spectrophotometer at predetermined (λ _max_).

##### Particle size and zeta potential

The PS &ZP of the F-opt were determined using Malvern zeta sizer, as described previously.

#####  Microscopical examination

TEM and SEM were used to examine morphology, nanoparticles preparation and surface characteristics of the F-opt. SEM imaging was conducted using a ThermoFisher Quattro S Field Emission Gun SEM (USA), and \the inner structure examination and high-resolution imaging were conducted on a Thermo Scientific™ Talos™ F200i S/TEM (20–200 kV), a field emission SEM.

#####  X-ray diffraction (XRD)

The crystalline or amorphous nature and phase composition of the pure MOX and Stearic acid, physical mixture (MOX: Stearic acid,1:1 W/W) and lyophilized F-opt, was analyzed using X- ray diffractometer (Panalytical Empyrean 3 diffractometer (Malvern, Netherlands)) equipped with a copper Kα radiation source (λ = 1.5406 Å). The scanning was conducted over the period of 37 min, over the range of 2θ = 4° to 90°, using a step size of 0.02° and a measurement time of 0.5 s per step. These conditions gave adequate resolution to resolve characteristic diffraction peaks as well as to determine variations in crystallinity among samples.

#####  Fourier transform infrared (FTIR) spectroscopy

FTIR was performed to verify if the chemical structure of MOX was preserved after encapsulation in lyophilized optimum SLNs and to evaluate potential drug-excipient interactions. The spectral analysis was conducted on a RAM II FT-Raman module coupled to a Vertex FTIR spectrometer (Bruker, Germany) in the wavelength range 500–4000 cm⁻¹ with high spectral resolution up to 0.1 cm⁻¹. The studies were conducted using an attenuated total reflectance (ATR) accessory, and spectra were measured with 16 cm⁻¹ resolution and a minimum scan rate of 50 scans per second.

## Results and discussion

### Ultraviolet (UV) spectrophotometry of MOX

**Fig. 2:**
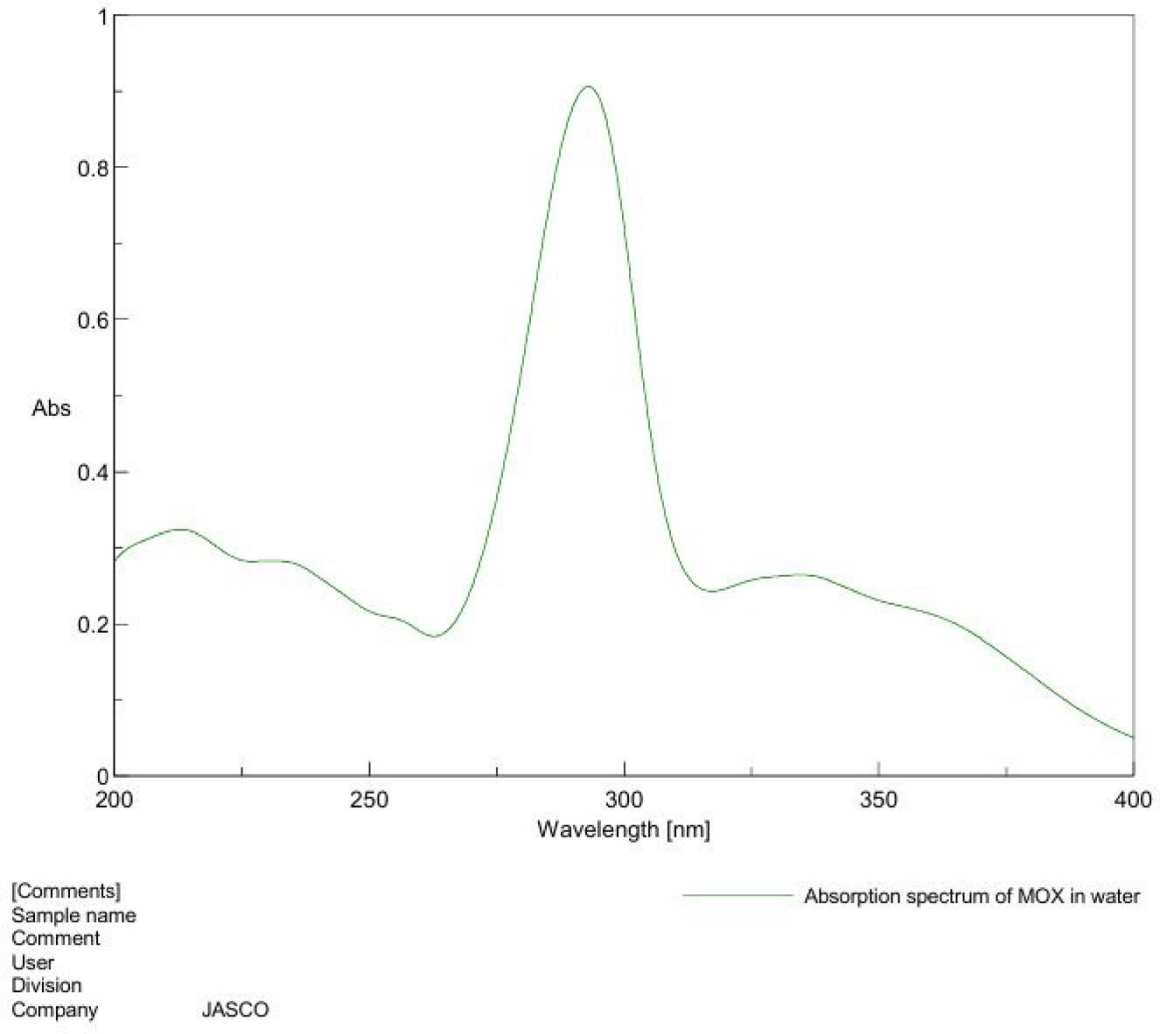
Absorption spectrum of MOX in distilled water.

As shown in Fig. 2, UV absorption spectra of MOX dissolved in distilled water exhibited a maximum absorbance (λ_max_) at 293 nm, this value was consistent with other published literature^52^.

### Selection of independent variables

During the preparation of all trials, few parameters were kept constant to ensure reproducible and consistent results. This included the MOX amount, sonication time and amplitude of probe (75% for 30 s), temperature (70 °C), agitation time for secondary emulsification (30 min), stirring speed (1000 rpm), prepared SLNs-to-cold-water ratio (1:20), and homogenization speed and time (3 min at 1000 rpm). As shown in Table 2, the only independent variables investigated systematically were solid lipid content and surfactant amounts and type. Stearic acid was selected as the type of solid lipid for preparation of nanoparticles because of its good biocompatibility and low toxicity in body, and it is commonly used in pharmaceutical formulations as lipid matrix to prepare solid nanoparticles^1^. Moreover, recent studies have indicated that stearic acid–based SLNs exhibit stable physicochemical properties for dermal use and enhanced skin penetration^53,54^. In the preliminary study, the aforementioned two forms of lecithin formed clumps that failed to melt even when the temperature was raised above the lipid’s melting point, leading to unsuccessful emulsion formation. Subsequently, Span 80 was evaluated and showed no phase separation, forming a more stable emulsion. This confirmed that nonionic surfactants were preferable for SLN preparation^55^.For the secondary phase, Poloxamer 80 and Tween 80 were tested at low concentrations; both led to less stable emulsions with phase separation. Poloxamer 80, even at higher concentrations, continued to produce phase-separated emulsions, whereas Tween 80 produced a homogeneous and stable emulsion at higher concentrations. Consequently, a combination of Span 80 and Tween 80 was deemed optimal due to their structural compatibility^55^. Moreover, for lipid nanoparticle preparations for topical use, the nonionic surfactants Tween 80 and Span 80 are generally selected owing to their appropriate hydrophilic–lipophilic balance (HLB)^56^ ,low irritation, and better stabilization of oil-in-water emulsions in skin formulations^57–60^. In this context, the external phase O/W was emulsified using Tween 80 (high HLB), whereas the primary W/O interface is emulsified with Span 80 (low HLB). The synergistic action of these surfactants minimized phase separation, reduced particle agglomeration, and enhanced complementary interfacial properties suitable for topical application^59,61,62^. Therefore, the MOX loaded SLNs were formulated through the combination of Span 80 and Tween 80 for complementary interfacial properties and topical compatibility, with stearic acid as a stable lipid matrix^63^.

### Statistical evaluation of BBD

Regression analysis was employed to model the responses into quadratic, two-factor interaction (2FI), and linear models. The responses were EE% (Y₁), PS (Y₂), and ZP (Y₃). The optimal model was selected based on the highest lack-of-fit value and the adjusted & predicted coefficient of determination (R²) from the respective models. In addition, analysis of variance (ANOVA) was performed to determine the statistically significant factors that influenced each response.

####  Impact of independent factors on EE% (Response 1-Y1)

The EE% of the developed SLNs ranged from 62.57 ± 2.91 in T14 to 91.96 ± 5.27 in T4, as shown in Table 3. The relatively high EE%, which exceeded 90%, may be attributed to the entrapment of the hydrophilic drug (MOX) in SLNs with stearic acid as the lipid matrix via the double emulsion method. This could be explained to the ability of stearic acid to form a stable lipid matrix that favors the encapsulation of hydrophilic drugs. Similar results were reported in a study using zidovudine as a hydrophilic drug, where stearic acid-coated SLNs prepared by the double emulsion solvent evaporation process exhibited high EE values^64^. Another contributing factor was that using stearic acid produced an imperfect crystalline matrix with enhanced drug encapsulation capability. This was supported by Subroto et al.^65^, who used the double emulsion technique to encapsulate ferrous sulfate, a water-soluble drug, using a combination of stearic acid and high-monolaurin fat. This approach led to extremely high EE% levels ranging from 99.97 to 99.99%.

**Table 3:** Mean values of EE%, PS and ZP for all trials.

Trials	Factor 1	Factor 2	Factor 3	Response 1 (Y1)	Response 2 (Y2)	Response 3 (Y3)
	A: Stearic acid	B: Span 80	C: Tween 80	Mean EE ± SD	Mean **PS ± SD**	Mean ZP ± SD
	Grams	Grams	Grams	%	Nm	mV
T1	2.45	2.45	0.25	88.10 ± 3.78	640.0 ± 20.0	− 58.1 ± 2.9
T2	2.45	0.9	0.1	80.14 ± 9.20	182.6 ± 6.5	− 29.6 ± 1.6
T3	4	2.45	0.4	90.35 ± 9.36	355.6 ± 15.5	− 54.4 ± 2.7
T4	2.45	4	0.1	91.96 ± 5.27	1020.0 ± 40.0	− 31.3 ± 1.5
T5	2.45	2.45	0.25	88.36 ± 10.83	917.9 ± 42.6	− 57.3 ± 2.9
T6	4	4	0.25	89.70 ± 6.12	1425.0 ± 55.0	− 29.8 ± 1.7
T7	0.9	0.9	0.25	70.52 ± 13.94	290.0 ± 15.0	− 30.9 ± 1.6
T8	2.45	2.45	0.25	75.19 ± 7.17	642.2 ± 22.7	− 59.0 ± 3.0
T9	0.9	2.45	0.4	71.11 ± 5.72	694.9 ± 25.0	− 44.6 ± 2.2
T10	4	2.45	0.1	87.31 ± 4.53	742.3 ± 32.5	− 33.1 ± 1.6
T11	2.45	0.9	0.4	70.83 ± 11.62	360.0 ± 20.0	− 38.0 ± 1.9
T12	4	0.9	0.25	74.04 ± 10.30	420.2 ± 15.0	− 48.3 ± 2.5
T13	0.9	2.45	0.1	79.08 ± 10.12	230.1 ± 15.0	− 45.2 ± 2.2
T14	0.9	4	0.25	62.57 ± 2.91	415.4 ± 15.0	− 45.4 ± 2.3
T15	2.45	4	0.4	81.11 ± 6.70	210.0 ± 10.0	− 36.8 ± 1.8

The Linear model was identified as the most significant unaliased model for the analysis of entrapment efficiency, as indicated in Table 4, with a *p*-value of 0.0337 and an R² value of 0.5319. The lack-of-fit in the model was insignificant compared with the pure error, as indicated by the *p*-value of 0.6544, which confirmed the adequacy of the model fit. A significant linear relationship among the independent and dependent variables was clearly indicated by the lack-of-fit, which was not significant (*p* > 0.05). Linear models’ relatively higher values of the predicted and adjusted R² confirm the model to be appropriate for the data 36. A final model equation for EE% was as follows:

2 $${\text{EE}}\% = {\text{ }} + {\text{8}}0.0{\text{2}} + {\text{7}}.{\text{27A }} + {\text{3}}.{\text{73 B }} - {\text{3}}.{\text{14C}}$$

**Table 4:** Proposed model for observed EE%.

Source	F-value	*P*-value	
Linear vs. Mean	4.17	0.0337	Suggested
2FI vs. Linear	1.23	0.3595	
Quadratic vs. 2FI	1.96	0.2389	
Cubic vs. Quadratic	0.3302	0.8093	Aliased

Table 5 showed the result of ANOVA for the final model. As indicated, the model was statistically significant, as the p-value was below 0.05. Furthermore, the data indicated that the drug EE% was statistically affected by factors A — the amount of stearic acid content —as the p-value for factor A was < 0.05, confirming statistically significance. Moreover, the high F-value indicated significant mean differences, confirming the strong contribution of this factor to the model.

**Table 5:** ANOVA for linear model (Response 1: entrapment efficiency).

Source	F-value	*p*-value	
Model	4.17	0.0337	Significant
A-Stearic acid	8.62	0.0135	
B-Span 80	2.27	0.1601	
C-Tween 80	1.61	0.2312	

**Fig. 3:**
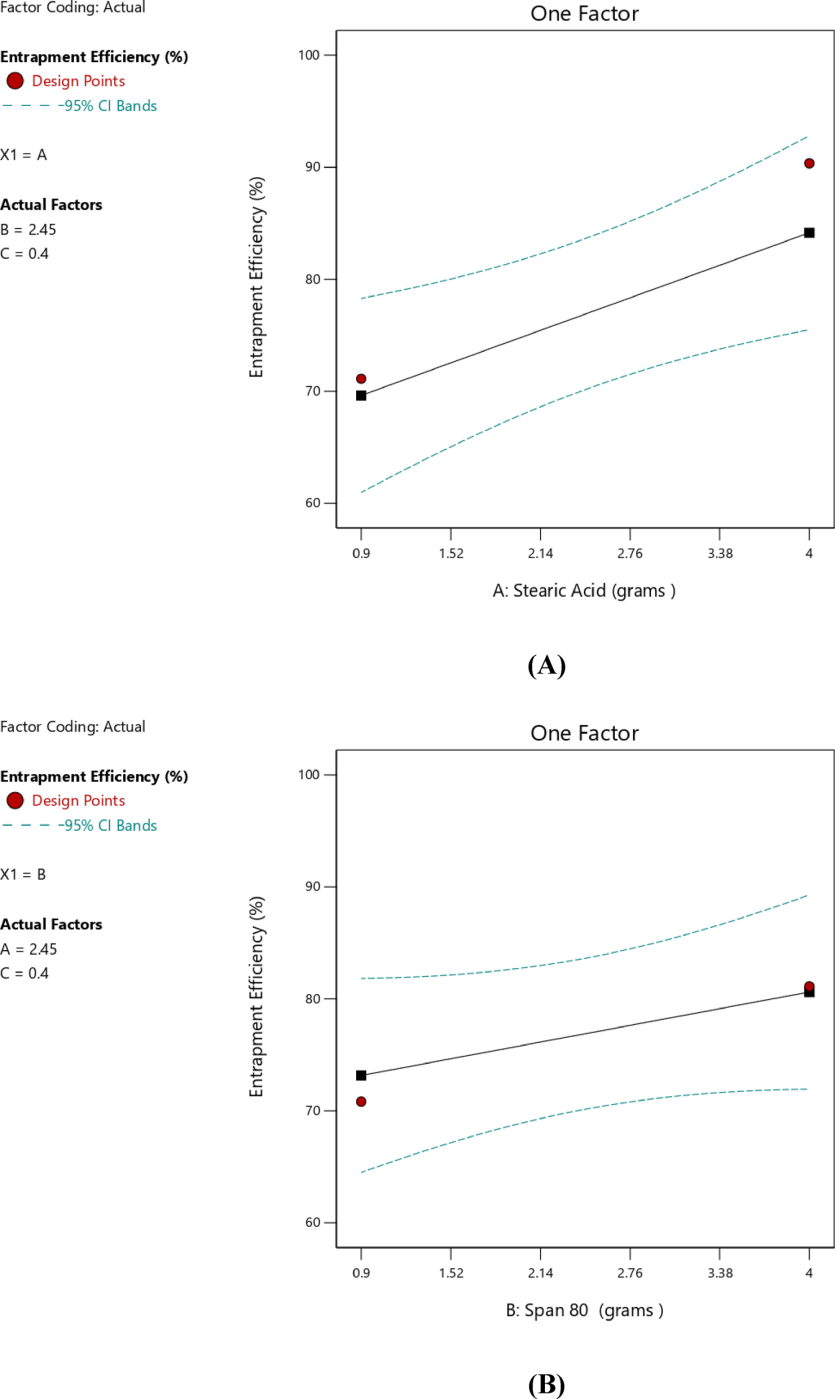

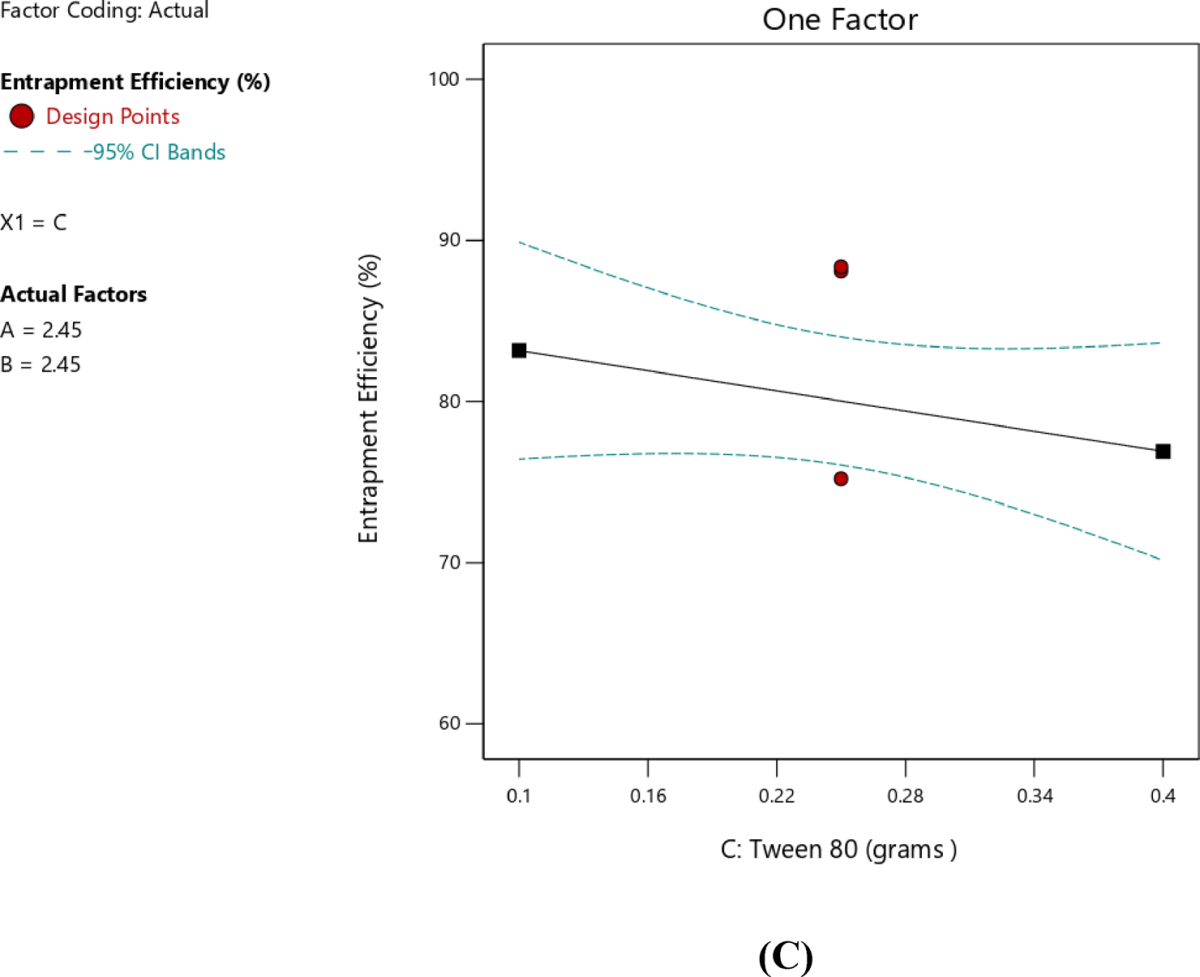
Line plot for the effect of stearic acid amount (**A**), Span 80 amount (**B**), and Tween 80 amount (**C**), on the EE%.

**Fig. 4:**
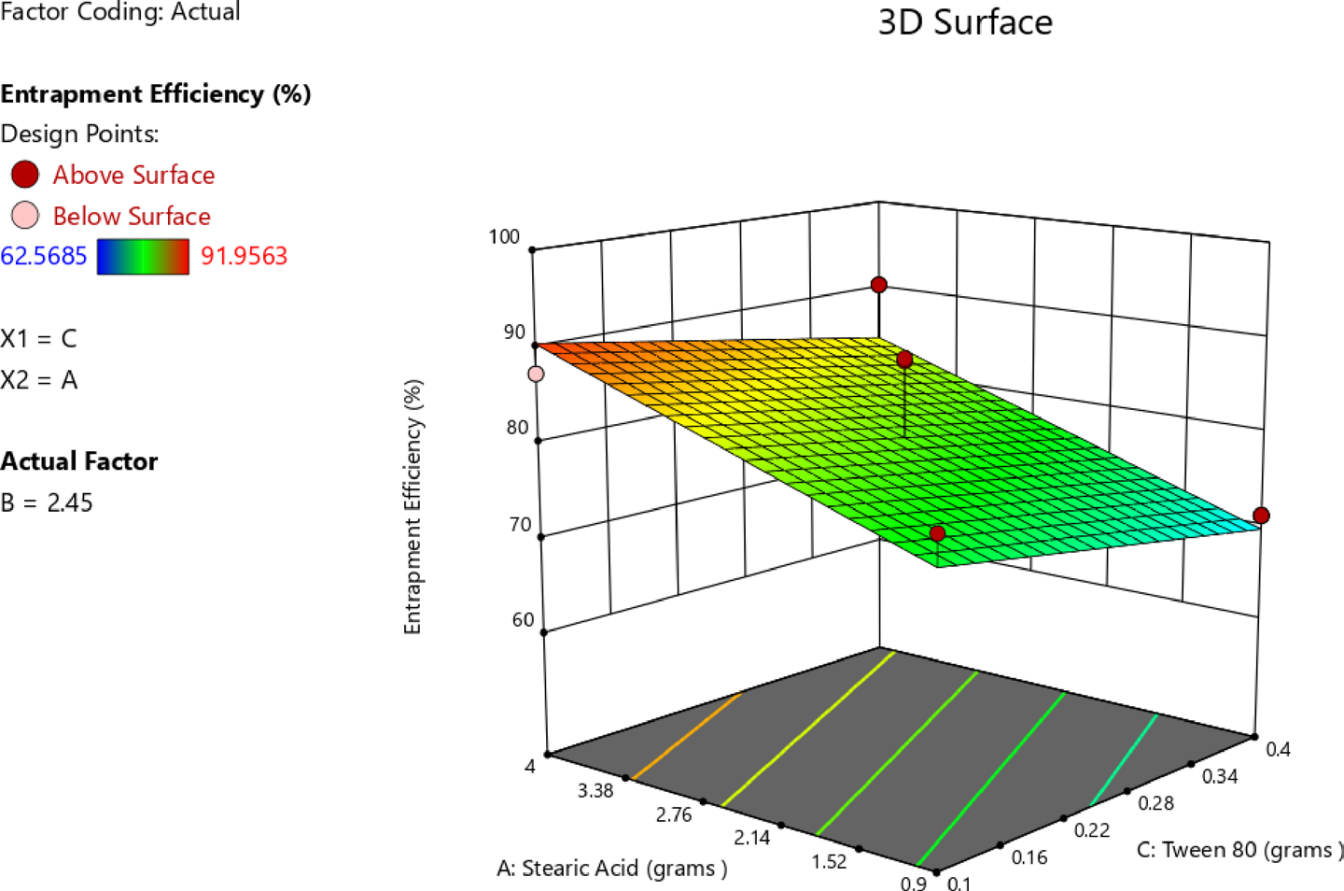
3D surface plot of main effect of stearic acid amount (A) and Tween 80 amount on EE%.

**Fig. 5:**
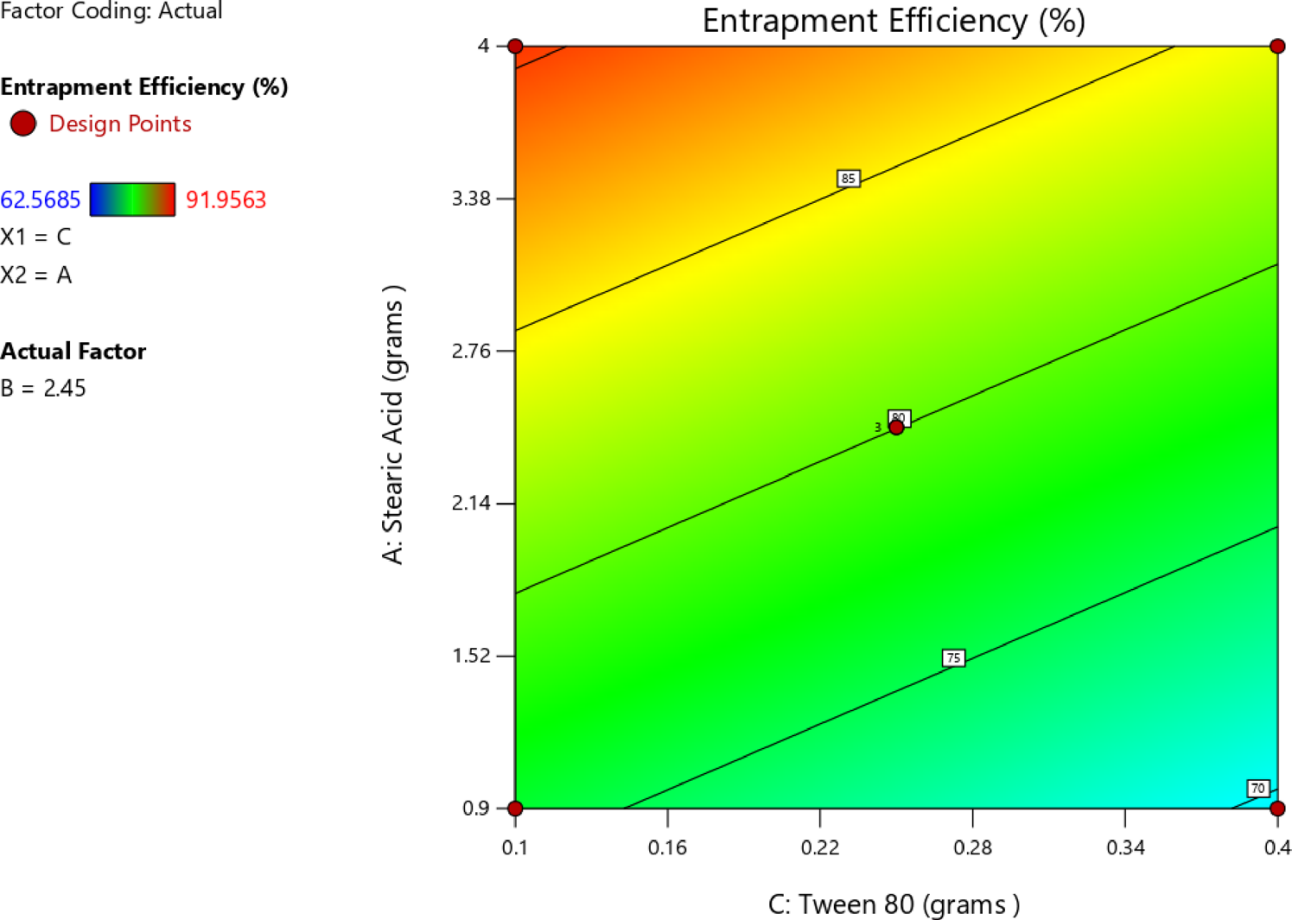
Contour plot of main effect of stearic acid amount and Tween 80 amount on EE%.

An increase in the amount of stearic acid was correlated with higher EE%, as indicated by the positive coefficient value reported in Table 6, suggesting a direct linear correlation with EE%. The one-factor plot shown in Fig. 3A also confirmed this trend. The coefficient estimate represented the expected change in the response variable was expected to change for every unit increase in the predictor variable, while all other variables were kept constant. In addition, T14, which was prepared with minimum amount of stearic acid (0.9 g), exhibited minimum value for EE% (62.57 ± 2.91), and this affirmed the reported correlation. The observations were in agreement with those of Silpa et al.^66^, who correlated higher EE% values with higher lipid content, which hindered the partitioning of MOX into the external aqueous phase during emulsification. During the process, a drug-enriched core was formed within the SLNs, thereby improving drug encapsulation. Similarly, Darsh et al.^67^, reported a direct proportionality between lipid content and EE%, where higher lipid content was reported to enhance the matrix capacity for drug loading. Moreover, the illustrated 3D surface plot in Fig. 4 and the contour plot in Fig. 5 confirmed the significant effect of stearic acid on EE%. The upward sloping surface towards the rear-right corner of the 3D plot corresponded to an increase in the stearic acid amount resulting in higher EE%. This observation was further supported by the gradual color gradient in the Fig. 5, which showed equal and complementary improvement in EE% with higher concentrations in stearic acid amount^68^. The positive estimate of the coefficient in Table 6 suggested a direct influence of Span 80 concentration on EE%; however, the effect was relatively small and not significant (p-value = 0.1601), as shown in Fig. 3B. Although Tween 80 did not have a statistically significant effect on EE% (*p* = 0.2312), the negative coefficient in Table 6 suggested a potential inverse trend between Tween 80 amount and EE%, as indicated in Fig. 3C. The increase in EE% not only reflects the rise in lipids but also in molecular interactions in the emulsion system. Amphiphilic balance between Span 80 and Tween 80 controls curvature and rigidity of the interfacial film enveloping the inner aqueous phase. Moderation in the concentration of Span 80 makes the interfacial layer thick, slowing down diffusion of the hydrophilic drug into the continuous phase on solidification. When the polyoxyethylene chains of Tween 80 molecules extend outwards, a close-packed steric barrier is created that immerses water droplets in the lipid matrix. The same cooperative effects were reported for improving drug retention^22^. The same trends were also obtained using hydrophilic antibiotics introduced into the lipid matrix, with surfactant mixtures reducing drug migration and enhancing interfacial packing^28^. Also, partial amorphization of the lipid phase—facilitated through trapping MOX molecules in crystal defects and later confirmed by XRD analysis—further improved encapsulation. This route of defect-assisted entrapment for hydrophilic drugs is well established in saturated lipid matrices^28^. Also, when loading ciprofloxacin with a solvent-free double-emulsion process, comparable enhancements were seen, indicating that rigid lipid matrices and properly tuned surface^14,69,70^.

**Table 6:** Coefficients in terms of coded factors regarding EE%.

Factor	Coefficient estimate	95% CI Low	95% CI High
Intercept	80.02	76.05	84.00
A-Stearic acid	7.27	1.82	12.71
B-Span 80	3.73	− 1.72	9.17
C-Tween 80	− 3.14	− 8.58	2.31

#### Impact of independent factors on PS (Response 2-Y2)

The efficiency of dermal drug delivery and tissue penetration was greatly influenced by the size of nanoparticles. Previous studies have shown that nanoparticles of approximately 80–100 nm can penetrated deeper into the skin, whereas particles of approximately 500–600 nm in diameter can provided better skin deposition^71^. These findings suggested that the optimal PS would most likely be determined by a compromise between drug residence within the skin layers and penetration depth. Although nanocrystals in the size range of 100–700 nm have historically been associated with enhanced delivery across the skin^72^, PS required optimization in this case to achieve these specific therapeutic outcomes^71,73^. Thus, the main target was to minimize the PS. As shown in Table 3, PS ranged from 182.6 ± 6.5 in T2 to 1425.0 ± 55.0 in T6. According to the data in Table 7, the significant model for the analysis of PS was the 2FI-two factor interaction, which represents a linear model with interaction terms for the formulation variables. The p-value was 0.0589 and an R² value of 0.7467. Moreover, the lack-of-fit of the model was insignificant compared with the pure error, as indicated by the p-value of 0.3141, confirming that the model was adequate to represent the experimental data. According to these findings, the 2FI model adequately captured the linear interactive relationships between the independent variables and particle size while avoiding overfitting associated with higher-order models. A significant relationship among the independent and dependent variables was clearly indicated by the lack-of-fit, which was not significant (*p* > 0.05). The model represented equation is:

3 $$\begin{aligned} {\text{PS }} = &\, + {\text{569}}.{\text{86}} + {\text{ 163}}.{\text{975 A}} + {\text{ 227}}.{\text{2125 B}} - {\text{ 69}}.{\text{2625 C}} \\ \quad & + {\text{ 219}}.{\text{575 AB}} - {\text{ 212}}.{\text{625 AC}} - {\text{246}}.{\text{8BC}} \\ \end{aligned}$$

**Table 7:** Proposed model for observed PS.

Source	F-value	*p*-value	
Mean vs. Total			
Linear vs. Mean	2.32	0.1316	
2FI vs. Linear	3.78	0.0589	Suggested
Quadratic vs. 2FI	1.17	0.4090	
Cubic vs. Quadratic	2.65	0.2856	Aliased

ANOVA results for 2FI model used to evaluate PS were presented in Table 8. The overall model significance was confirmed by a p-value of 0.0394 (*p* < 0.05). Additionally, the relatively high F-value of 3.93 for the overall model suggested the existence of significant mean differences. The fitness of the model to reproduce the experimental data was also supported by the non-significant lack-of-fit (*p* = 0.3141). Among the individual factors, Span 80 (factor B) had the most pronounced on PS, as evidenced by its *p*-value of 0.0249.

**Table 8:** ANOVA for 2FI model (Response 2: PS).

Source	F-value	*P*-value	
Model	3.93	0.0394	Significant
A-Stearic Acid	3.95	0.0821	
B-Span 80	7.59	0.0249	
C-Tween 80	0.7049	0.4255	
AB	3.54	0.0966	
AC	3.32	0.1058	
BC	4.48	0.0673	
Lack of fit	2.49	0.3141	Not significant

**Fig. 6:**
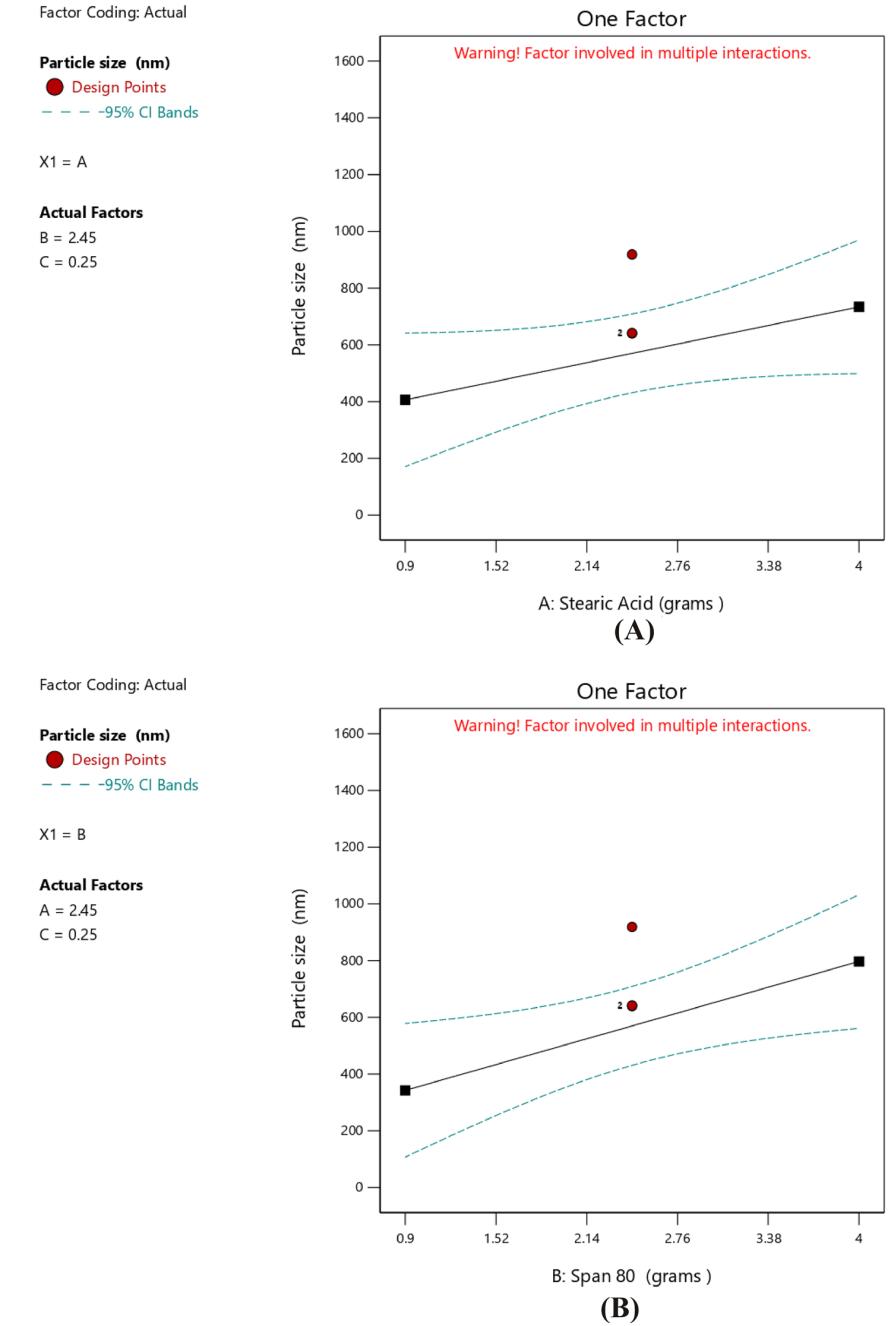

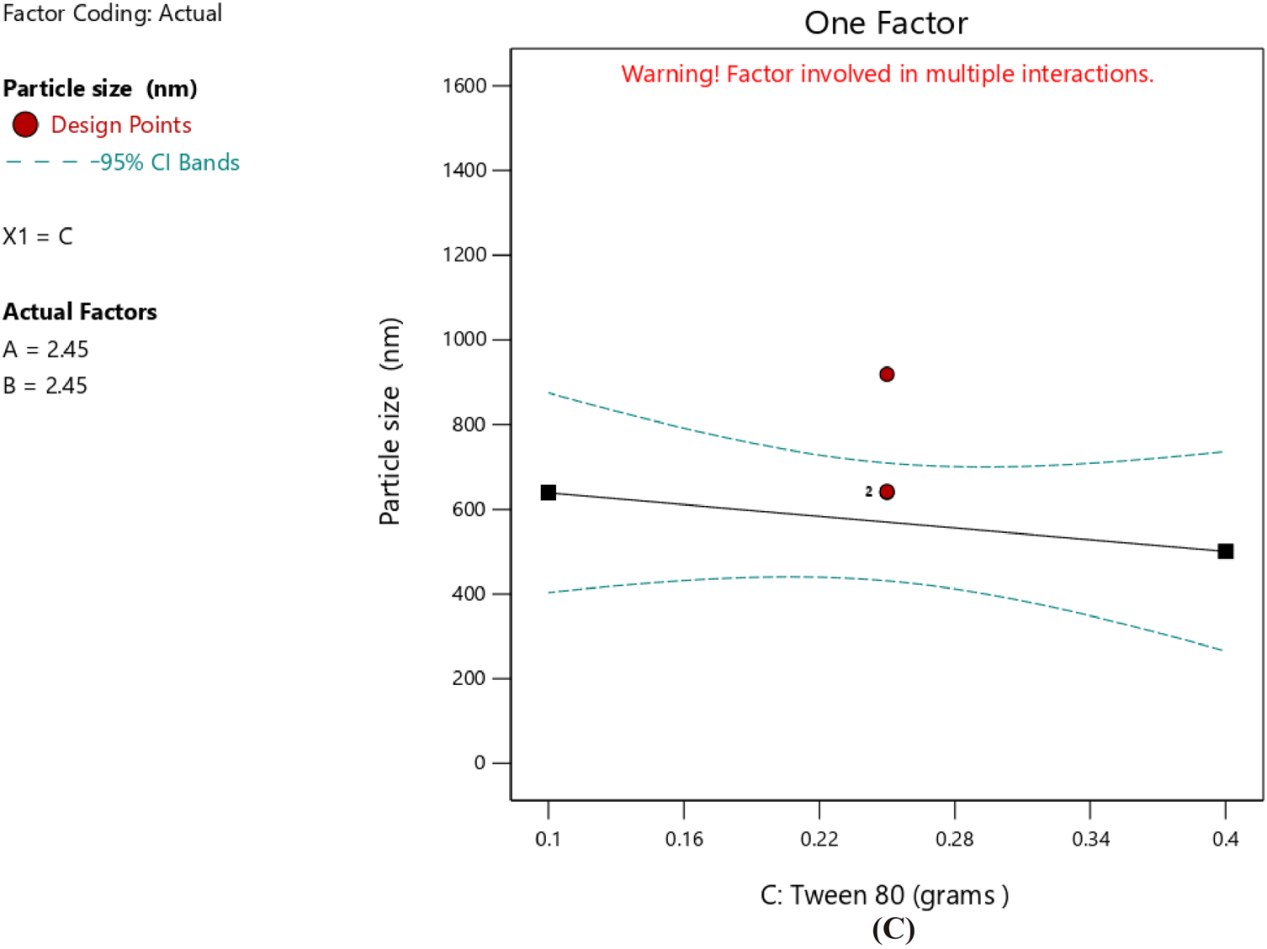
Line plot for the effect of Stearic acid amount (**A**), Span 80 amount (**B**), and Tween 80 amount (**C**), on the PS.

**Fig. 7:**
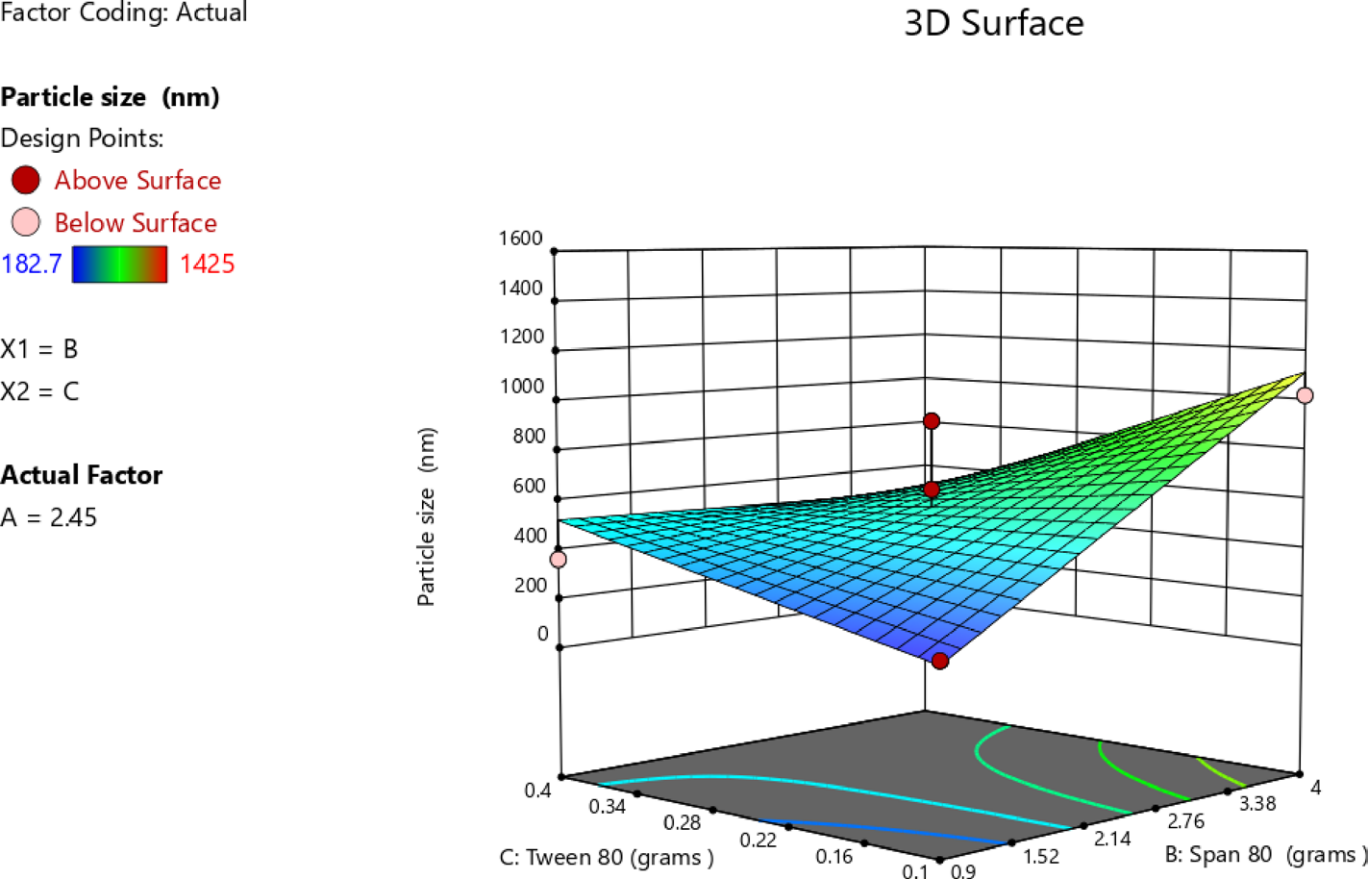
The 3D surface plot of main effect of Span 80 amount and Tween 80 amount on PS.

**Fig. 8:**
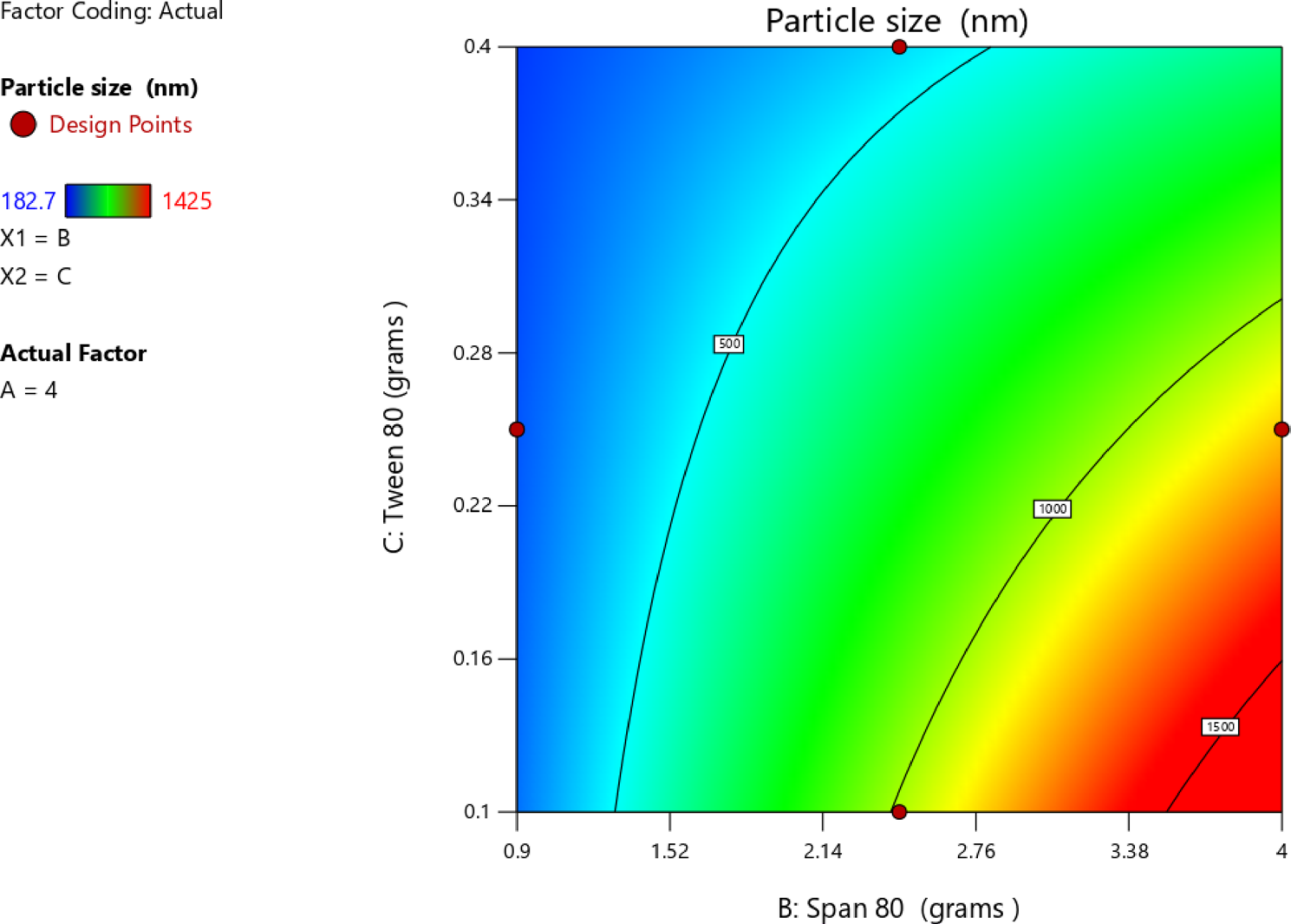
Contour plot of main effect of Tween 80 amount and Span 80 amount on PS.

As indicated in Table 9, the main impact of stearic acid (A) had a positive coefficient of + 163.97; nevertheless, its partial contribution was not statistically important considering the broad confidence interval (− 26.26 to 354.21), as shown in Fig. 6C. Moreover, Span 80 exhibited a positive main effect of + 227.21 with a confidence interval that did not include zero, indicating its significant contribution in enhancing PS. This was confirmed by the trend apparent in the one-factor plot in Fig. 6B and the three-dimensional surface plot displayed a high degree of upward curvature along the Span 80 axis, which demonstrated that higher levels of Span 80 were associated with a substantial rise in PS (Fig. 7). Moreover, the contour diagram in Figure 8 aided in illustrating the interaction between Span 80 and PS as shown, the color scheme ranges from blue, for small particles, to red thereby graphically confirming that PS increased proportionally with the amount of Span 80. This may have been attributed to the low HLB value (~ 4.3) of Span 80, which results in less stable emulsions with reduced steric stabilization and less interfacial tension control. According to Shahraeini et al.^74^, drug molecules tended to diffuse out of smaller particles with larger surface areas. An increase in Span 80 thus decreased the overall HLB of the surfactant system, thereby promoting particle growth. Similarly, it was reported that surfactants with higher HLB values such as Poloxamer 188 (HLB ~ 29) created much smaller nanoparticles compared to those with lower HLB values, likely due to their greater emulsification efficiency and steric stabilization capacity^75^. Furthermore, data from another study^76^ revealed that PS growth was obvious when the HLB value was reduced below 10.06. Although the effect of Tween 80 was not statistically significant, its negative main effect (-69.26), presented in Table 9 and presented in Fig. 6A, indicated a potential ability to reduce PS. From a mechanistic standpoint, particle-size distribution formed is controlled by the interaction between interfacial elasticity of the surfactants and viscous stress of the lipid phase. Internal-phase viscosity is increased with higher concentrations of stearic acid, which reduces cavitation efficacy and disruption of droplets under sonication^77^. Increasing concentrations of Tween 80, on the other hand, produce smaller emulsion droplets, which are compressed into nanoparticles of smaller size by reducing interfacial tension and increasing film flexibility^78^. Span 80, with its low HLB and limited hydration potential that predispose droplet coalescence on cooling, induces very positive effects on particle size consistent with dynamic-emulsification theory that proposes particle size to be dictated by interfacial-viscous force equilibrium^79^. A recent study^80^ demonstrated that the viscosity–tension interaction brought about by altering the lipid–surfactant ratio exposed immediate effect on SLN size and homogeneity. The same was noted with stearic-acid SLNs stabilized with Span 80/Tween 80, in which interfacial stiffness produced larger particles at increased Span 80^81^. Therefore, the current particle-size data support the effectiveness of dual-surfactant emulsification to control particle size and are consistent with theoretical and empirical models of viscosity–interfacial-tension competition in nanoparticle formation. The quadratic model illustrates how electrostatic and steric forces cooperate on the nanoparticle surface to control the surface-charge behavior^82^.

**Table 9:** Coefficients in terms of coded factors regarding EE%.

Factor	Coefficient estimate	95% CI low	95% CI high
Intercept	569.86	430.93	708.79
A-Stearic Acid	163.97	− 26.26	354.21
B-Span 80	227.21	36.98	417.45
C-Tween 80	− 69.26	− 259.50	120.97
AB	219.58	− 49.46	488.61
AC	− 212.62	− 481.66	56.41
BC	− 246.80	− 515.83	22.23

#### Impact of independent factors on ZP (Response 3-Y3)

ZP values indicated the stability of the formed solid nanoparticles. The higher the ZP (whether positive or negative), the stronger the electrostatic repulsion, and consequently better stability^83^. The charge presented on the surface of nanoparticles depended on the nature of the ingredients used in their preparation. The zeta potential of the prepared nanoparticles ranged from − 29.6 ± 1.6 in T2 to -59.0 ± 3.0 in T8 as shown in Table 3. This negative charge was attributed to the use of stearic acid as the solid lipid in the preparation. In aqueous solution, stearic acid dissociated, resulting in negatively charged particles. This occurred because the stearic acid structure has a terminal carboxylic group (–COOH) that gets ionized to (–COO^−^) in aqueous media^65,84,85^. The best fitting model for ZP analysis was the quadratic model, as illustrated in Table 10. The *p*-value was < 0.0001 and an R² value of 0.9928. The lack-of-fit of the model was insignificant compared to the pure error, as indicated by the p-value of 0.1773. A significant relationship among the independent and dependent variables was clearly indicated by the lack-of-fit, which was not significant (*p* > 0.05). Furthermore, the lack-of-fit *p*-value of 0.1773 (> 0.05) confirmed the model adequacy and significantly. The regression equation of the model was as follows

4 $$\begin{aligned} {\text{ZP }} = & {\text{ }} - {\text{58}}.{\text{133 }} + {\text{ }}0.0{\text{62A}} + {\text{ }}0.{\text{437B }} - {\text{4}}.{\text{325C }} + {\text{ 8}}.{\text{25AB }} \\ \quad & - {\text{5}}.{\text{475AC }} + {\text{ }}0.{\text{725BC }} + {\text{ 4}}.{\text{565A}}^{{\text{2}}} {\text{ }} + {\text{ 14}}.{\text{967B}}^{{\text{2}}} + {\text{ 9}}.{\text{242C}}^{{\text{2}}} \\ \end{aligned}$$

**Table 10:** Proposed model for observed ZP.

Source	F-value	*P*-value	
Mean vs. Total			
Linear vs. Mean	0.3681	0.7776	
2FI vs. Linear	0.9454	0.4631	
Quadratic vs. 2FI	154.63	< 0.0001	Suggested
Cubic vs. Quadratic	4.80	0.1773	Aliased

The data in Table 11 indicated the ANOVA results of the model. The* p*-value (< 0.0001) was below 0.05, confirming the statistically significant of the model. In this case, C, AB, AC, A², B², and C² were significant model terms. Values greater than 0.1000 indicated the model terms were not significant. Furthermore, the F-value of 4.80 implied that the lack-of-fit was not significant relative to the pure error.

**Table 11:** ANOVA for quadratic model (Response 3: ZP).

Source	F-value	*p*-value	
Model	77.10	< 0.0001	Significant
A-stearic acid	0.0132	0.9131	
B-Span 80	0.6456	0.4582	
C-Tween 80	63.09	0.0005	
AB	114.78	0.0001	
AC	50.55	0.0009	
BC	0.8864	0.3897	
A²	32.46	0.0023	
B²	348.71	< 0.0001	
C²	132.96	< 0.0001	
Residual			
Lack of fit	4.80	0.1773	Not significant

**Fig. 9:**
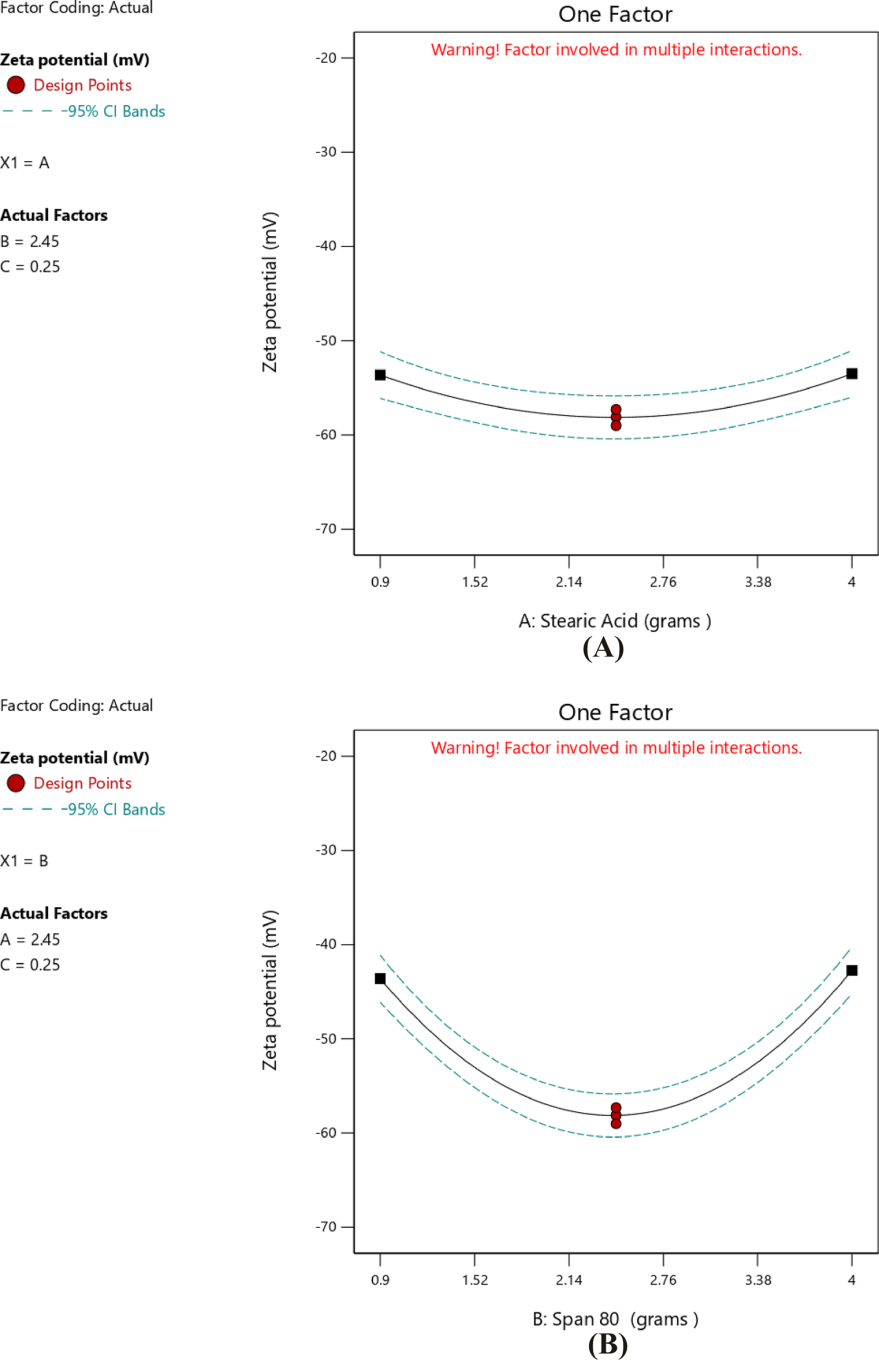

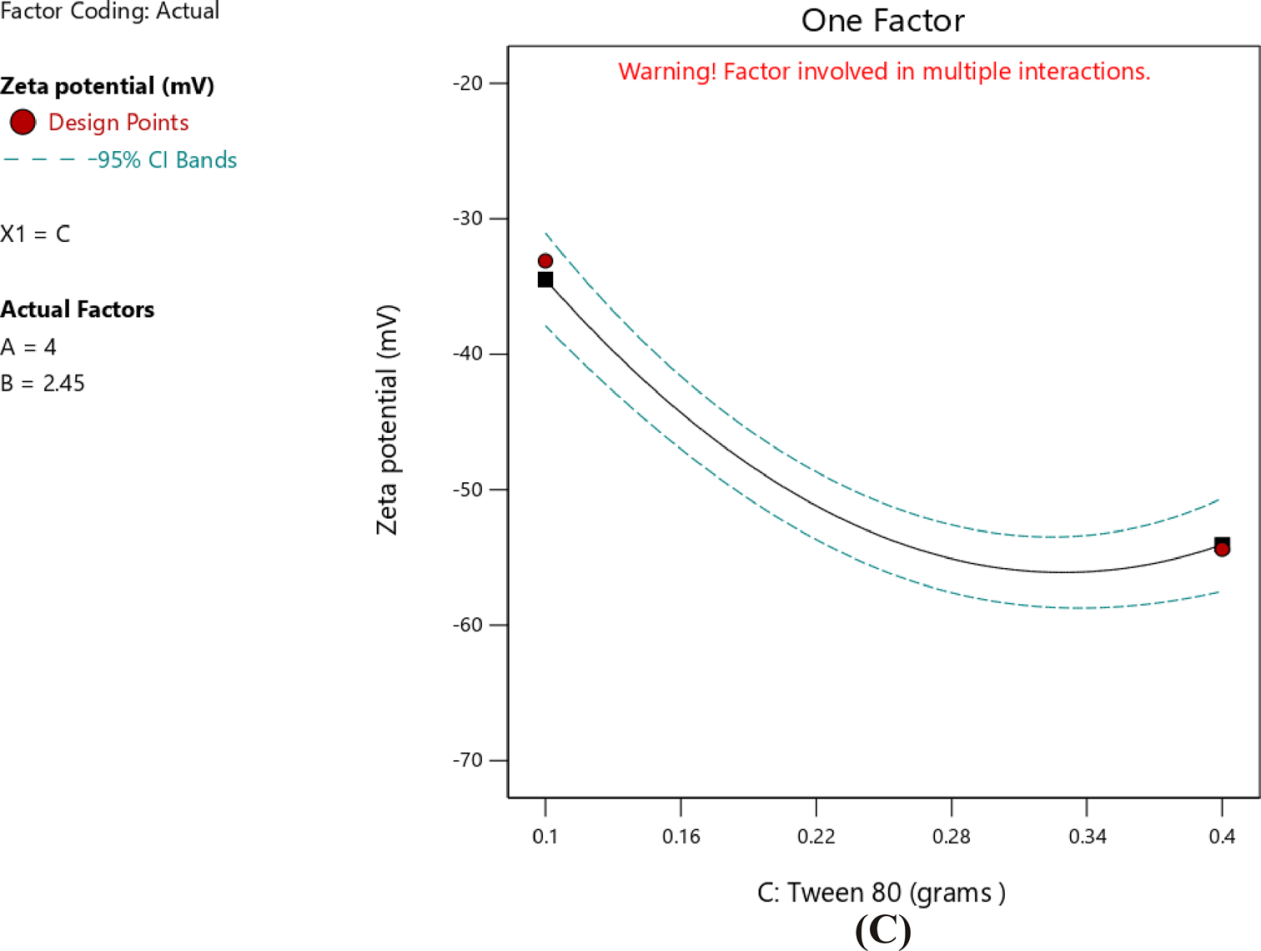
Line plot for the effect of stearic acid amount (**A**), Span 80 amount (**B**), and Tween 80 amount (**C**) on ZP.

**Fig. 10:**
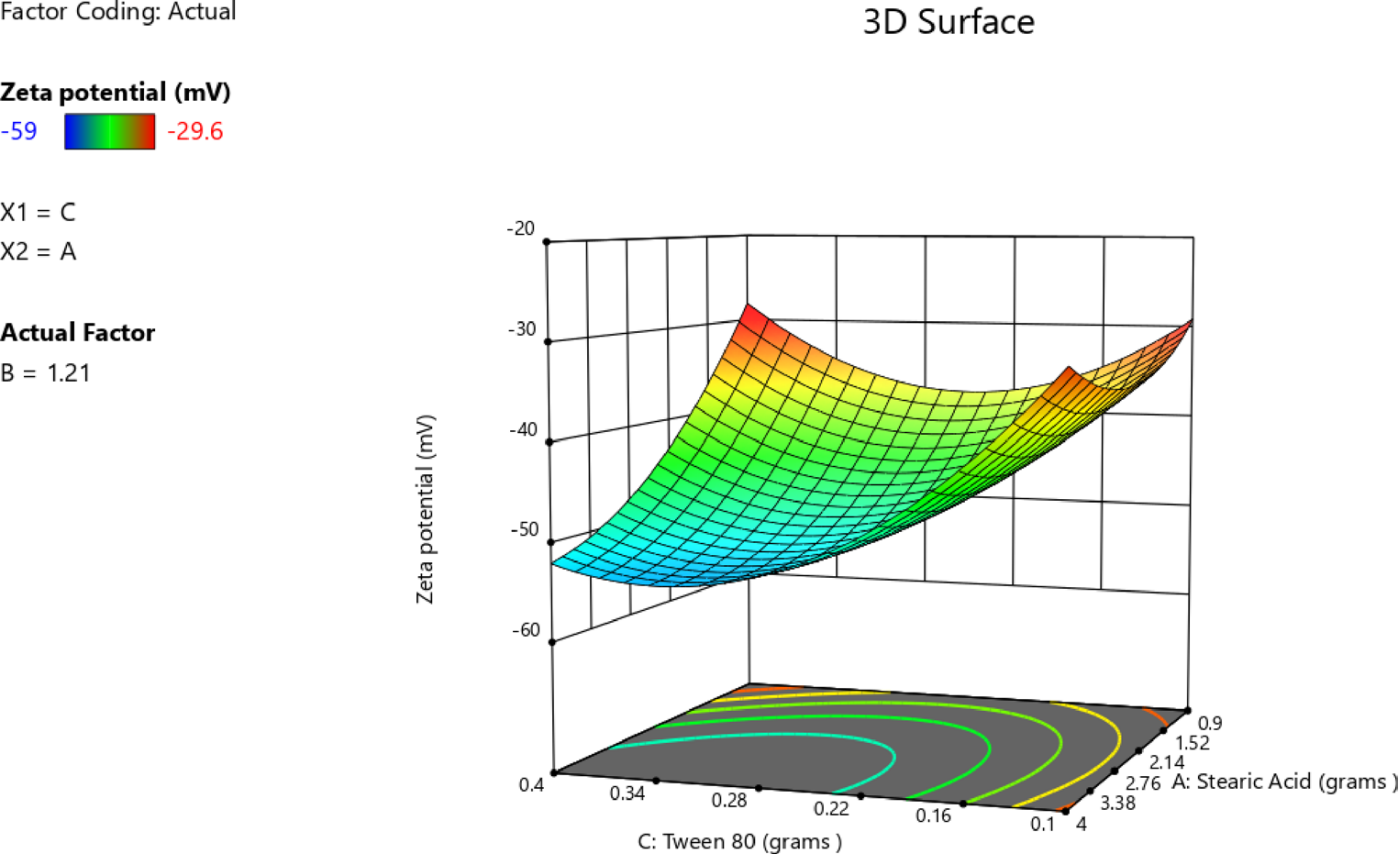
3D surface plot of main effect of stearic acid amount and Tween 80 amount on ZP.

**Fig. 11:**
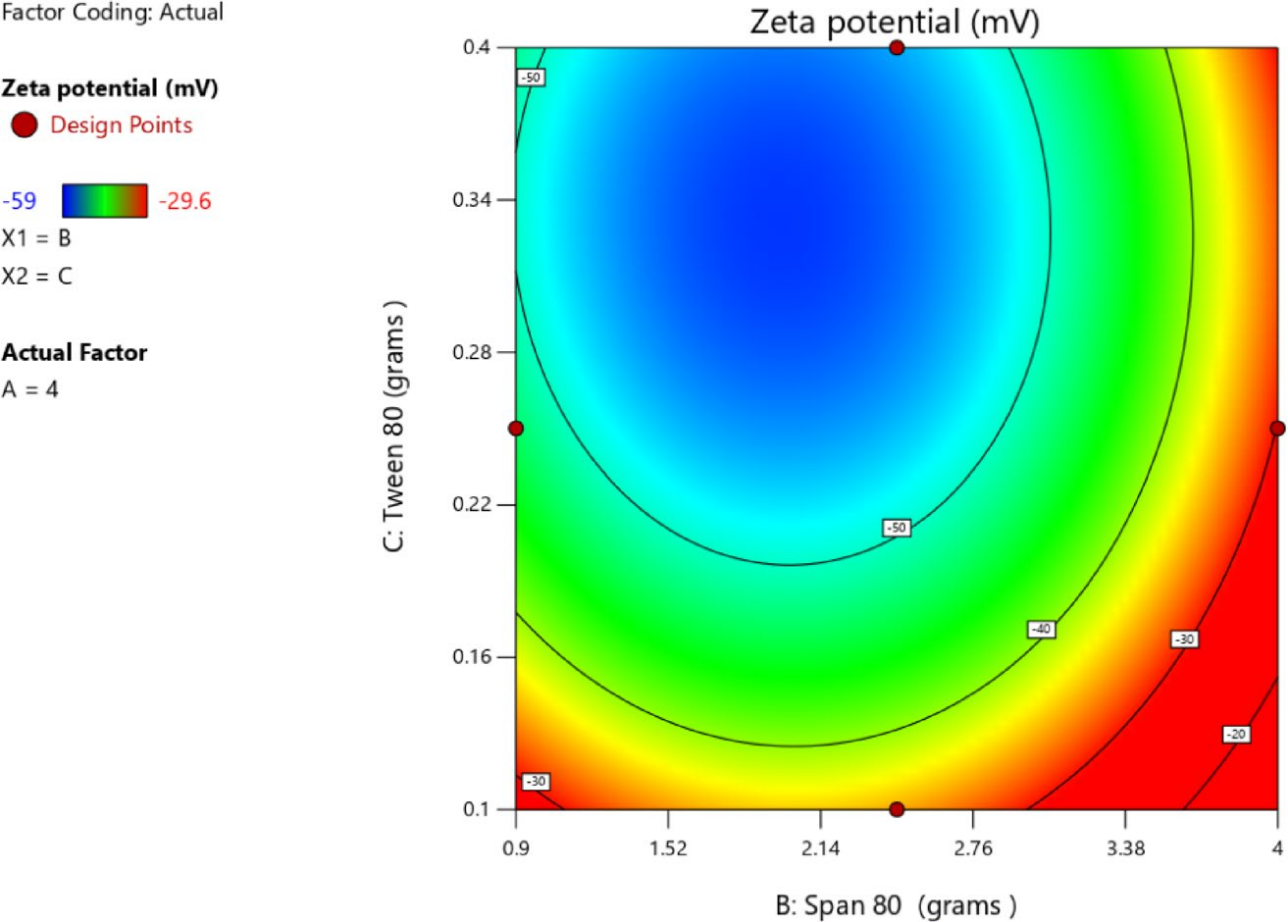
Contour plot of main effect of Tween 80 amount and Span 80 amount on ZP.

The correlation between the stearic acid amount and the ZP was a nonlinear relationship. Although the linear coefficient with respect to stearic acid was statistically not significant, the response curvature was confirmed by the existence of a large quadratic term, as indicated in Table 12. The linear term A was + 0.0625 and CI − 1.34 to 1.46 thus insignificant, but the quadratic term A² was + 4.57 and CI 2.51 to 6.63 hence significant and confirmed curved response as illustrated in Fig. 9A. The graph showed that ZP initially became increasingly negative with increasing stearic acid amount to a minimum prior to increasing again at higher amount of stearic acid thus forming a U-shaped curve. This indicated that intermediate concentrations of stearic acid result in the most negative values of ZP, which were the optimal for stability. A study confirmed that the relationship between the amount of stearic acid and ZP value was not linear^77^, stearic acid stabilizes the particles electrostatically by adsorption onto the surface of SLNs, and any deficiency or excess will disturb this equilibrium, wherein ZP was lowered this was an indicator for losing colloidal stability^86^. Similar to the effect of stearic acid, Span 80 exhibited a quadratic effect on ZP (Table 12). Its highly significant non-linear term indicated the sharp U-shaped correlation where the surface charge of nanoparticles was less negative at both low and high concentrations and reached its most negative value at intermediate levels as shown in Fig. 9B. This was consistent with the findings of Rostamkalaei et al.^76^, who found that The ZP of SLNs was usually decreased by increasing the concentration of Span 80. Because of the role of Span 80 which enhanced surface charge by producing a more compact surfactant layer around the nanoparticles, reduction of the negative ZP may occur when amounts of Span 80 were above the optimum at intermediate levels. This could potentially reduce the stability of the stabilizing surfactant layer and consequently decrease colloidal stability. Furthermore, Tween 80 exerted the most influence on ZP among the three independent variables. The linear term had a highly negative coefficient, indicating that higher Tween 80 amount led to more negative ZP and, thus, better stabilization of the colloid (Table 12). This finding was further confirmed by the 3D structure in Fig. 10 and in contour. plot in Fig. 11. Furthermore, the presence of a significant quadratic term indicated that the relationship was not linear in the sense that ZP initially reached a maximum value before it falls at higher concentrations. This behavior was shown in the accompanying Fig. 9C with a lower minimum compared with that of stearic acid and Span 80, with a characteristic curved response. Stearic acid also exhibited a notable negative interaction with Tween 80, indicative of a strong interactive effect^87^. Collectively, these findings demonstrated the critical role played by Tween 80 in the modulation of surface charge and the conferment of stability to the nanoparticles produced. This aligned with Shi et al.^28^ findings that increasing the amount of Tween 80 increased the ZP values. This might have been attributed to the hydrophobic chain length of Tween 80, compared with other Tweens, which led to more compact packaging of hydrophobic chains in the oil phase. Upon addition of the microemulsion to cold water, the oil phase became the solid lipid matrix, as in SLNs. Denser packing of these hydrophobic chains yielded a more rigid lipid core. Hence, Tween 80 enhanced the structural rigidity and overall stability of the SLNs. Moreover, the complex surface-charge behavior of the quadratic model shows how steric and electrostatic forces interact at the nanoparticle interface. With intermediate concentrations of stearic acid, the maximum number of ionized carboxylate groups (–COO⁻) is established, which yields maximum colloidal stability and repulsive interactions. When lipid content exceeds the optimum concentration, active sites are occupied with non-ionized lipid, causing dilution of the charge^88^. Span 80 likewise influenced interfacial dipole arrangement: below a certain level, there was insufficient coverage so charge inhomogeneity occurred; at higher Span 80 levels, a bulky steric layer that helped to sustain surface potential existed^89^. Tween 80 stabilized the system likewise by taking on its ethoxy head groups to create an extended hydration layer, expanding the thickness of the electrical double layer and preventing aggregation. Fluoroquinolone-loaded SLNs, which had zeta potentials of around − 50 mV, indicated similar electro-steric stabilization, highlighting that well-adjusted surfactant ratio enhanced colloidal stability.

**Table 12:** Coefficients in terms of coded factors regarding ZP.

Factor	Coefficient estimate	95% CI low	95% CI high
Intercept	− 58.13	− 60.42	− 55.85
A-Stearic acid	0.0625	− 1.34	1.46
B-Span 80	0.4375	− 0.9622	1.84
C-Tween 80	− 4.33	− 5.72	− 2.93
AB	8.25	6.27	10.23
AC	− 5.48	− 7.45	− 3.50
BC	0.7250	− 1.25	2.70
A²	4.57	2.51	6.63
B²	14.97	12.91	17.03
C²	9.24	7.18	11.30

### Optimization

As discussed earlier, the effects of Span 80, Tween 80, and stearic acid formulation variables on the responses of EE%, PS, and ZP were analyzed, reflecting their roles in drug entrapment and stability of the emulsion. An increasing proportion of stearic acid tended to raise EE% and PS due to the enhanced density and viscosity of the lipid matrix. Conversely, higher concentrations of Tween 80 reduced PS and ZP by increasing droplet stability and lowering interfacial tension, while Span 80 enhanced EE% by stabilizing the inner lipid phase and limiting drug diffusion into the aqueous phase. To determine the most balanced set of formulation parameters to achieve the desired nanoparticle characteristics, the following optimization process synthesized these relationships together statistically within the desirability function. Design Expert^®^ software was used to statistically optimize MOX-SLNs preparation by performing desirability function calculations and statistical analysis that assist in establishing the optimal combination of independent variables for achieving the desired outcomes. The desirability function, ranging from 0 to 1, indicates the extent to which the selected factor levels meet the objectives set, with values closer to 1 indicating an improved solution. In this work, restraints were applied to optimize ZP, PS, and encapsulation efficiency (EE%). Based on these parameters, the software recommended A F-opt with a total desirability value of 0.851. Table 13 presented the specific factor values corresponding to the F-opt. This formulation was further characterized under the same conditions to validate the efficiency of the optimization process.

**Table 13:** Predicted levels for F-opt and desirability.

Independent variable	Levels	Desirability
A-Stearic acid	4	0.851
B-Span 80	0.9	
C-Tween 80	0.3371	

####  Assessment of F-opt

##### PS, ZP and EE% results

For experimental validation of the Box–Behnken optimization model, a checkpoint batch of the optimized formulation (F-opt) was prepared. As previously indicated, EE%, PS, and ZP were measured, with values summarized in Table 14. The applicability of the optimization process in respect to accuracy and reliability was authenticated by the fact that experimentally obtained values for each response EE%, PS, and ZP fell within their confidence intervals.

**Table 14:** F-opt predicted and observed responses.

F-opt	Predicted mean	Observed	95% CI low for mean	95% CI high for mean
EE (%)	81.7055	79.563	74.4227	89.565
PS (nm)	290.126	257.7	177.677	810.001
ZP (mv)	− 55.7538	-52.4	− 55.7099	− 50.2881

##### Microscopical characteristics

**Fig. 12:**
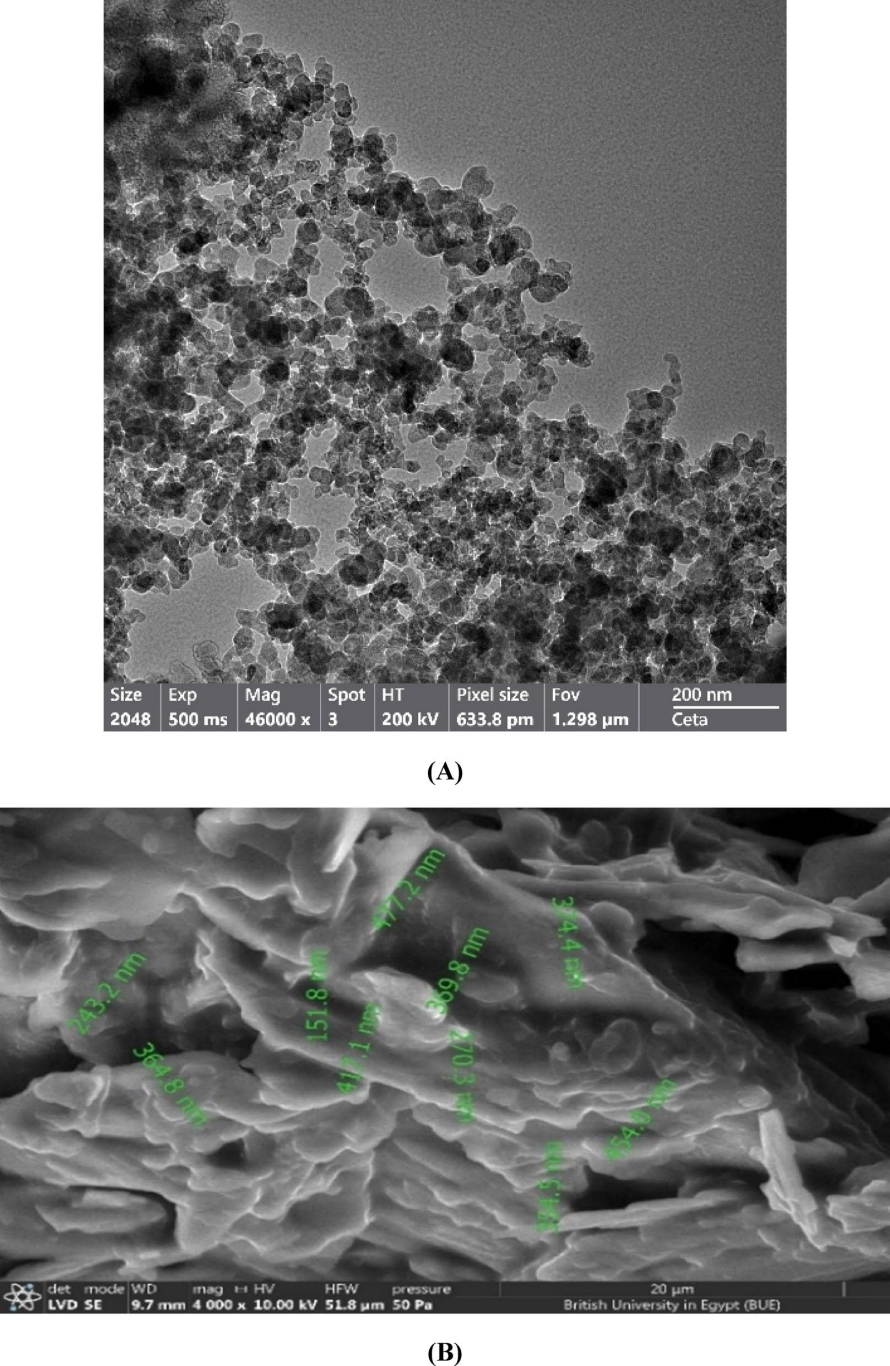
TEM image (**A**) and SEM image (**B**) of the lyophilized F-opt.

The surface morphology and structural characteristics of the F-opt were examined using both SEM and TEM. Regarding TEM images (Fig. 12A) clearly showed discrete, nearly spherical nanoparticles with smooth surface. In addition, they revealed a tightly packed cluster of smaller, shape-isomorphic particles that were closely packed in contact to yield a cohesive network. This clumping behavior was characteristic of surfactant-stabilized lipid nanoparticles and suggested excellent particle homogeneity and surface integrity. SEM images provided additional information on surface morphology (Fig. 12B). The particles with heterogeneous, irregular shapes were observed, potentially indicating minor aggregation or layering effects. In addition, the images showed an expanded field of view with multiple spherical particles dispersed across the surface. The irregular, non-smooth surface textures were evident and may have resulted from stearic acid crystallization during freeze-drying, a process that has been reported in SLNs preparations and may be accountable for surface roughness as well as for enhancing rearrangement of the lipid matrix^90^. Furthermore, an irregular surface morphology was observed to have large surface features presumably caused by crystallized lipid domains. Taken together, these SEM characteristics validated the formation of the SLNs and were consistent with earlier studies demonstrating that stearic acid-based SLNs exhibited rough or flaky surfaces following lyophilization, elucidating the role of lipid crystallization impacts on the morphological characteristics of the nanoparticles^24^.

#####  X-ray diffraction (XRD)

**Fig. 13:**
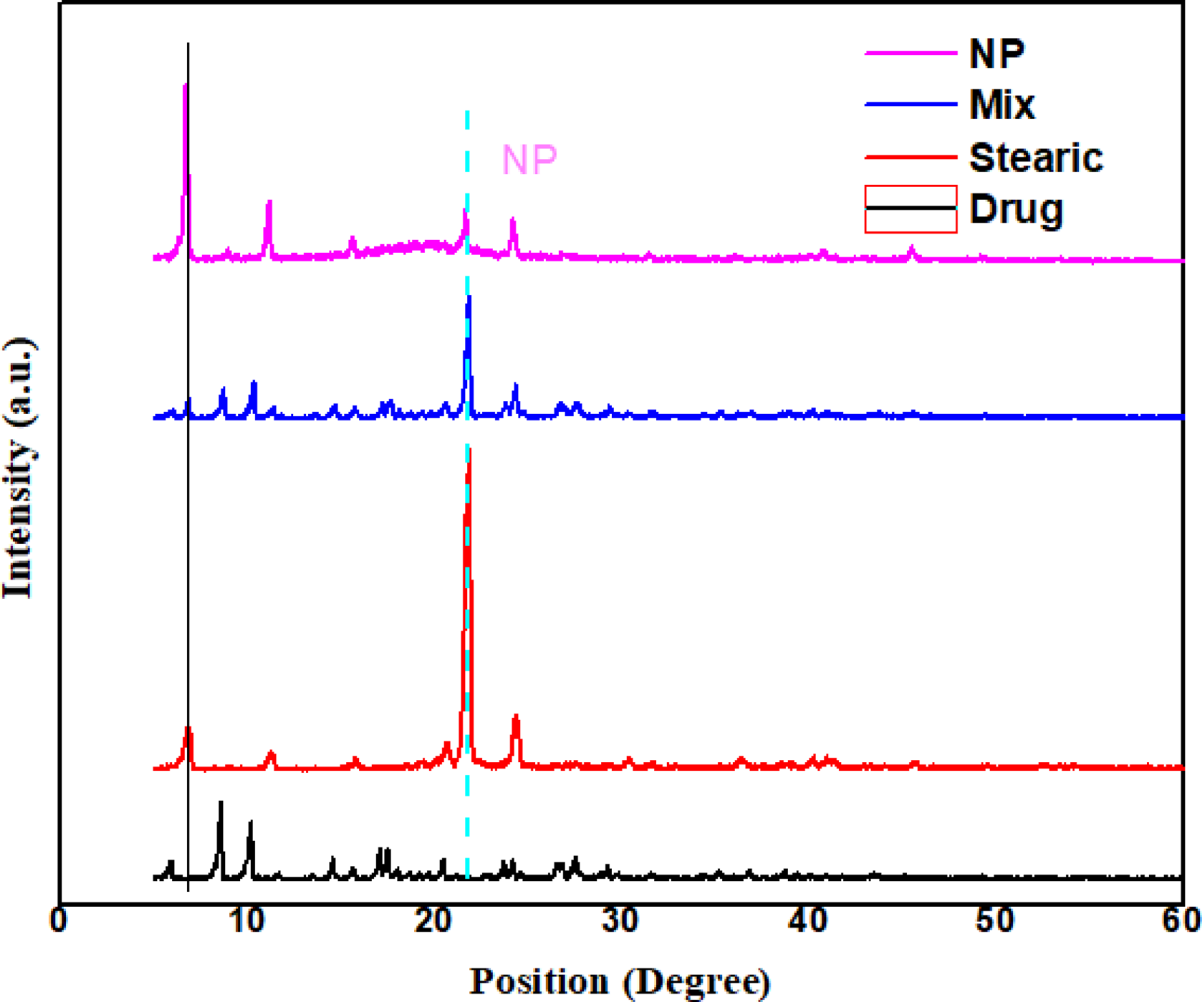
XRD stacked lines for pure drug, stearic acid, physical mixture of drug: stearic 1:1 and F-opt.

The crystallinity of the pure drug, stearic acid, physical mixture, and the prepared nanoparticles was investigated using XRD (Fig. 13). The pure MOX exhibited sharp diffraction peaks, confirming that it was a crystalline substance. Stearic acid also displayed characteristic peaks, confirming its crystalline state^91^. In the physical mixture, peaks attributable to both the drug and stearic acid were observed, indicating that simple mixture did not trigger significant physicochemical interactions. However, the XRD spectrum of the nanoparticles showed a decrease in the peak intensity of stearic acid, as well as the complete disappearance of some of the drug’s characteristic peaks. This implied that in the matrix of the nanoparticle, drug existed in the form of amorphous or molecularly dispersed drug with no crystalline structure.

#####  Fourier transform infrared (FTIR) spectroscopy

**Fig. 14:**
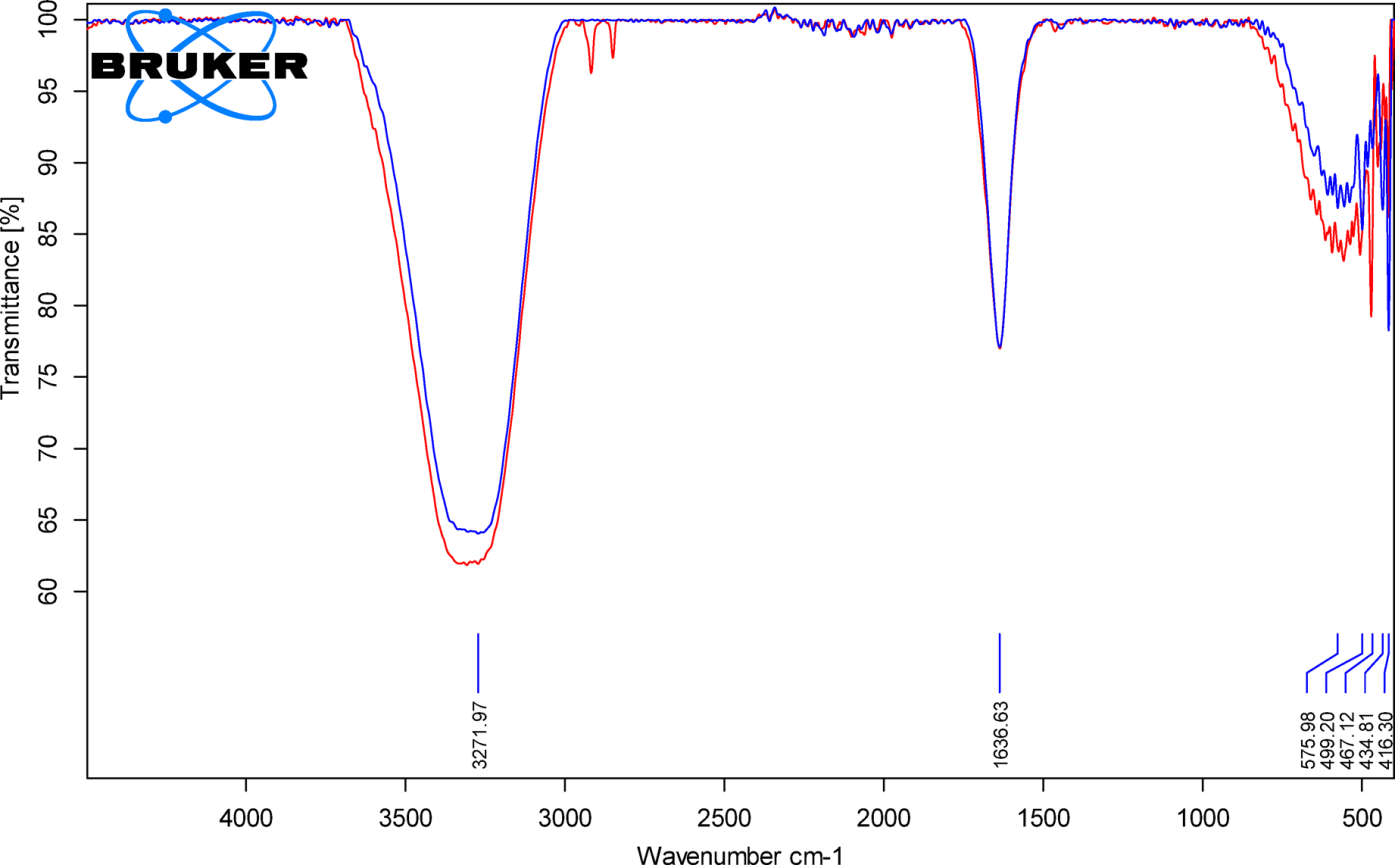
FTIR overlaid chart of pure drug (MOX, the blue line) and optimized SLNs formula (F-opt, the red line).

FTIR analysis was performed to identify any possible molecular interactions of MOX with lipid–surfactant matrix of the optimized SLN formulation (F-opt) containing MOX. As shown in Fig. 14, the pure MOX spectrum, characteristic peaks at 1620 cm⁻¹ and 1450 cm⁻¹ assigned to C = C and C–N stretching in the quinolone ring, broad band at 1715 cm⁻¹ due to carboxyl C = O stretch, and bands in the region 3450–3470 cm⁻¹ due to O–H and N–H stretching. The functionalities of fluoroquinolones, C–F and C–O–C stretching, were indicated by other bands at 1280 cm⁻¹ and 1040 cm⁻¹, respectively. In the F-opt spectrum these MOX-specific peaks remained with reduced intensity and slight broadening, but without any shift or disappearance, suggesting no new formation of functional groups. The chemical structure of the drug was unchanged after encapsulation, as attested by the retention of the major bands. The narrowing and loss of intensity would likely result from physical restriction of MOX in the lipid matrix and possible weak hydrogen bonding between MOX functional groups and polar stearic acid or surfactant termini, in contrast to chemical or electrostatic excipient interactions. This implies physical encapsulation or dispersion of MOX in the lipid matrix rather than covalent bonding. These findings are consistent with previous FTIR investigations of SLNs. For instance, formulations containing simvastatin^92^, dapagliflozin^93^, and coenzyme Q10^94^ formulations all exhibited characteristic drug-related peaks in their FTIR spectra without the appearance of new bands, indicating no chemical interaction between drug and lipid phases. The minor peak broadening or intensity loss was attributed to weak hydrogen bonding or physical entrapment in the lipid matrix. In concordance with our MOX-loaded SLNs results, these experiments confirm that entrapment of drug in SLNs involves mainly physical inclusion with the preservation of chemical integrity^95^.

##### Conclusion

Using a Box–Behnken design, the current study was successful in formulating and optimizing MOX-loaded solid lipid nanoparticles (MOX-SLNs) for identify the key formulation factors influencing zeta potential (ZP), particle size (PS), and entrapment efficiency (EE%). The optimized formulation (F-opt) yielded spherical, monodisperse nanoparticles, as evidenced by SEM and TEM. While FTIR and XRD analysis confirmed the preferred physicochemical characteristics, and revealed no chemical interactions between drug and excipients, substantiating physical encapsulation of the drug within the lipid matrix. These findings highlighted the potential of optimized MOX-SLNs that may be serve as a drug delivery system with high efficacy for treating chronic wounds. Therefore, our future prospectives based on the aforementioned data will aim to further investigate the optimized formula, that will involve several studies including in vitro tests such as drug release, kinetic modeling and antibacterial assessments. The upcoming study will also expand to involve ex vivo permeation, skin deposition, in addition to in vivo animal model.

## Supplementary Information

Below is the link to the electronic supplementary material.

Supplementary Material 1

Supplementary Material 2

Supplementary Material 3

Supplementary Material 4

Supplementary Material 5

Supplementary Material 6

## Data Availability

All data generated or analysed during this study are included in this published article and its supplementary information files.
