# Supplementary material for: Development and optimization of Moxifloxacin solid lipid nanoparticles via double emulsion organic solvent free technique applying Box–Behnken experimental design
Source: Sci Rep. 2025 Nov 26;15:42013. doi: 10.1038/s41598-025-26860-x (PMC12657925; doi:10.1038/s41598-025-26860-x)
Supplement: Supplementary file 1 — Supplementary Material 1 [file 41598_2025_26860_MOESM1_ESM.pdf]

Esraa Elshazly

Development and optimization of Moxifloxacin solid lipid nanoparticles for treating chronic wounds via double emulsion...

Quick Submit

Quick Submit

British University in Egypt

Document Details

Submission ID

trn:oid:::1:3294718899

Submission Date

Jul 13, 2025, 10:34 AM GMT+3

Download Date

Jul 13, 2025, 10:57 AM GMT+3

File Name

Manuscript_File_2.docx

File Size

3.1 MB

40 Pages

8,894 Words

47,955 Characters

*% detected as AI

AI detection includes the possibility of false positives. Although some text in this submission is likely AI generated, scores below the 20% threshold are not surfaced because they have a higher likelihood of false positives.

Caution: Review required.

It is essential to understand the limitations of AI detection before making decisions about a student’s work. We encourage you to learn more about Turnitin’s AI detection capabilities before using the tool.

Disclaimer
Our AI writing assessment is designed to help educators identify text that might be prepared by a generative AI tool. Our AI writing assessment may not always be accurate (it may misidentify writing that is likely AI generated as AI generated and AI paraphrased or likely AI generated and AI paraphrased writing as only AI generated) so it should not be used as the sole basis for adverse actions against a student. It takes further scrutiny and human judgment in conjunction with an organization's application of its specific academic policies to determine whether any academic misconduct has occurred.

Frequently Asked Questions

How should I interpret Turnitin's AI writing percentage and false positives?
The percentage shown in the AI writing report is the amount of qualifying text within the submission that Turnitin’s AI writing detection model determines was either likely AI-generated text from a large-language model or likely AI-generated text that was likely revised using an AI-paraphrase tool or word spinner.

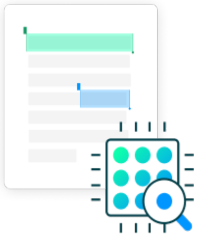

False positives (incorrectly flagging human-written text as AI-generated) are a possibility in AI models.

AI detection scores under 20%, which we do not surface in new reports, have a higher likelihood of false positives. To reduce the likelihood of misinterpretation, no score or highlights are attributed and are indicated with an asterisk in the report (*%).

The AI writing percentage should not be the sole basis to determine whether misconduct has occurred. The reviewer/instructor should use the percentage as a means to start a formative conversation with their student and/or use it to examine the submitted assignment in accordance with their school's policies.

What does 'qualifying text' mean?
Our model only processes qualifying text in the form of long-form writing. Long-form writing means individual sentences contained in paragraphs that make up a longer piece of written work, such as an essay, a dissertation, or an article, etc. Qualifying text that has been determined to be likely AI-generated will be highlighted in cyan in the submission, and likely AI-generated and then likely AI-paraphrased will be highlighted purple.

Non-qualifying text, such as bullet points, annotated bibliographies, etc., will not be processed and can create disparity between the submission highlights and the percentage shown.

Development and optimization of Moxifloxacin solid lipid nanoparticles for treating chronic wounds via double emulsion organic solvent free technique applying Box-Behnken experimental design

Esraa M.Elshazly[1,2], Mona G. Arafa[2,3,4*], Samia A. Nour[1]

[1]Department of Pharmaceutics and Industrial Pharmacy, Faculty of Pharmacy, Cairo University, Cairo, Egypt

[2]Department of Pharmaceutics and Pharmaceutical Technology, Faculty of Pharmacy, The British University in Egypt, Cairo, Egypt

[3]Chemotherapeutic Unit, Mansoura University Hospitals, Mansoura, Egypt

[4]Nanotechnology Research Center, The British University in Egypt, Cairo, Egypt

* Corresponding author: Mona G. Arafa (mona.arafa@bue.edu.eg)

Abstract

The current research proposes the preparation of Moxifloxacin-loaded solid lipid nanoparticles (SLNs) through a solvent-free double emulsion technique, to overcome chronic wound healing limitation. The independent variables: stearic acid, Span 80, and Tween 80 were optimized via Box–Behnken design, based on entrapment efficiency (EE%), zeta potential (ZP), and particle size (PS). Results indicated that increasing stearic acid enhanced EE%, Span 80 increase PS, and Tween 80 improved ZP. The Optimized SLNs formula had a ZP of −52.4 mV, EE% of ~79.6% and PS of 257 nm. Chemical compatibility was emphasized using FTIR, and XRD studies revealed reduced drug crystallinity within the lipid matrix, which confirmed successful encapsulation. Transmission electron microscopy (TEM) and scanning electron microscopy (SEM) imaging revealed nanoparticles with defined spherical shape and smooth surface. The optimized formula is anticipated to enhance dermal distribution and prolonged antibiotic release, thereby offering an efficient therapeutic strategy for the management of chronic wounds.

Keywords

Chronic wounds, Moxifloxacin, Nanotechnology, Solid lipid nanoparticles, Double emulsion technique, Box-Behnken design, Optimization

Introduction

The process of wound healing in healthy people is highly precise and integrated. Any interruption in this precise and methodical process can result in the formation of chronic wounds [1]. Diabetes mellitus (DM) is a principal factor that hinders wound healing, recognized as the most common chronic disease[2,3]. Approximately 15% of diabetic people develop foot ulcers, which are particularly prone to infections. Infected wounds considerably hinder normal healing. The International Working Group on Diabetic Foot (IWGDF) 2019 guidelines [4] recommended the empirical administration of broad-spectrum antibiotics such as penicillins, fluoroquinolones, and cephalosporins, either orally or intravenously, to prevent additional problems. Moxifloxacin (MOX), a broad-spectrum hydrophilic antibiotic [5], is recognized for its strong antibacterial efficacy and effectiveness in treating infections. Nonetheless, the transdermal administration of hydrophilic pharmaceuticals presents considerable obstacles owing to their limited permeability across the lipid-dense stratum corneum. Nanotechnology has arisen as an approach to address drawbacks of traditional dosage forms by enhancing medicinal efficacy. Nanoparticles provide multiple methods to improve wound healing, as various studies have shown their capacity to accelerate the healing [6–8]. The integration of hydrophilic pharmaceuticals such as MOX into solid lipid nanoparticles (SLNs) has demonstrated an enhancement in skin penetration and an improvement in drug delivery. SLNs enhance drug stability and bioavailability while facilitating regulated and sustained release, which is especially advantageous for wound healing. Despite these advantages, encapsulating hydrophilic medicines into SLNs remains challenging due to the inadequate interaction between hydrophilic and lipophilic components, which often leads to phase separation. This separation compromises drug delivery efficiency and results in poor release profiles. Despite these challenges, the double emulsion method is the optimal choice for hydrophilic drugs, as it prevents drug dispersion and leakage into the external aqueous layer of the emulsion[9]. Studies suggested that the double-emulsion technique is an effective approach to address this issue, facilitating better incorporation efficiency and enhanced performance[10], as the drug is confined within the inner aqueous core. The multilayer structure minimizes drug leakage, enhances stability, and improves both the shelf life and effectiveness of SLNs. Moreover, using this technique without using any organic solvent further increases the advantages of the method, making it safer and more environmentally friendly [11]. The Box-Behnken design (BBD) is utilized as a statistical tool to evaluate the impact of various independent factors on the targeted responses, hence optimizing the preparation process. Compared to full factorial design, BBD facilitates an effective optimization process with fewer trials. This is especially beneficial for examining the relationships between independent variables and their effects on outcomes such as encapsulation efficiency EE%, PS, and ZP [12]. This research aims to develop and evaluate SLNs containing MOX through the double-emulsion method to boost skin absorption, promote drug stability, and accelerated wound healing. The study utilizes thorough analysis through the BBD, examining the impact of several independent parameters: amount of used solid lipid and amounts of utilized surfactants on EE%, PS, and ZP. The study further examines the interaction among individual variables by infrared (IR)

spectroscopy and X-ray diffraction (XRD) to verify compatibility and evaluate crystalline characteristics. SEM and TEM were employed for microscopic examinations to check the morphology of the optimized SLNs.

Materials

Moxifloxacin was generously provided by Eva Pharmaceutics, Cairo, Egypt. Stearic acid (reagent grade, 95%) and Poloxamer 80 were obtained from Sigma-Aldrich, St. Louis, MO, USA. Tween 80 (Polysorbate 80) was purchased from Fisher Scientific, Loughborough, UK. Span 80 (Sorbitan monooleate) was purchased from Sigma-Aldrich, USA. L-α-Lecithin, granular (from soybean oil) was purchased from ACROS Organics (Geel, Belgium), and lecithin (90%, from soybean, solid) was obtained from Alfa Aesar (Thermo Fisher Scientific, Germany). All chemicals and reagents used were of analytical reagent grade to ensure reliable results.

Methods

1.Ultraviolet (UV) spectrophotometry evaluation of MOX

MOX solution was prepared in 100 ml volumetric flask of 100 µg MOX in 100 ml distilled water. Then the solution was scanned using a spectrophotometer to determine the maximum wavelength of absorbance (λ_{max}) in the ultraviolet range of 200 to 400 nm using the UV/ Visible Spectrophotometer (Jasco – V-630- Japan)

2.Calibartion curve construction

A stock solution of 100 µg/ml MOX in distilled water was prepared in a 100 ml volumetric flask. Serial dilutions of the stock solution were made in 10 ml volumetric flasks to achieve concentrations of 1, 2, 3, 4, 5, 6, 7, 8, 9, 10, 11, 12 and 13 µg/ml using the UV/ Visible Spectrophotometer the absorption of the solutions was determined at the previously determined (λ_{max})[13]. Mean absorbance of three replicated readings was plotted against corresponding concentrations on the Y and X-axes, respectively, to obtain a calibration curve. Linear regression analysis was performed using Excel Microsoft® software to display the equation of the best-fit line. The procedural constant (K) was then determined as the inverse of the slope obtained from the equation.

3.Preparation of MOX SLNs

3.1. Selection of independent variables

Critical independent parameters in this method include the type and quantity of lipid (single or multiple), type and concentration of surfactants, the addition of cosurfactants, processing temperature, the ratio of double emulsion to cold water, stirring time and amplitude[14]. Optimizing these parameters helps overcome the major drawbacks of this technique, such as instability due to coalescence of aqueous droplets within the oily phase, aggregation tendencies, and larger particle sizes[15]. Prior to designing complete experimental study, an initial screening was conducted to

inform the choice of preparation components. evaluating different reagents for the preparation of SLNs containing a hydrophilic drug (MOX). The SLNs were prepared using the double emulsion method (w/o/w), which requires the use of solid lipid and two surfactants: one in the primary (w/o) emulsion phase and another in the secondary (w/o/w) phase, to ensure emulsion stability. In the primary phase, the surfactant was mixed with the melted lipid and should possess a low hydrophilic-lipophilic balance (HLB) value, indicating lipophilic properties[16] .For the primary emulsion, two different lecithins with varying molecular weights were evaluated. L-α-Lecithin granular and lecithin 90% solid), Subsequently, Span 80 was evaluated. For the secondary phase, Poloxamer 80 and Tween 80 were tested at low concentrations. The selection of such excipients was based on literature [14,17–20] and preliminary laboratory experiments.

3.2. Experimental design and statistical analysis

A three-factor, three-level (3^3) BBD was applied to statistically optimize the preparation of MOX SLNs. Fifteen trials of SLNs were conducted, involving three center points, using Design-Expert software (version 13.0.5.0). The BBD was used to study the response surface methodology involving three independent variables: the amount of lipid stearic acid, the amount of the first surfactant (Span 80, B), and the percent of the second surfactant solution (Tween 80, C). The evaluation of each variable was carried out at three levels (-1,0,1) the actual values of these levels were shown in Table 2. These ranges were chosen according to pervious literatures with some modification [17,21,22] as shown in Table 1 . The three dependent variables encompass EE%, PS, and ZP, as detailed in Table 1. The experimental design was evaluated using ANOVA, with p-values below 0.05 and F-values greater than 0.05 consider significant[23]. Preparation responses were analyzed via three mathematical polynomial models: linear, two-factor interaction (2FI), and quadratic models. The optimal model was identified through ANOVA metrics, particularly R^2 values (predicted and adjusted) and adequate precision (>4). A close correspondence between predicted and adjusted R^2 (difference < 0.2) validated the model, while a coefficient of variation (CV% < 10%) confirmed reproducibility [24]

Table 1: independent and dependent variables for the BBD methodology utilized for the preparation of MOX SLNs

**Independent variable**	**Levels**			**Dependent variable**
	**Low (-1)**	**Medium (0)**	**High (1)**	
**A: Stearic acid (grams)**	**0.9**	**2.25**	**4**	**Y1 : EE%**
**B: Span 80 (grams)**	**0.9**	**2.25**	**4**	**Y2 : PS**
**C: Tween 80 (grams)**	**0.1**	**0.25**	**0.4**	**Y3 : ZP**

Table 2: Composition of SLNs developed using 3^3 Box–Behnken Design (BBD)

Variables in their actual values			
Trials	A: Stearic acid	B: Span 80	C: Tween 80
	Grams	Grams	Grams
T1	2.45	2.45	0.25
T2	2.45	0.9	0.1
T3	4	2.45	0.4
T4	2.45	4	0.1
T5	2.45	2.45	0.25
T6	4	4	0.25
T7	0.9	0.9	0.25
T8	2.45	2.45	0.25
T9	0.9	2.45	0.4
T10	4	2.45	0.1
T11	2.45	0.9	0.4
T12	4	0.9	0.25
T13	0.9	2.45	0.1
T14	0.9	4	0.25
T15	2.45	4	0.4

3.3. Development of MOX-SLNs through double emulsion method

Double emulsion organic solvent-free technique was applied to prepare the fifteen trials with the following detailed steps. Initially the first emulsion (w/o) was prepared by melting the calculated amount of solid lipid (stearic acid) above its melting point at 70°c and mixed with the determined amount of Span 80 [25]. Then the aqueous phase containing the drug was added dropwise to this mixture under probe sonicator (Sonics Vibra Cell power 130Watt, frequency 20kHz, made by Sonics & Materials Inc, Newtown, CT) with amplitude 75% for 30 seconds. The amount of drug was fixed across all trials, and a primary w/o emulsion was formed. The secondary emulsion was then prepared through adding a predetermined amount of Tween 80 ,in form of aqueous solution, [26] dropwise under stirring at 1000 rpm for 30 min using hot plate magnetic stirrer (MSH-20D, DAIHAN Scientific, Korea). Finally, this formed w/o/w emulsion was poured into cold water (2-5 °C) with a ratio of 1:20 under stirring then this mixture was homogenized for 3 min at 1000 rpm using homogenizer (WiseTis HG-15D, Daihan Scientific, Korea) [27]. The formed SLNs went through centrifugation in a cooling centrifuge (Centurion Ltd., PRO-Research K241R, United Kingdom) at 15,000 rpm and 4°C for a duration of 2 hours. The supernatant was collected to further assess drug entrapment efficiency. Figure 1 illustrates the schematic representation of the method mentioned above.

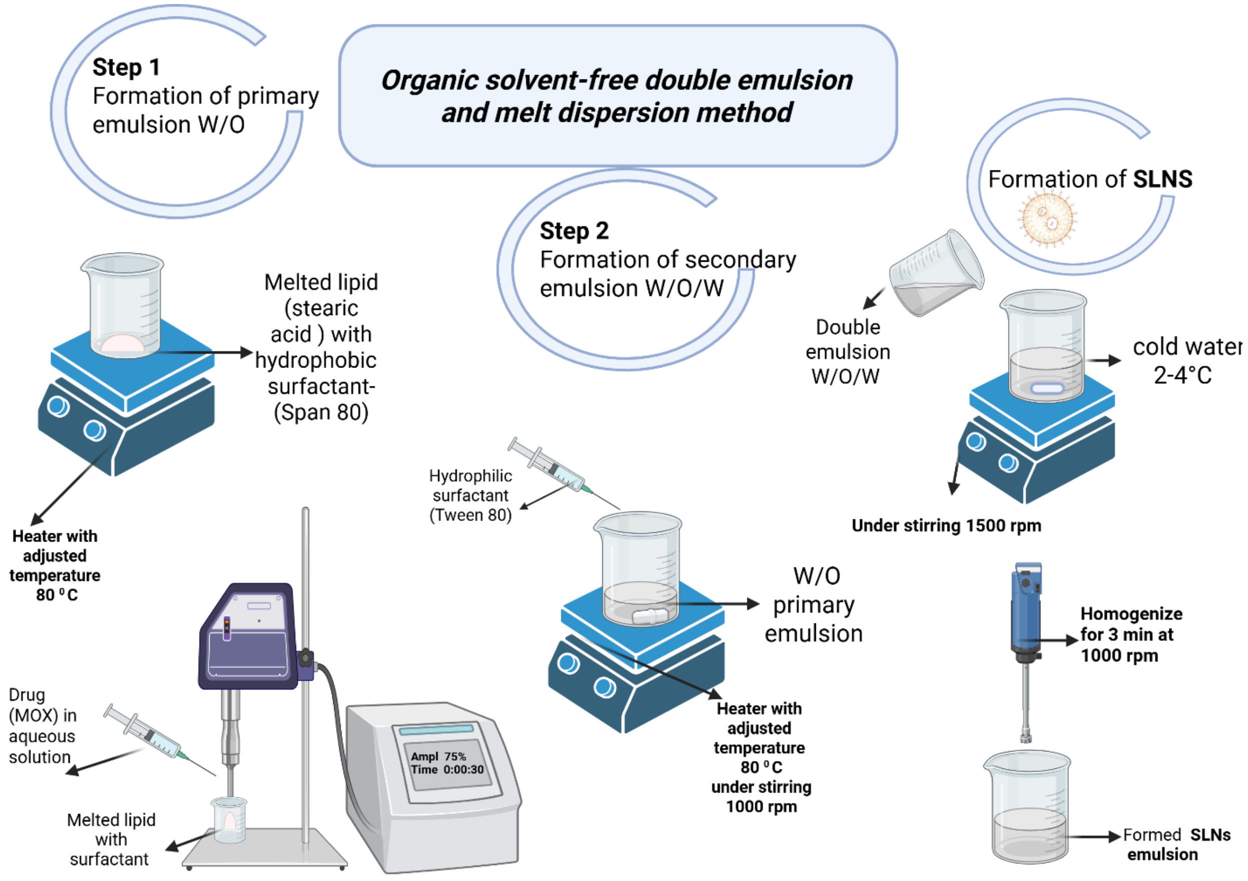

Figure 1: Schematic diagram illustrating the double emulsion organic solvent-free method for SLNs preparation

4.Characterization of formed MOX-SLNs

4.1. Entrapment Efficiency (EE%)

An indirect method was used to determine the amount of unentrapped drug in the supernatant after centrifugation using cooling centrifuge (Centurion Ltd., PRO-Research K241R, United Kingdom). The absorbance of the supernatant was measured at predetermined MOX λ_{max} (293 nm), and the resulted absorbance was substituted into the calibration equation (y = 0.0985x - 0.0507) to calculate the drug concentration. The exact conditions were applied to develop blank nanoparticles, and their supernatant layer was used as control. The EE % was then calculated using the following equation[28].

$$EE\ \% = \frac{\text{Total amount of drug} - \text{unentrapped}}{\text{Total amount of drug}} \times 100 \qquad \text{(Equation 1)}$$

4.2. Analysis of particle size and zeta potential

Zetasizer (Nano ZS, Malvern, UK) was used to measure the average PS &ZP. These were essential features to detect the physical stability of the system [29]. All measurements were performed at room temperature (25 °C) and sufficient dilution was done using water for all dispersions [30]. Size measurements were conducted using Dynamic Light Scattering (DLS) technique, which involves assessing Brownian motion and correlating it to the dimensions of the particles. The ZP was determined by measuring Electrophoretic Mobility, which means the observed rate of migration of a component divided by the electric field strength in the specified medium.

5.Optimization

All outcomes were optimized using Design Expert® software (version 13) by applying certain constraints on the selected dependent variables. The optimization is based on maximizing ZP, EE% and minimizing PS. For predictive validation of the derived model, the optimized formula (F-opt) was again prepared then Freeze-dried in a lyophilizer (Alpha 1-2 LDplus, CHRIST, Germany) with the condenser maintained at -45 °C for 24 hours to be analyzed for the above-mentioned parameters.

5.1. Assessment of F-opt

5.1.1. Entrapment Efficiency %

The encapsulation efficiency was calculated for the prepared F-opt using the previously stated indirect method. Cold centrifugation was performed to separate the supernatant which was analyzed through UV spectrophotometer at predetermined (λ_{max}).

5.1.2. Particle size and zeta potential

The PS &ZP of the F-opt were determined using Malvern zeta sizer as stated above in details in section (4.2.)

5.1.3. Microscopical examination

TEM and SEM were used to examine morphology, nanoparticles preparation and surface characteristics of the F-opt. SEM imaging was conducted using a ThermoFisher Quattro S Field Emission Gun SEM (USA), and \the inner structure examination and high-resolution imaging were conducted on a Thermo Scientific™ Talos™ F200i S/TEM (20–200 kV), a field emission SEM.

5.1.4. X-ray diffraction (XRD)

The crystalline or amorphous nature and phase composition of the pure MOX and Stearic acid, physical mixture (MOX:Stearic acid,1:1 W/W) and lyophilized F-opt, was analyzed using X- ray diffractometer (Panalytical Empyrean 3 diffractometer (Malvern, Netherlands)) equipped with a copper Kα radiation source (λ = 1.5406 Å). The scanning was conducted over the period of 37 minutes, over the range of 2θ = 4° to 90°, using a step size of 0.02° and a measurement time of 0.5

seconds per step. These conditions gave adequate resolution to resolve characteristic diffraction peaks as well as to determine variations in crystallinity among samples.

5.1.5. Fourier transform infrared (FTIR) spectroscopy

FTIR was performed to verify if the chemical structure of MOX was preserved after encapsulation in lyophilized optimum SLNs and to evaluate potential drug-excipient interactions. The spectral analysis was conducted on a RAM II FT-Raman module coupled to a Vertex FTIR spectrometer (Bruker, Germany) in the wavelength range 500–4000 cm^{-1} with high spectral resolution up to 0.1 cm^{-1}. The studies were conducted using an attenuated total reflectance (ATR) accessory, and spectra were measured with 16 cm^{-1} resolution and a minimum scan rate of 50 scans per second.

Results and discussion

1.Ultraviolet (UV) spectrophotometry of MOX

As shown in Figure 2, UV absorption spectra of MOX dissolved in distilled water exhibits a maximum absorbance (λ_{max}) at 293 nm, this value was consistent with other published literature [31]

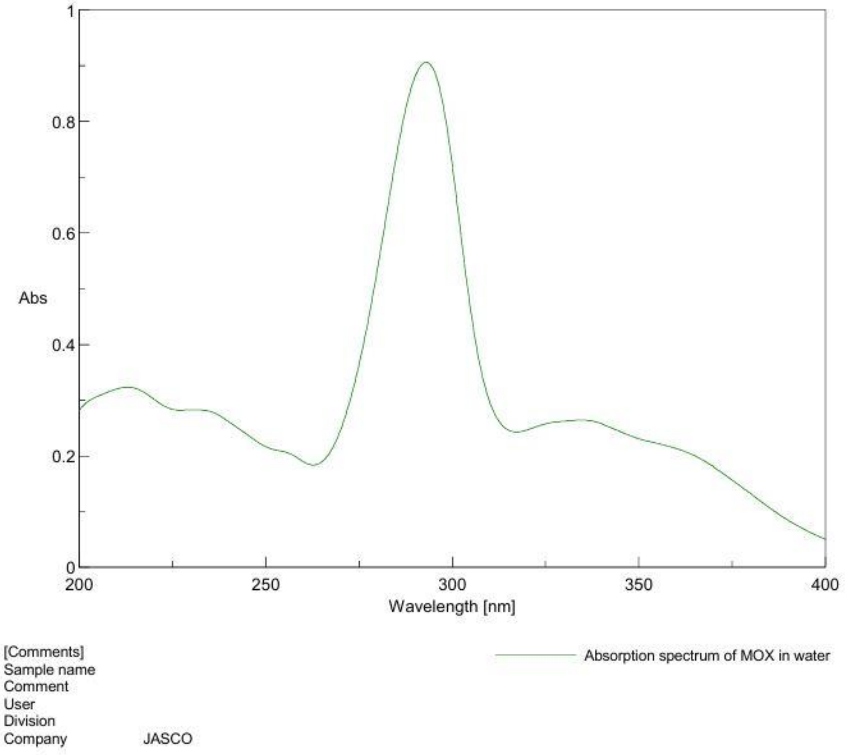

Figure 2: Absorption spectrum of MOX in distilled water

2.Calibartion curve

The UV absorbance of MOX at the predetermined λ_{max} of 293 nm was measured using solutions of serial dilutions prepared in distilled water. The mean values of three independent measurements were calculated and used to construct the calibration curve presented in Figure 3. The regression equation and the coefficient of determination (R^2) (0.9973) are displayed on the graph of Fig.3, this indicating good linearity of the MOX concentration absorbance in accordance with Beer_ Lambert's law. The procedural constant (K) was determined and was found to be 10.

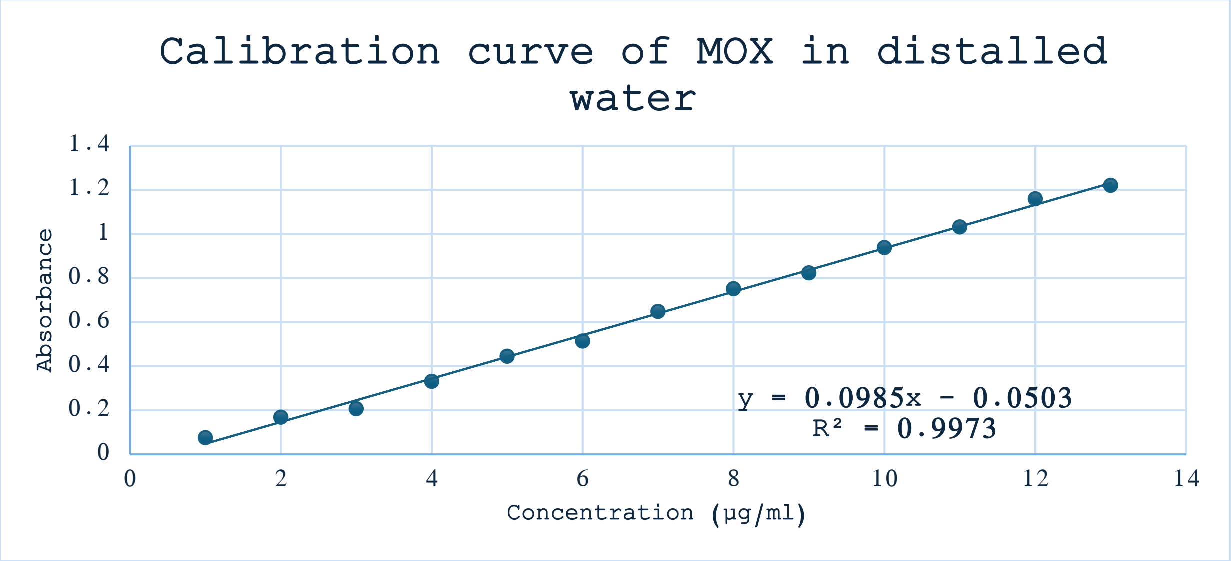

Figure 3: MOX calibration curve in distilled water at λ_{max} 293 nm

3.Selection of independent variables

In preparation of all trials, a few parameters were kept constant to ensure reproducible and consistent results. They were MOX amount, sonication time and amplitude of probe (75% for 30 seconds), temperature (70°C), agitation time for secondary emulsification (30 minutes), stirring speed (1000 rpm), prepared SLNs: cold water ratio (1:20), homogenization speed and time (3 minutes at 1000 rpm). As shown in Table 1, the only independent variables that were investigated systematically were solid lipid content and surfactant amounts and type. Stearic acid was chosen as the type of solid lipid for preparation of nanoparticles due to its good biocompatibility and low toxicity in body and it is commonly chosen in pharmaceutical use as lipid matrix to prepare solid nanoparticles [32]. In the preliminary study the aforementioned two forms of lecithins formed clumps that failed to melt even when the temperature was raised above the lipid's melting point, leading to unsuccessful emulsion formation. Subsequently, Span 80 was evaluated and resulted in no phase separation, forming a more stable emulsion. This confirmed that non-ionic surfactants were preferable for SLN preparation [33].For the secondary phase, Poloxamer 80 and Tween 80 were tested at low concentrations, both led to less stable emulsions with phase separation. Poloxamer 80, even at higher concentrations, continued to result in phase-separated emulsions, while Tween

80 produced a homogenous and stable emulsion at higher concentrations. Consequently, a combination of Span 80 and Tween 80 was deemed optimal due to their structural compatibility [33]

4.Statistical evaluation of BBD

Regression analysis was employed to model the responses into quadratic, two-factor interaction (2FI), and linear models. The responses were EE% (Y_1), PS (Y_2), and ZP (Y_3). The optimal model was selected based on the highest lack of fit value and adjusted and predicted coefficient of determination R^2 values from the respective models. In addition, analysis of variance (ANOVA) was employed to determine the statistically significant factors that influence each response.

4.1. Impact of independent factors on EE% (Response 1-Y1)

The EE% of the developed SLNs ranged from 62.57 ± 2.91 in T14 to 91.96 ± 5.27 in T4, as shown in Table 3. The relatively high EE%, exceeded 90%, may be due to the entrapment of hydrophilic drug (MOX) in SLNs with stearic acid as the lipid matrix via the double emulsion method. This might be attributed to the ability of stearic acid to create a stable lipid matrix that favors encapsulation of hydrophilic drugs. Similar results have been reported in a study using zidovudine as hydrophilic drug, where stearic acid-coated SLNs prepared by the double emulsion solvent evaporation process gave high EE values[34]. Another reason was that using stearic acid led to the formation of an imperfect crystalline matrix with enhanced drug encapsulation capability. This was supported by the findings of Subroto et al[35], who used the double emulsion technique to encapsulate ferrous sulfate, a water- soluble drug, by using a combination of stearic acid and high-monolaurin fat. This approach led to extremely high levels of EE ranging from 99.97% to 99.99%.

Table 3: Mean values of EE% for all trials

	Factor 1	Factor 2	Factor 3	Y 1
Trials	**A:Stearic acid**	**B:Span 80**	**C:Tween 80**	**Mean EE ± SD**
	**Grams**	**Grams**	**Grams**	**%**
T1	2.45	2.45	0.25	**88.10 ± 3.78**
T2	2.45	0.9	0.1	**80.14 ± 9.20**
T3	4	2.45	0.4	**90.35 ± 9.36**
T4	2.45	4	0.1	**91.96 ± 5.27**
T5	2.45	2.45	0.25	**88.36 ± 10.83**
T6	4	4	0.25	**89.70 ± 6.12**
T7	0.9	0.9	0.25	**70.52 ± 13.94**
T8	2.45	2.45	0.25	**75.19 ± 7.17**
T9	0.9	2.45	0.4	**71.11 ± 5.72**
T10	4	2.45	0.1	**87.31 ± 4.53**
T11	2.45	0.9	0.4	**70.83 ± 11.62**
T12	4	0.9	0.25	**74.04 ± 10.30**

T13	0.9	2.45	0.1	**79.08 ± 10.12**
T14	0.9	4	0.25	**62.57 ± 2.91**
T15	2.45	4	0.4	**81.11 ± 6.70**

Linear model was the most significant unaliased model for the analysis of entrapment efficiency, as indicated from Table 4, having a p-value of 0.0337 and an R^2 value of 0.5319. The lack of fit of the model was insignificant compared to the pure error, as indicated by the p-value of 0.6544, which shows a good fit. A significant linear relationship among the independent and dependent variables was clearly indicated by the lack of fit, which was not significant ($p > 0.05$). Linear models' relatively higher values of the predicted and adjusted R^2 confirm the model to be appropriate for the data 36. A final model equation for EE% was as follows:

EE%= +80.02+7.27A +3.73 B -3.14C (Equation 2)

Table 4: Proposed model for observed EE%

Source	F-value	P-value	
Linear vs Mean	**4.17**	**0.0337**	**Suggested**
2FI vs Linear	**1.23**	**0.3595**	
Quadratic vs 2FI	**1.96**	**0.2389**	
Cubic vs Quadratic	**0.3302**	**0.8093**	**Aliased**

Table 5 shows the result of ANOVA for the final model. As indicated, the model was statistically significant as evidenced by the fact that the p-value was below 0.05. Besides, the data show that the drug EE% was statistically affected by factors: (A) the amount of stearic acid content as the p-value for factor A was < 0.05, indicating it to be statistically significant. Further, the high F-value was an indicator of the existence of significant mean differences, confirming its role in contributing to the model.

Table 5: ANOVA for Linear model (Response 1: Entrapment Efficiency)

Source	F-value	p-value	
Model	4.17	0.0337	Significant
A-Stearic acid	8.62	0.0135	
B-Span 80	2.27	0.1601	

C-Tween 80	1.61	0.2312	

An increase in the amount of stearic acid was correlated to higher EE%, as the positive coefficient value reported in Table 6, which suggests direct linear correlation to EE%. The one-factor plot presented in Figure 4 (A) also confirmed that. The coefficient estimate represents the way in which the response variable was expected to change for every unit increase in the predictor variable, while all other variables were kept constant. In addition, T14, which was loaded with the minimum amount of stearic acid (0.9 g), had the minimum value for EE% (62.57 ± 2.91), and this affirms the reported correlation. The observations were in agreement with those by Silpa et al [36] , who correlated higher value for EE% to higher content in lipids, which hindered the partitioning of MOX into external aqueous phase on emulsification. Through the process, there was the formation of drug-enriched core in the SLNs, thereby improved drug encapsulation. Similarly, Darsh et al [37] , reported direct proportionality to lipid content and EE, where higher lipid content was reported to enhance the matrix capacity for drug loading. Moreover, the illustrated 3D surface plot in Figure 5 and the contour plot in Figure 6 confirm the significant effect of Stearic Acid on EE%. The upward sloping surface towards the rear-right corner in 3D plot which relates to increasing the amount of stearic acid resulting in higher EE%. The same was supported by the gradual color graduation in the Fig 6, which shows equal and complementary improvement in EE with higher concentrations in stearic acid amount [38]. The positive estimate of the coefficient in Table 6 suggests a direct influence of Span 80 concentration on EE%, however, relatively small and not significant with p-value (0.1601). as can be seen in Figure 4 (B). Although Tween 80 did not have a statistically significant effect on EE% ($p = 0.2312$), the negative coefficient in Table 6 suggests a potential negative trend between Tween 80 amount and EE% as indicated in Figure 4 (C).

Table 6: Coefficients in terms of coded factors regarding EE%

Factor	Coefficient Estimate	95% CI Low	95% CI High
Intercept	80.02	76.05	84.00
A-Stearic acid	7.27	1.82	12.71
B-Span 80	3.73	-1.72	9.17
C-Tween 80	-3.14	-8.58	2.31

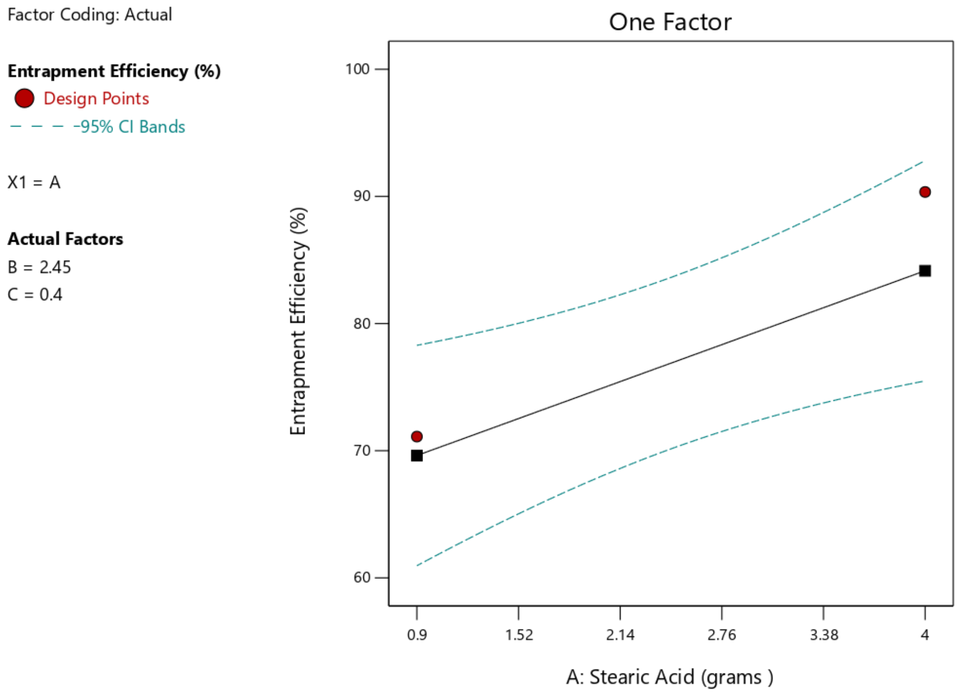
Factor Coding: Actual
Entrapment Efficiency (%)
Design Points
95% CI Bands
X1 = A
Actual Factors
B = 2.45
C = 0.4
One Factor
Entrapment Efficiency (%)
100
90
80
70
60
0.9
1.52
2.14
2.76
3.38
4
A: Stearic Acid (grams)

(A)

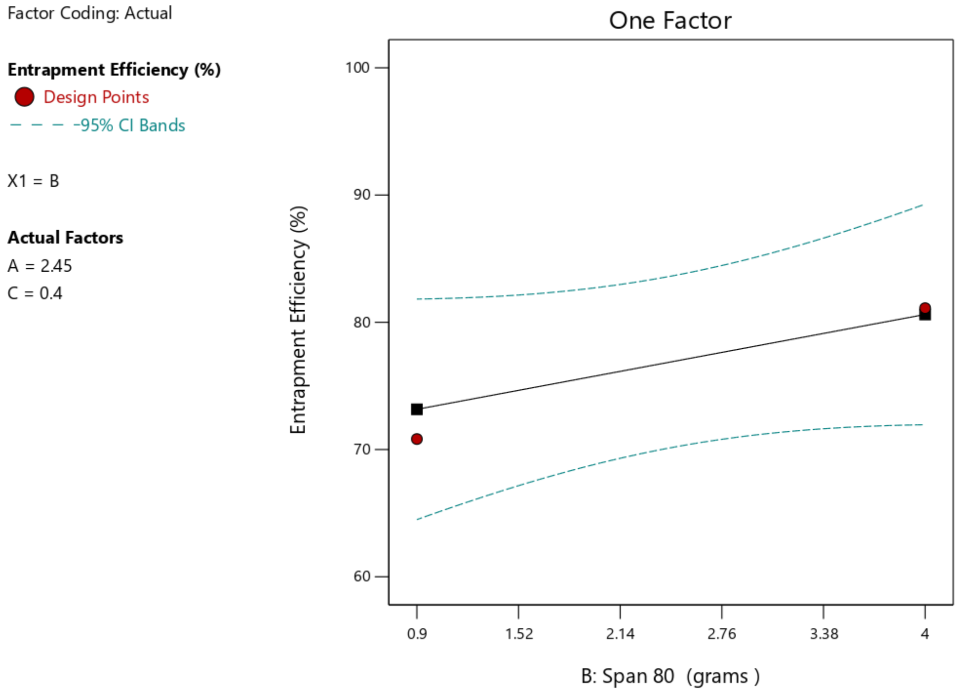
Factor Coding: Actual
Entrapment Efficiency (%)
Design Points
95% CI Bands
X1 = B
Actual Factors
A = 2.45
C = 0.4
One Factor
Entrapment Efficiency (%)
100
90
80
70
60
0.9
1.52
2.14
2.76
3.38
4
B: Span 80 (grams)

(B)

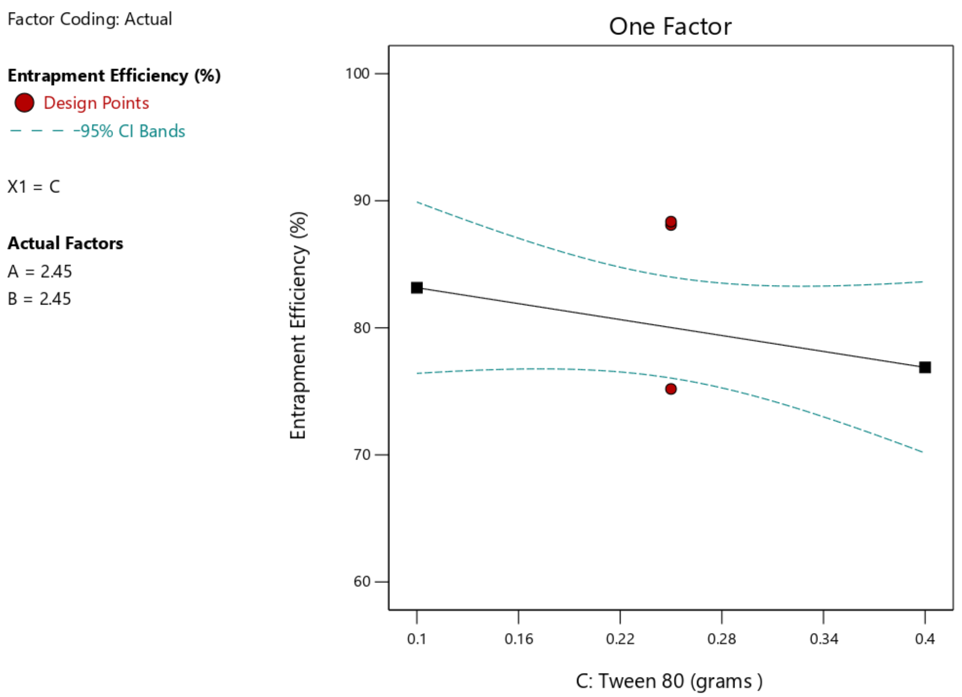

(C)

Figure 4: Line plot for the effect of Stearic acid amount (A), Span 80 amount (B), and Tween 80 amount (C), on the EE%.

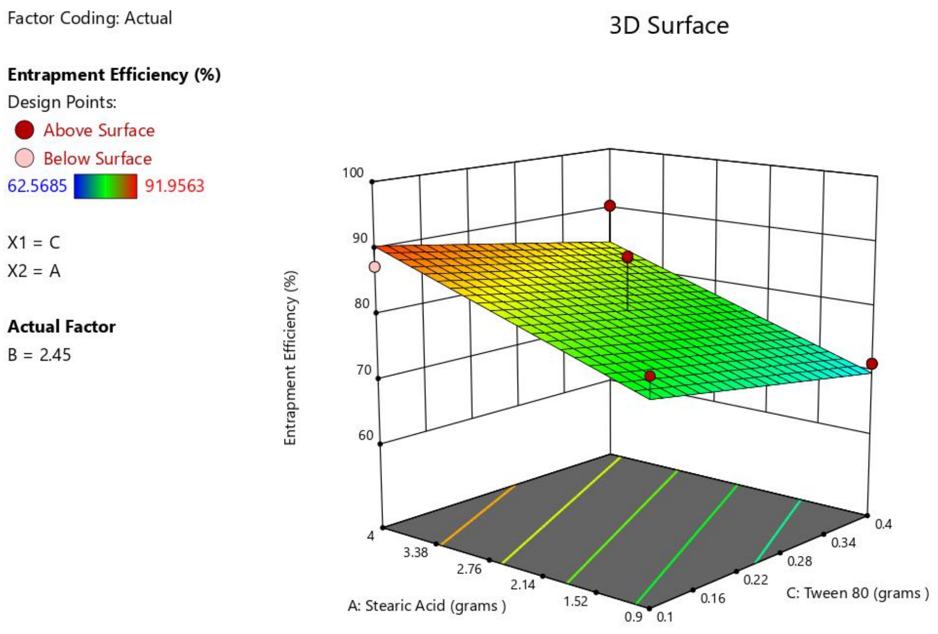

Figure 5: 3D surface plot of main effect of Stearic acid amount (A) and Tween 80 amount on EE%

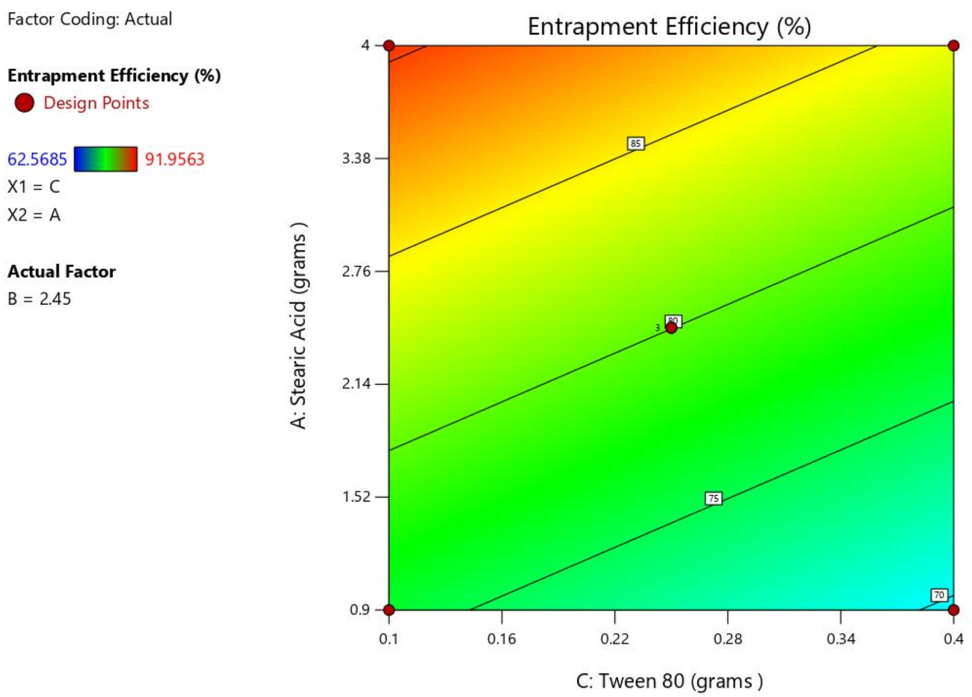

Figure 6: Contour plot of main effect of stearic acid amount and Tween 80 amount on EE%

4.2. Impact of independent factors on PS (Response 2-Y2)

The efficiency of dermal drug delivery and tissue penetration was greatly influenced by the size of nanoparticles. Recent studies have shown that nanoparticles of a size of approximately 80–100 nm can penetrate deeper into the skin, whereas particles of approximately 500–600 nm in diameter can provide better skin deposition [39]. These findings suggest that the optimal PS would most likely be determined by the compromise between drug residence in the skin layers and the penetration depth. Whereas nanocrystals in the size range 100-700 nm have historically been associated with enhanced delivery across the skin [40], PS may require optimization in this case to affect these specific therapeutic outcomes.[41,42]. Thus, the main target was to minimize the PS, as shown in Table 7 PS ranged from 182.6 ± 6.5 in T2 to 1425.0 ± 55.0 in T6.

Table 7: Mean values of PS for all trials

	**Factor 1**	**Factor 2**	**Factor 3**	**Response 2**
**Trials**	**A:Stearic Acid**	**B:Span 80**	**C:Tween 80**	**PS**
	Grams	Grams	Grams	nm
T1	2.45	2.45	0.25	640.0 ± 20.0
T2	2.45	0.9	0.1	182.6 ± 6.5
T3	4	2.45	0.4	355.6 ± 15.5
T4	2.45	4	0.1	1020.0 ± 40.0
T5	2.45	2.45	0.25	917.9 ± 42.6
T6	4	4	0.25	1425.0 ± 55.0
T7	0.9	0.9	0.25	290.0 ± 15.0

T8	2.45	2.45	0.25	642.2 ± 22.7
T9	0.9	2.45	0.4	694.9 ± 25.0
T10	4	2.45	0.1	742.3 ± 32.5
T11	2.45	0.9	0.4	360.0 ± 20.0
T12	4	0.9	0.25	420.2 ± 15.0
T13	0.9	2.45	0.1	230.1 ± 15.0
T14	0.9	4	0.25	415.4 ± 15.0
T15	2.45	4	0.4	210.0 ± 10.0

The significant model for the analysis of PS was 2FI-two factor interaction, according to the data in Table 8. The p-value was 0.0589 and an R^2 value of 0.7467. The lack of fit of the model was insignificant compared to the pure error, as indicated by the p-value of 0.3141. A significant relationship among the independent and dependent variables was clearly indicated by the lack of fit, which was not significant ($p > 0.05$). The model represented equation is:

PS =+569.86+ 163.975 A+ 227.2125 B- 69.2625 C+ 219.575 AB- 212.625 AC-246.8BC (Equation 3)

Table 8: Proposed model for observed PS

Source	F-value	p-value	
Mean vs Total			
Linear vs Mean	2.32	0.1316	
2FI vs Linear	3.78	0.0589	Suggested
Quadratic vs 2FI	1.17	0.4090	
Cubic vs Quadratic	2.65	0.2856	Aliased

ANOVA results for 2FI model to evaluate PS are presented in Table 9. The overall model significance was determined with a p-value of 0.0394 ($p < 0.05$). Additionally, the very large F-value of 3.93 for the model as a whole suggests that large differences between the means exist. The fitness of the model to reproduce the experimental data is also attested from the non-significant lack-of-fit ($p = 0.3141$). Among the individual factors, Span 80 (factor B) affected PS most strongly, as evidenced by its p-value of 0.0249.

Table 9: ANOVA for 2FI model (Response 2: PS)

Source	F-value	P-value	
Model	3.93	0.0394	Significant
A-Stearic Acid	3.95	0.0821	
B-Span 80	7.59	0.0249	
C-Tween 80	0.7049	0.4255	
AB	3.54	0.0966	
AC	3.32	0.1058	
BC	4.48	0.0673	
Lack of Fit	2.49	0.3141	not significant

As indicated from Table 10, the main impact of stearic acid (A) has a positive coefficient of +163.97; nevertheless, its partial contribution is not statistically important considering the broad confidence interval (−26.26 to 354.21), as seen in Figure 7(C). Moreover, Span 80 exhibits a positive main effect of +227.21 with a confidence interval that does not contain zero, showing its significant contribution in enhancing PS. This is confirmed by the trend apparent in the one-factor plot in Figure 7(B) and the three-dimensional surface plot displays a high degree of upward curvature along the Span 80 axis, which demonstrates that higher levels of Span 80 are associated with a substantial rise in PS (Fig.8). Moreover, the contour diagram in Figure (9) aids in explaining the interaction between Span 80 and PS as shown, the color scheme ranges from blue, for small particles, to red, for large particles, thereby graphically confirming that PS increases proportionally with the amount of Span 80. This may be due to the low HLB value (~4.3) of Span 80 which results in less stable emulsions with reduced steric stabilization and less interfacial tension control. According to Shahraeini et al.[43], drug molecules tend to diffuse out of smaller particles with bigger surface area. An increase in Span 80 thus decreases the overall HLB of the surfactant system, thereby promoting particle growth. Similarly, it was reported that surfactants with greater HLB values such as Poloxamer 188 (HLB ~29) created much smaller nanoparticles compared to those with lower HLB values, likely due to their greater emulsification efficiency and steric stabilization capacity [44]. Furthermore, data from another study [45] revealed that PS growth was obvious when the HLB value was reduced below 10.06. Although the effect of Tween 80 is not statistically significant, its negative main effect (-69.26), visible in Table 10 and presented in Figure 7 (A), indicates a possible potential to reduce PS.

Table 10: Coefficients in terms of coded factors regarding EE%

Factor	Coefficient Estimate	95% CI Low	95% CI High
Intercept	569.86	430.93	708.79
A-Stearic Acid	163.97	-26.26	354.21
B-Span 80	227.21	36.98	417.45
C-Tween 80	-69.26	-259.50	120.97
AB	219.58	-49.46	488.61
AC	-212.62	-481.66	56.41
BC	-246.80	-515.83	22.23

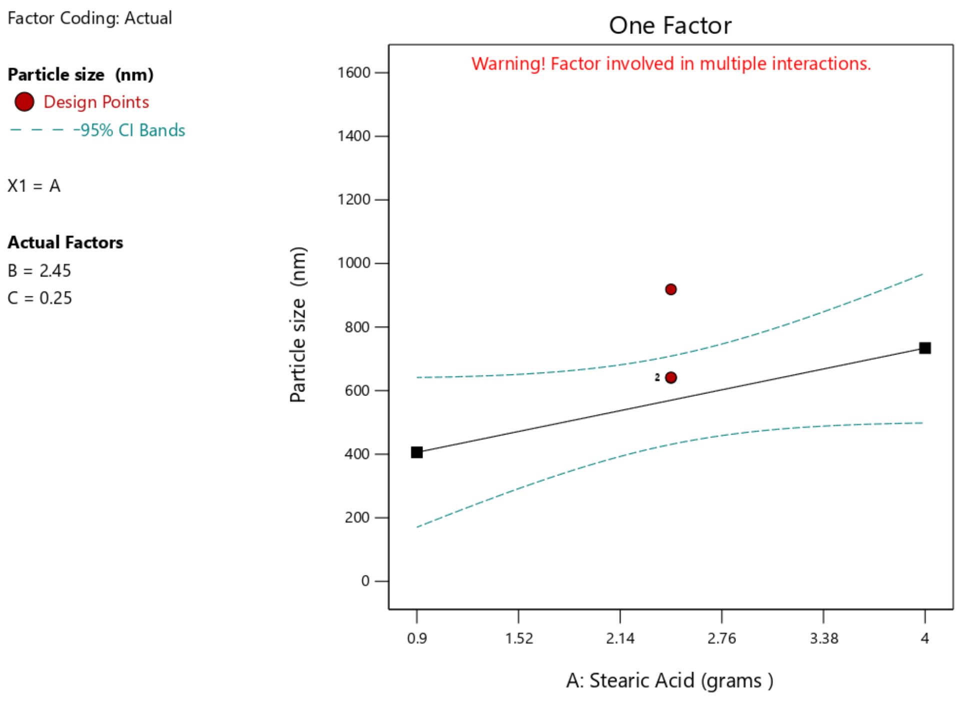

(A)

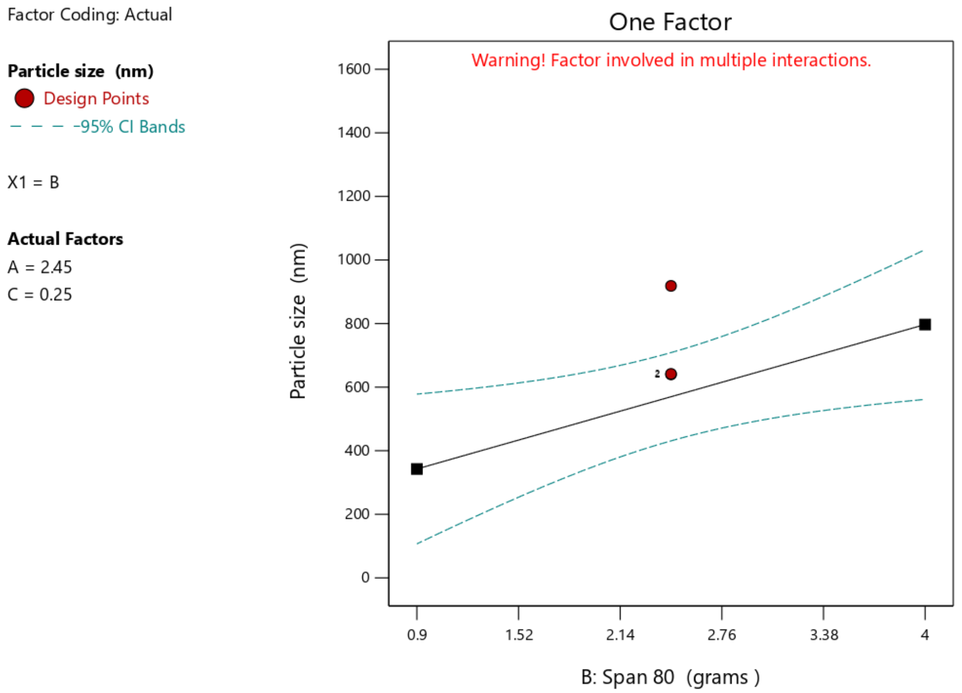

(B)

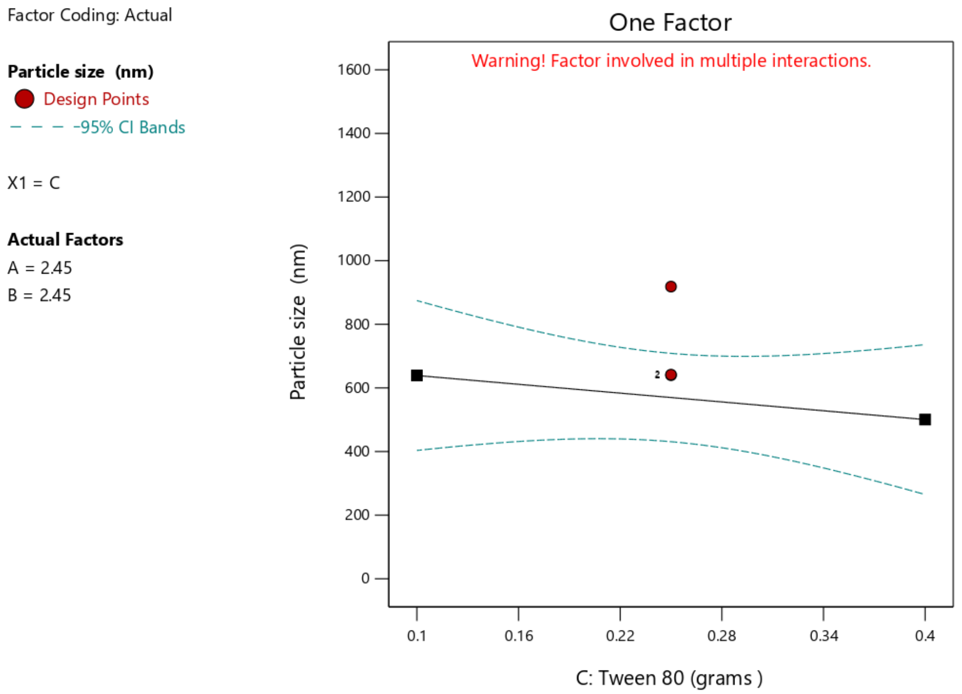

(C)

Figure 7: Line plot for the effect of Stearic acid amount (A), Span 80 amount (B), and Tween 80 amount (C), on the PS

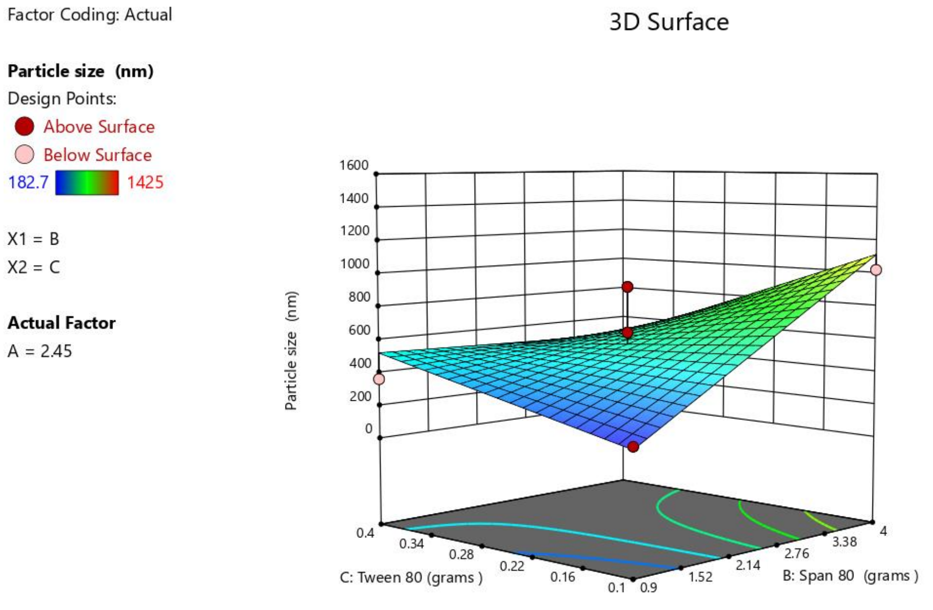

Figure 8: The 3D surface plot of main effect of Span 80 amount and Tween 80 amount on PS

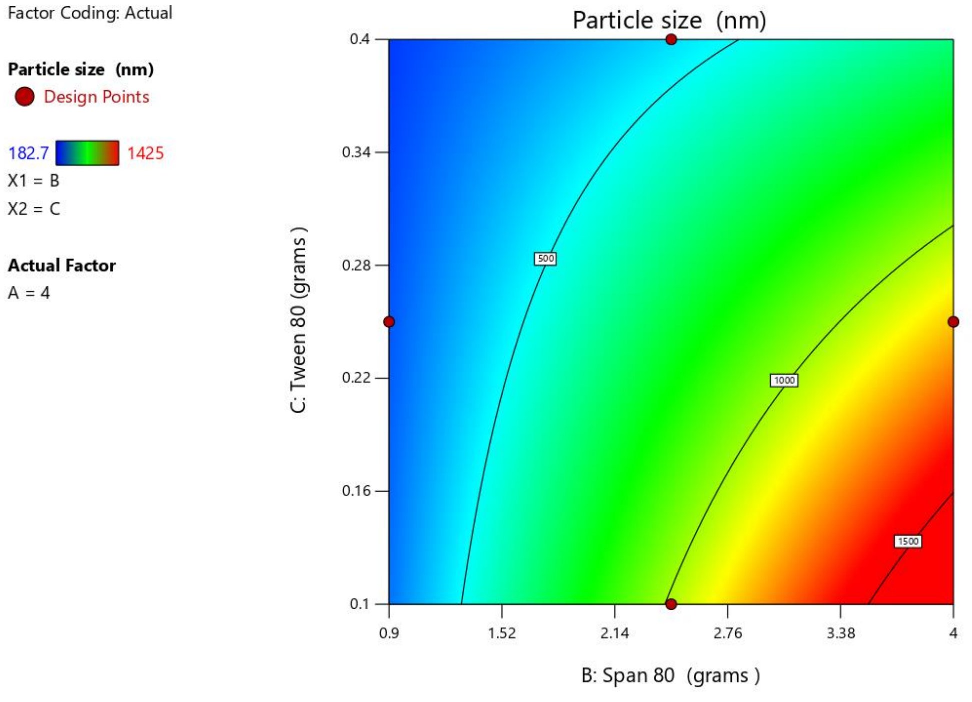

Figure 9: Contour plot of main effect of Tween 80 amount and Span 80 amount on PS

4.3. Impact of independent factors on ZP (Response 3-Y3)

ZP values indicate the stability of formed solid nanoparticles. The higher the ZP (whether positive or negative), the stronger the electrostatic repulsion, and consequently better stability. The charge presented on the surface of nanoparticles depends on the nature of the ingredients used in their preparation. The zeta potential of the prepared nanoparticles ranged from -29.6 ± 1.6 in T2 to -59.0 ± 3.0 in T8 as can be seen in Table 11. This negative charge was attributed to the use of stearic acid as solid lipid in the preparation method. As in aqueous solution stearic acid dissociates, resulting in negatively charged particles. This is because the stearic acid structure has a terminal carboxylic group (-COOH) that gets ionizes to ($-COO^-$) in aqueous media [35,46,47].

Table 11: Mean values of ZP for all trials

	Factor 1	Factor 2	Factor 3	Response 3
**Trials**	**A: Stearic Acid**	**B: Span 80**	**C: Tween 80**	**ZP**
	Grams	Grams	Grams	mV
T1	2.45	2.45	0.25	-58.1 ± 2.9
T2	2.45	0.9	0.1	-29.6 ± 1.6
T3	4	2.45	0.4	-54.4 ± 2.7
T4	2.45	4	0.1	-31.3 ± 1.5
T5	2.45	2.45	0.25	-57.3 ± 2.9
T6	4	4	0.25	-29.8 ± 1.7
T7	0.9	0.9	0.25	-30.9 ± 1.6
T8	2.45	2.45	0.25	-59.0 ± 3.0
T9	0.9	2.45	0.4	-44.6 ± 2.2
T10	4	2.45	0.1	-33.1 ± 1.6
T11	2.45	0.9	0.4	-38.0 ± 1.9
T12	4	0.9	0.25	-48.3 ± 2.5
T13	0.9	2.45	0.1	-45.2 ± 2.2
T14	0.9	4	0.25	-45.4 ± 2.3
T15	2.45	4	0.4	-36.8 ± 1.8

The best fitting model for the ZP analysis was quadratic as illustrated in Table 12. The p-value was **< 0.0001** and an R^2 value of 0.9928. The lack of fit of the model was insignificant compared to the pure error, as indicated by the p-value of 0.1773. A significant relationship among the independent and dependent variables was clearly indicated by the lack of fit, which was not significant ($p > 0.05$). In addition, this lack of fit p-value of 0.1773 which was more than 0.05 declared the model adequacy and significantly. The regression equation of the model was as follows

$$ZP = -58.133 + 0.062A + 0.437B - 4.325C + 8.25AB - 5.475AC + 0.725BC + 4.565A^2 + 14.967B^2 + 9.242C^2 \quad \text{(Equation 4)}$$

Table 12: Proposed model for observed ZP

Source	F-value	P-value	
Mean vs Total			
Linear vs Mean	0.3681	0.7776	
2FI vs Linear	0.9454	0.4631	
Quadratic vs 2FI	154.63	< 0.0001	Suggested
Cubic vs Quadratic	4.80	0.1773	Aliased

The data in Table 13 indicates the ANOVA results of model. The p-value (< 0.0001) was below 0.05, this confirms the statistically significant model. In this case C, AB, AC, A^2, B^2, C^2 were significant model terms. Values greater than 0.1000 indicate the model terms were not significant. Further, F-value of 4.80 implies that the Lack of Fit was not significant relative to the pure error

Table 13: ANOVA for Quadratic model (Response 3: ZP)

Source	F-value	p-value	
Model	77.10	< 0.0001	Significant
A-Stearic Acid	0.0132	0.9131	
B-Span 80	0.6456	0.4582	
C-Tween 80	63.09	0.0005	
AB	114.78	0.0001	
AC	50.55	0.0009	
BC	0.8864	0.3897	
A^2	32.46	0.0023	
B^2	348.71	< 0.0001	
C^2	132.96	< 0.0001	
Residual			
Lack of Fit	4.80	0.1773	not significant

The correlation between the stearic acid amount and the ZP was a non-linear relationship. Although the linear coefficient with respect to stearic acid was statistically not significant, the

response curvature was confirmed by the existence of a large quadratic term as indicated in Table 14. The linear term A was + 0.0625 and CI: –1.34 to 1.46 thus insignificant, but the quadratic term A^2 was +4.57 and CI: 2.51 to 6.63 hence significant and indicating curved response as illustrated in Figure 10 (A). The graph depicts that ZP initially becomes increasingly negative with increasing stearic acid amount to a minimum prior to increasing again at higher amount of stearic acid thus forming a U-shaped curve. This indicates that intermediate concentrations of stearic acid result in the most negative values of ZP which was the optimal for stability. A study confirmed that the relation between the amount of stearic acid and ZP value was not linear,[48] stearic acid stabilizes the particles electrostatically by adsorption onto the surface of SLNs, and any deficiency or excess will disturb this equilibrium, wherein ZP was lowered this was an indicator for losing colloidal stability [49].Same as the effect of stearic acid, Span 80 has a quadratic effect on ZP (Table 14). Its highly significant non-linear term indicated the sharp U-shaped correlation where the surface charge of nanoparticles was less negative at low and high concentrations and reached its most negative value at intermediate levels as shown in Figure 10 (B). This was also consistent with the findings of Rostamkalaei et al [45], who found that The ZP of SLNs was usually decreased by increasing the concentration of Span 80. Because of the role of Span 80 which enhances surface charge by producing a more compact surfactant layer around the nanoparticles, reduction of the negative ZP may occur when amounts of Span 80 were above the optimum at intermediate levels. This may potentially reduce the stability of the stabilizing surfactant layer and consequently decrease colloidal stability. Furthermore, Tween 80 exerted the most influence on ZP of the three independent variables. The linear term has a highly negative coefficient, meaning that higher Tween 80 amount leads to more negative ZP and, thus, better stabilization of the colloid (see Table 14). This confirmed through the 3D structure in Figure (11) and in contour plot in Figure (12). Furthermore, the presence of a significant quadratic term indicates that the relationship was not linear in the sense that ZP initially reaches a maximum value before it falls at higher concentrations. This behavior was shown in the accompanying Figure 10 (C) with a lower minimum compared to that of stearic acid and Span 80, with a characteristic curved response Furthermore, stearic acid also exhibited a notable negative interaction with Tween 80, indicative of a strong interactive [50]. Collectively, these findings demonstrate the critical role played by Tween 80 in the modulation of surface charge and the conferment of stability to the nanoparticles produced. This aligned with Shi et al [14] findings that increasing the amount of Tween 80 will increase the zeta potential values. This might be due to the hydrophobic chain length of Tween 80, compared to other Tweens, leading to more compact packaging of hydrophobic chains in the oil phase. On addition of the microemulsion to cold water, the oil phase becomes the solid lipid matrix as in SLNs. More dense packing of these hydrophobic chains yields a more rigid lipid core. Hence, Tween 80 enhances the structural rigidity and overall stability of the SLNs.

Table 14: Coefficients in terms of coded factors regarding ZP

Factor	Coefficient Estimate	95% CI Low	95% CI High
Intercept	-58.13	-60.42	-55.85
A-Stearic acid	0.0625	-1.34	1.46
B-Span 80	0.4375	-0.9622	1.84
C-Tween 80	-4.33	-5.72	-2.93
AB	8.25	6.27	10.23
AC	-5.48	-7.45	-3.50
BC	0.7250	-1.25	2.70
A^2	4.57	2.51	6.63
B^2	14.97	12.91	17.03
C^2	9.24	7.18	11.30

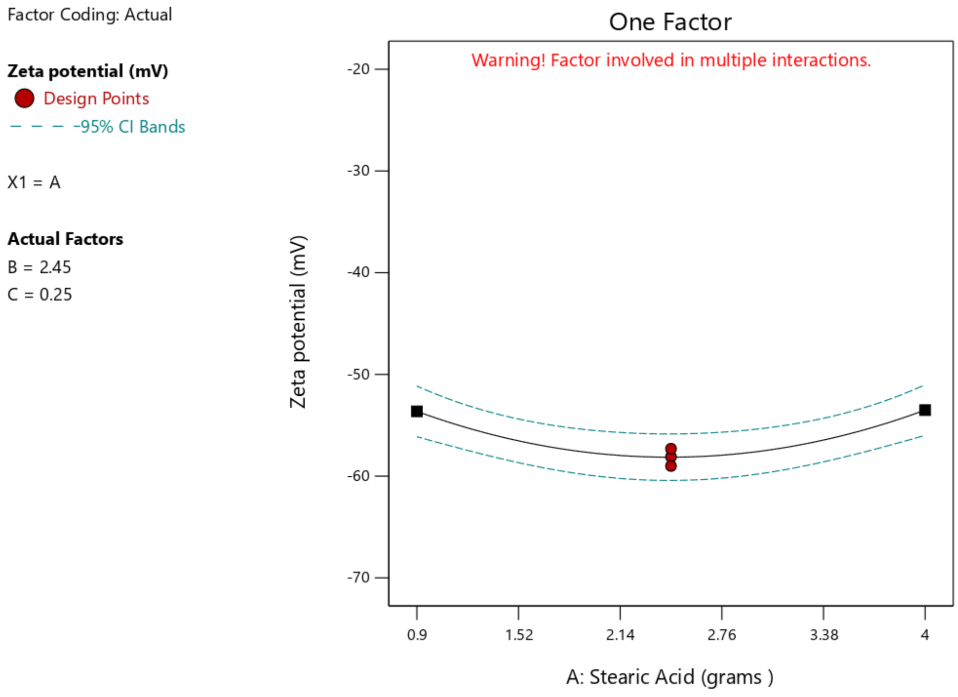
Factor Coding: Actual
Zeta potential (mV)
Design Points
-95% CI Bands
X1 = A
Actual Factors
B = 2.45
C = 0.25
One Factor
Warning! Factor involved in multiple interactions.
Zeta potential (mV)
-20
-30
-40
-50
-60
-70
0.9
1.52
2.14
2.76
3.38
4
A: Stearic Acid (grams)

(A)

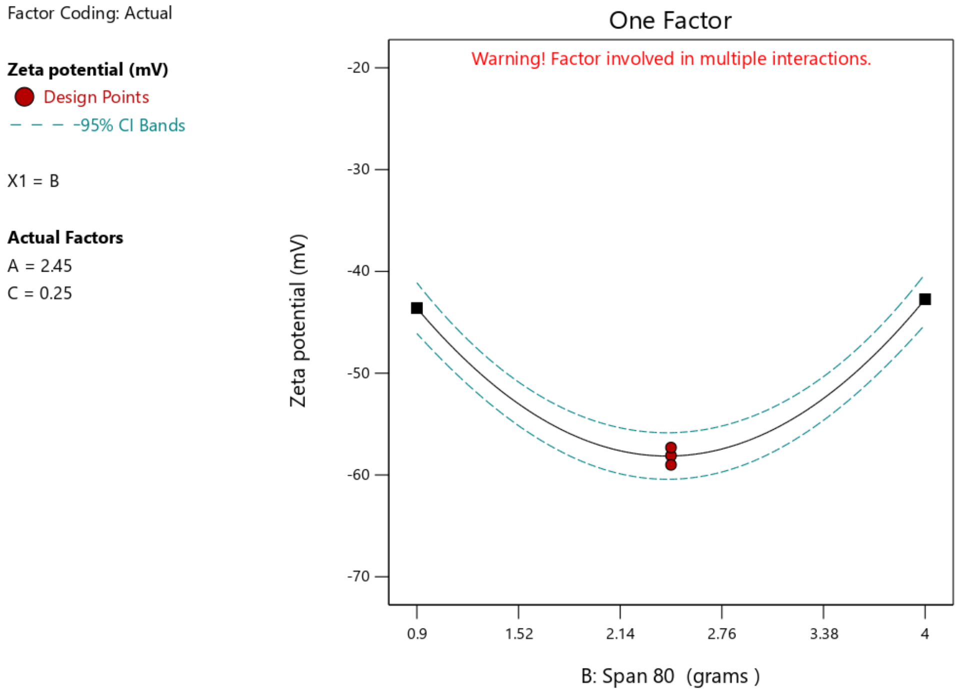
Factor Coding: Actual
Zeta potential (mV)
Design Points
-95% CI Bands
X1 = B
Actual Factors
A = 2.45
C = 0.25
One Factor
Warning! Factor involved in multiple interactions.
Zeta potential (mV)
-20
-30
-40
-50
-60
-70
0.9
1.52
2.14
2.76
3.38
4
B: Span 80 (grams)

(B)

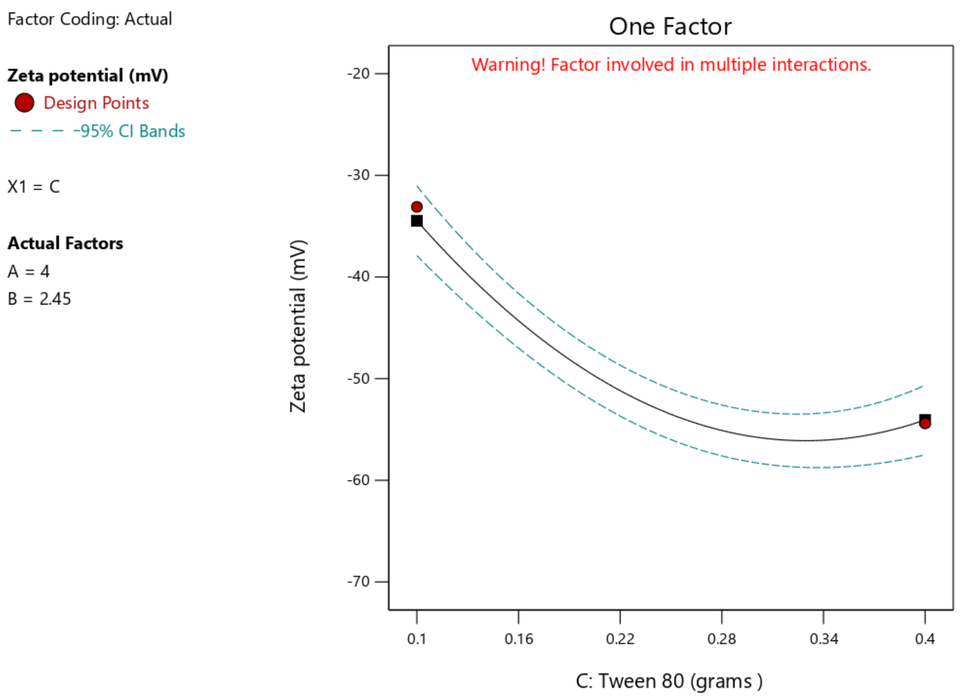

(C)

Figure 10: Line plot for the effect of Stearic acid amount (A), Span 80 amount (B), and Tween 80 amount (C) on ZP

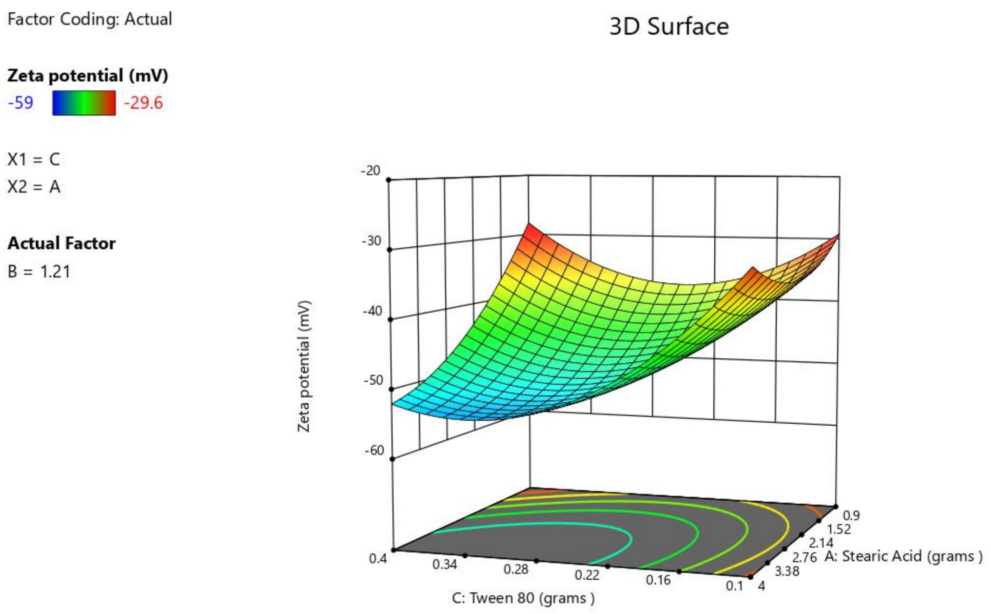

Figure 11: 3D surface plot of main effect of Stearic acid amount and Tween 80 amount on ZP

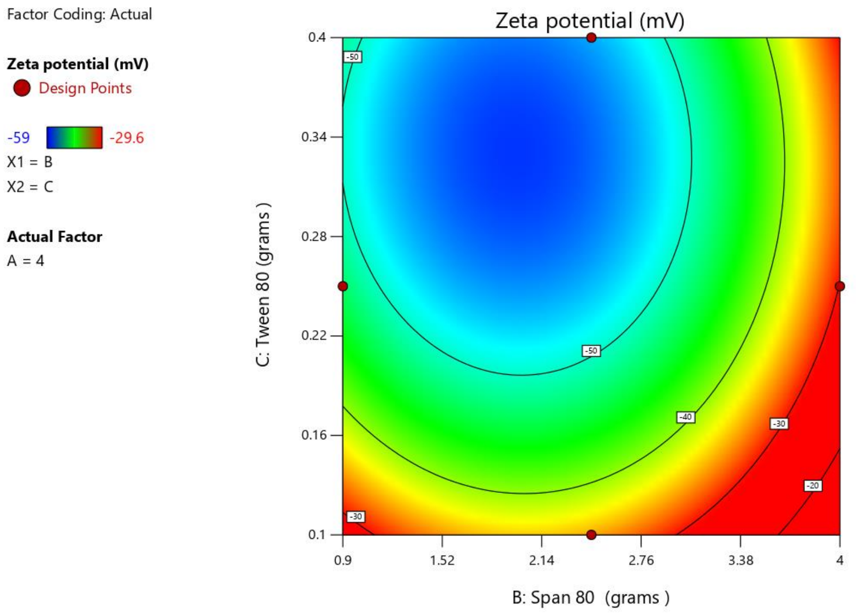

Figure 12: Contour plot of main effect of Tween 80 amount and Span 80 amount on ZP

5.Optimization

Design Expert® software was used to statistically optimize MOX-SLNs preparation by performing desirability function calculations and statistical analysis that assist in establishing the optimal combination of independent variables for achieving the desired outcomes. The desirability

function, ranging from 0 to 1, indicates the extent to which the selected factor levels meet the objectives set, with closer to 1 value indicating an improved solution. In this work, restraints were applied to optimize ZP, PS, and encapsulation efficiency (EE%). Based on these parameters, the software had recommended A F-opt with a total desirability value of 0.851. Table 16 contains the specific factor values corresponding to the F-opt. This formula was further characterized under the same conditions for testing the efficiency of the optimization process.

Table 16: Predicted levels for F-opt and desirability

Independent Variable	Levels	Desirability
A-Stearic acid	4	0.851
B-Span 80	0.9	
C-Tween 80	0.3371	

5.1. Assessment of F-opt

5.1.1.PS, ZP and EE% results

As previously indicated EE%, PS, and ZP were measured, with values summarized in Table 17. The applicability of the optimization process in respect to accuracy and reliability was authenticated by the fact that experimentally obtained values for each response EE%, PS, and ZP fill within their confidence intervals.

Table 17: F-opt Predicted and observed responses

F-opt	Predicted Mean	Observed	95% CI low for Mean	95% CI high for Mean
EE (%)	81.7055	79.563	74.4227	89.565
PS (nm)	290.126	257.7	177.677	810.001
ZP (mv)	-55.7538	-52.4	-55.7099	-50.2881

5.1.2. Microscopical characteristics

The surface morphology and structural characteristics of the F-opt were examined using both SEM and TEM. Regarding TEM images (Fig.13) evidently showed discrete, nearly spherical nanoparticles with smooth surface. In addition, it revealed a tightly packed group of smaller, shape-isomorphic particles that were closely packed in contact to yield a cohesive network. This clumping behavior is characteristic for surfactant-stabilized lipid nanoparticles and suggests excellent particle homogeneity and surface integrity. The other alternative, SEM images, provides additional information on the surface morphology (Fig.14). The particles with heterogeneous, irregular shapes were observed, potentially suggestive of minor aggregation or layering effects. In addition, it showed an expanded field of view with multiple spherical particles dispersed across

the surface. The irregular, non-smooth surface textures are evident and may be due to by stearic acid crystallization during freeze-drying, a process that has been reported in SLNs preparations and may be accountable for surface roughness as well as for enhancing rearrangement of the lipid matrix [51]. Furthermore, an irregular surface morphology was observed having large surface features presumably caused by crystallized lipid domains, was observed. Combined, these SEM characteristics validate the formation of the SLNs and were consistent with earlier studies demonstrating that stearic acid-based SLNs would have rough or flaky surfaces following lyophilization, elucidating the role of lipid crystallization impacts on the morphological characteristics of the nanoparticles [52]

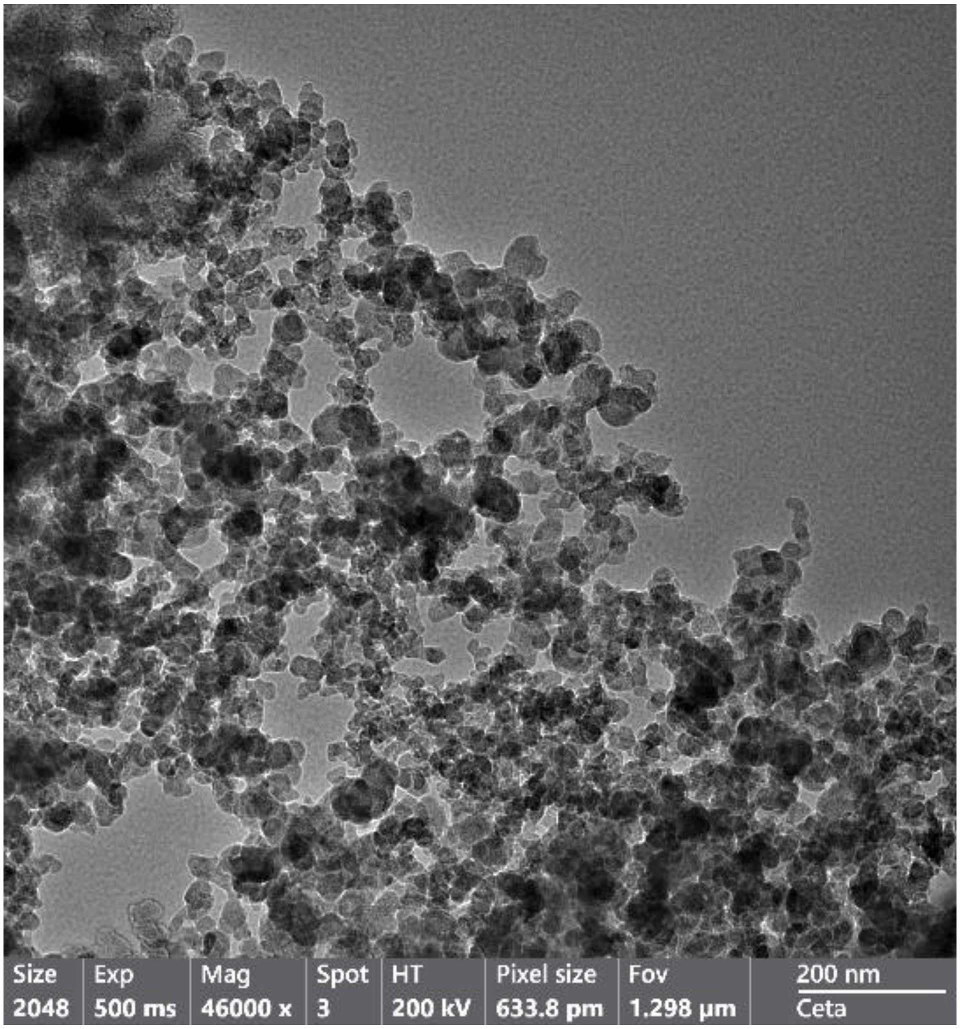

Figure 13: TEM images of F-opt

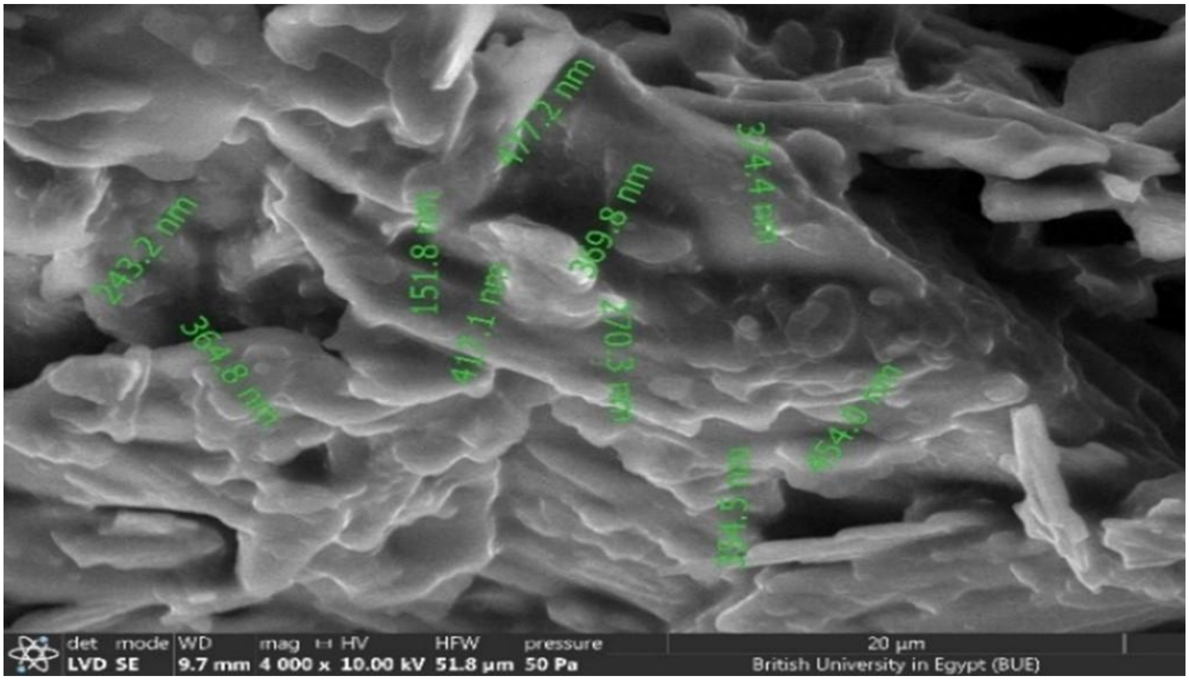

Figure 14: SEM image of the lyophilized F-opt

5.1.3. X-ray diffraction (XRD)

The crystallinity of the pure drug, stearic acid, physical mixture, and the prepared nanoparticles were investigated through XRD (Figure 15). The pure MOX exhibited sharp diffraction peaks, confirming that it was a crystalline substance. Stearic acid also displayed characteristic peaks, confirming that it exists in a crystalline state[53]. In the physical mixture, peaks attributable to both the drug and stearic acid could be seen, indicating that simple mixture does not trigger significant physicochemical interactions. However, the XRD spectrum of the nanoparticles showed a decrease in the peak intensity of stearic acid, as well as the complete disappearance of some of the drug's characteristic peaks. This implies that in the matrix of the nanoparticle, drug exists in the form of amorphous or molecularly dispersed drug with no crystalline structure as further illustrated in the overlaid patterns in Figure 16.

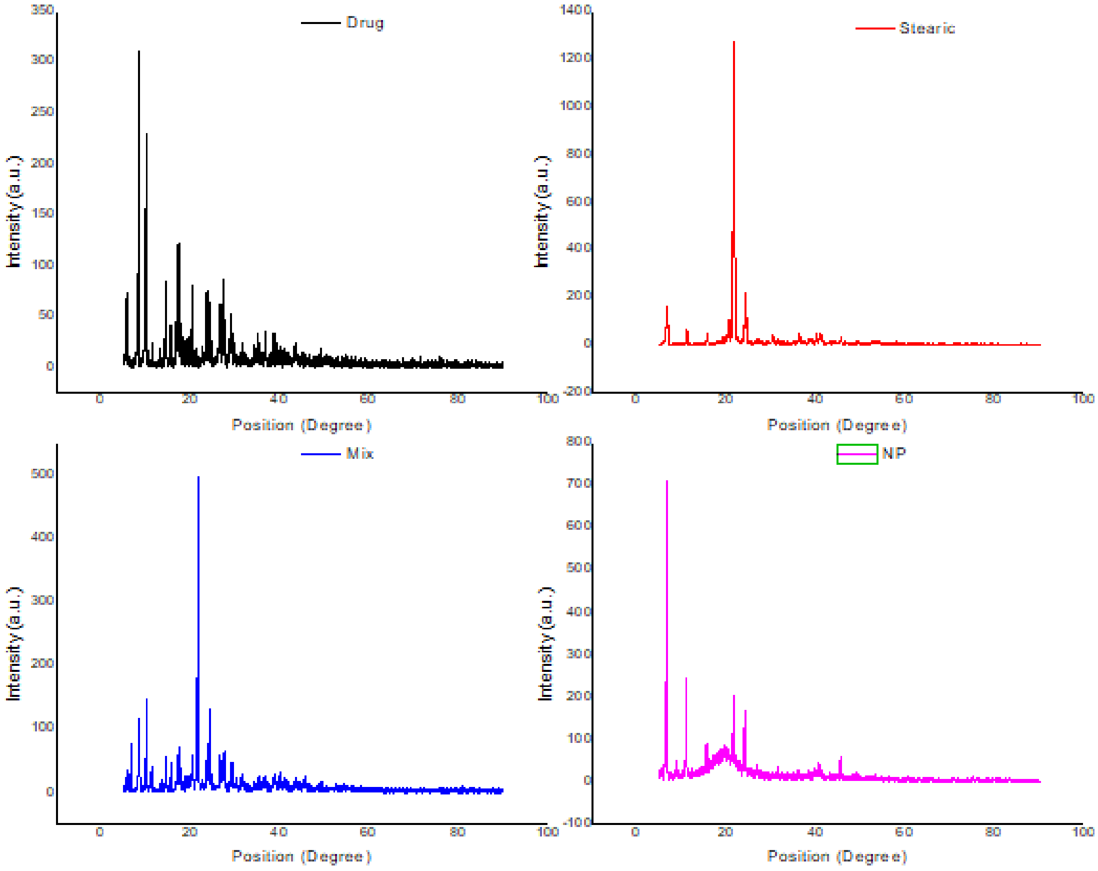

Figure 15: XRD of pure drug, stearic acid, physical mixture of drug: stearic 1:1 and F-opt

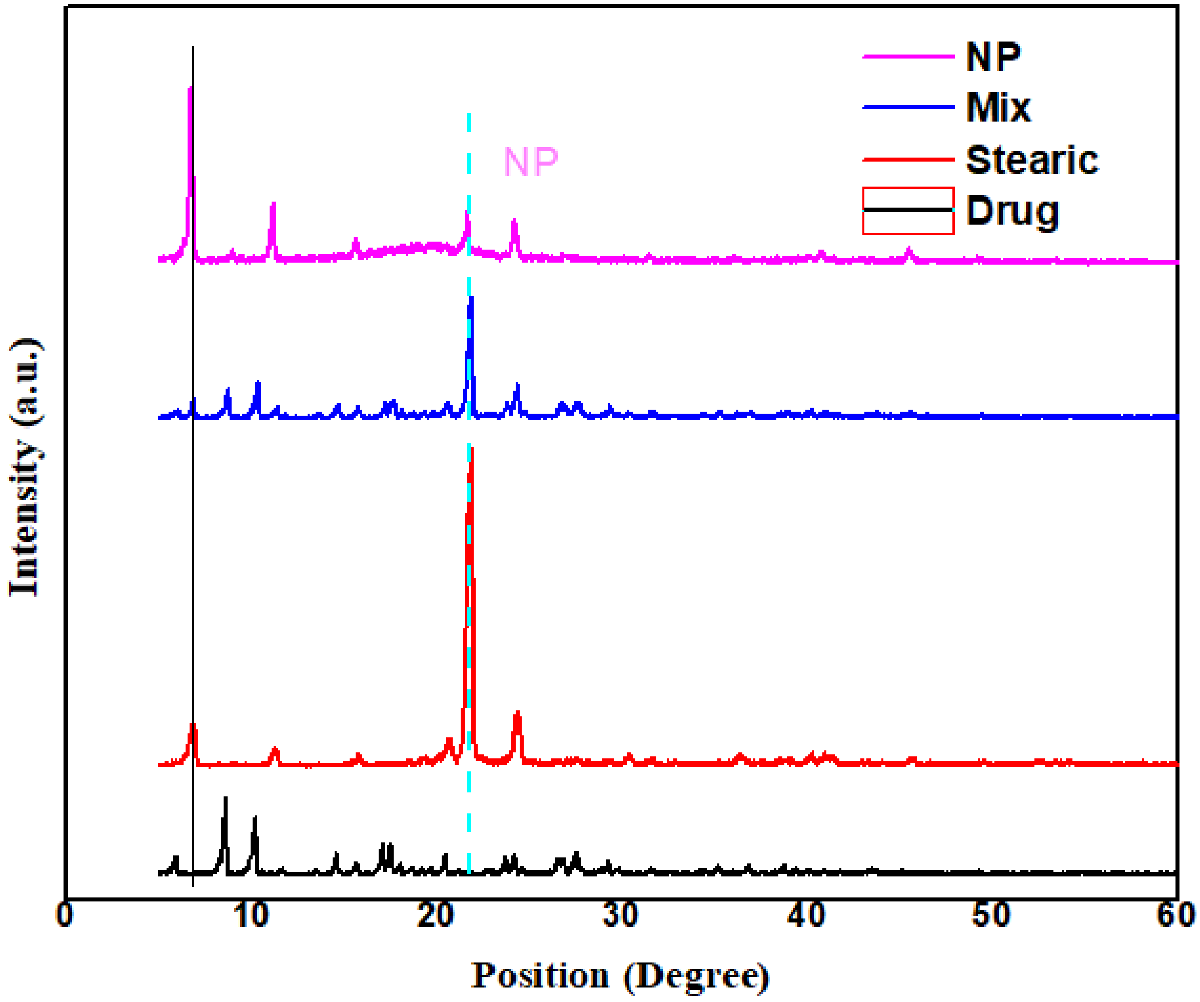

Figure 16: XRD stacked lines for pure drug, stearic acid, physical mixture of drug: stearic 1:1 and F-opt

5.1.4. Fourier transform infrared (FTIR) spectroscopy

As shown in Figure 17 (A), FTIR is for the pure drug and for F-opt (B). Furthermore, (C) indicates no marked changes or disappearance of the prominent peaks in between the F-opt and the pure drug, indicating neither chemical interaction nor chemical bond formation between MOX and stearic acid. This information supports the evidence that the drug was largely encapsulated in or physically embedded into the lipid matrix of the solid SLNs rather than any chemical modification [54]

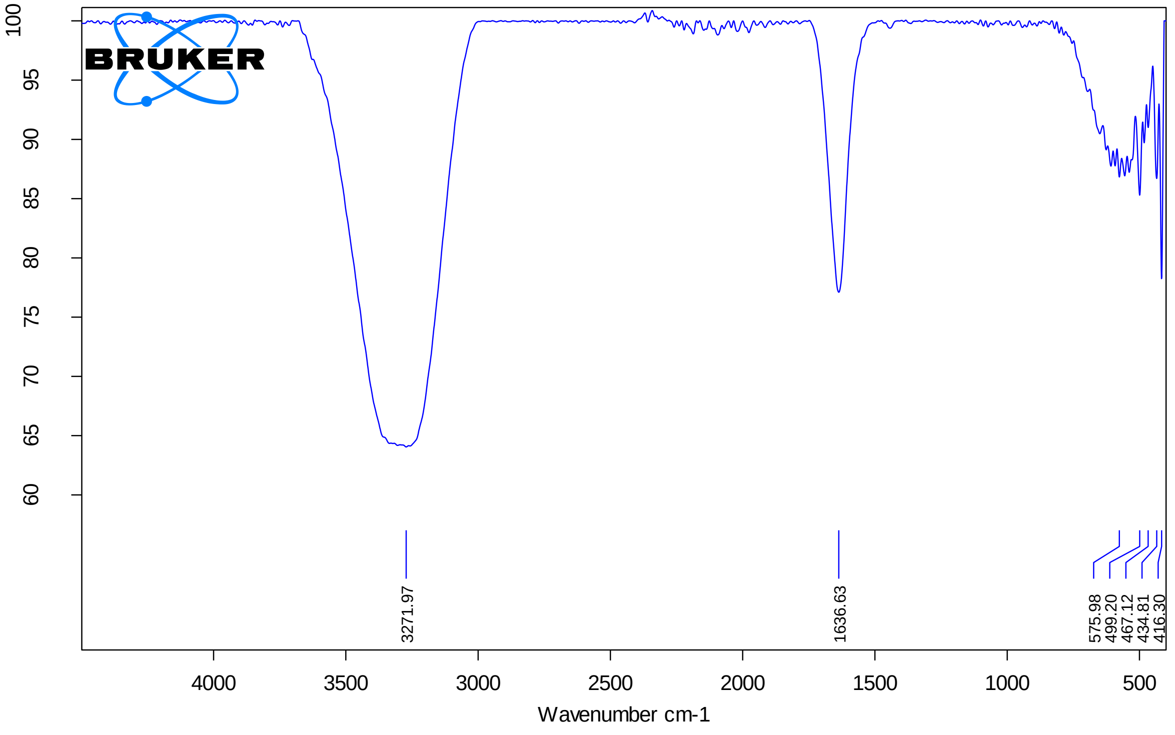
BRUKER
100
95
90
85
80
75
70
65
60
3271.97
1636.63
575.98
499.20
467.12
434.81
416.30
4000
3500
3000
2500
2000
1500
1000
500
Wavenumber cm-1

(A)

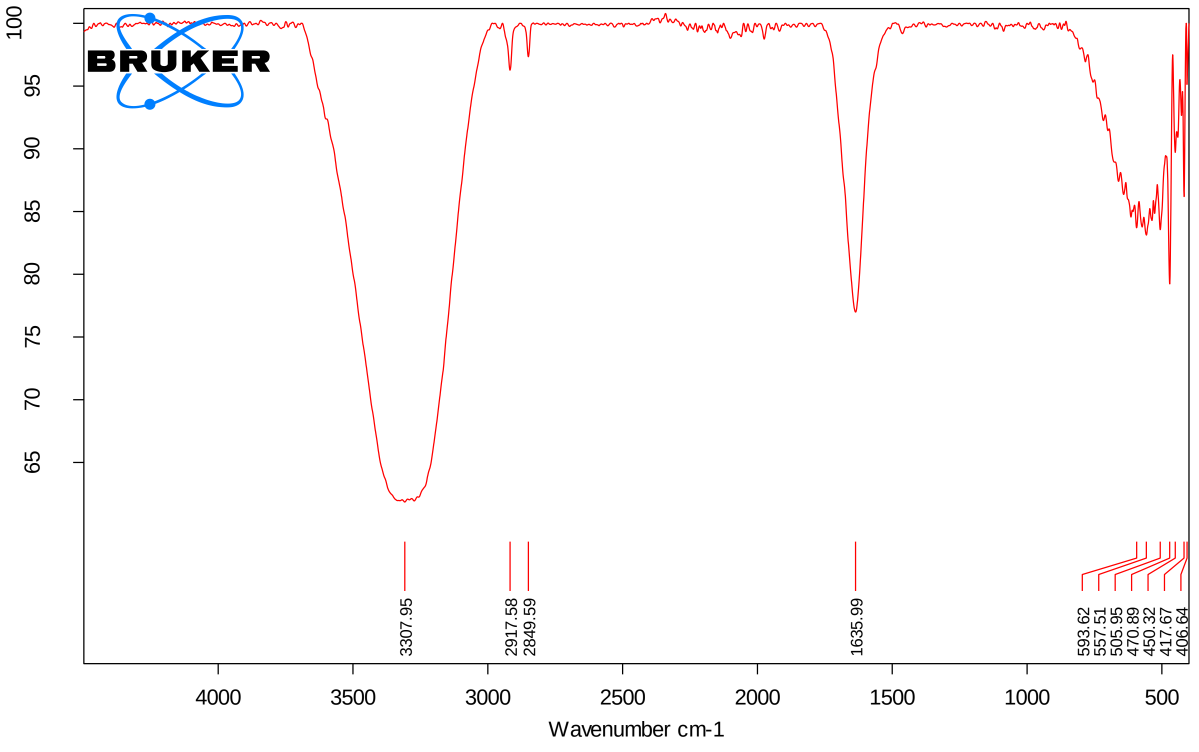
BRUKER
100
95
90
85
80
75
70
65
3307.95
2917.58
2849.59
1635.99
593.62
557.51
505.95
470.89
450.32
417.67
406.64
4000
3500
3000
2500
2000
1500
1000
500
Wavenumber cm-1

(B)

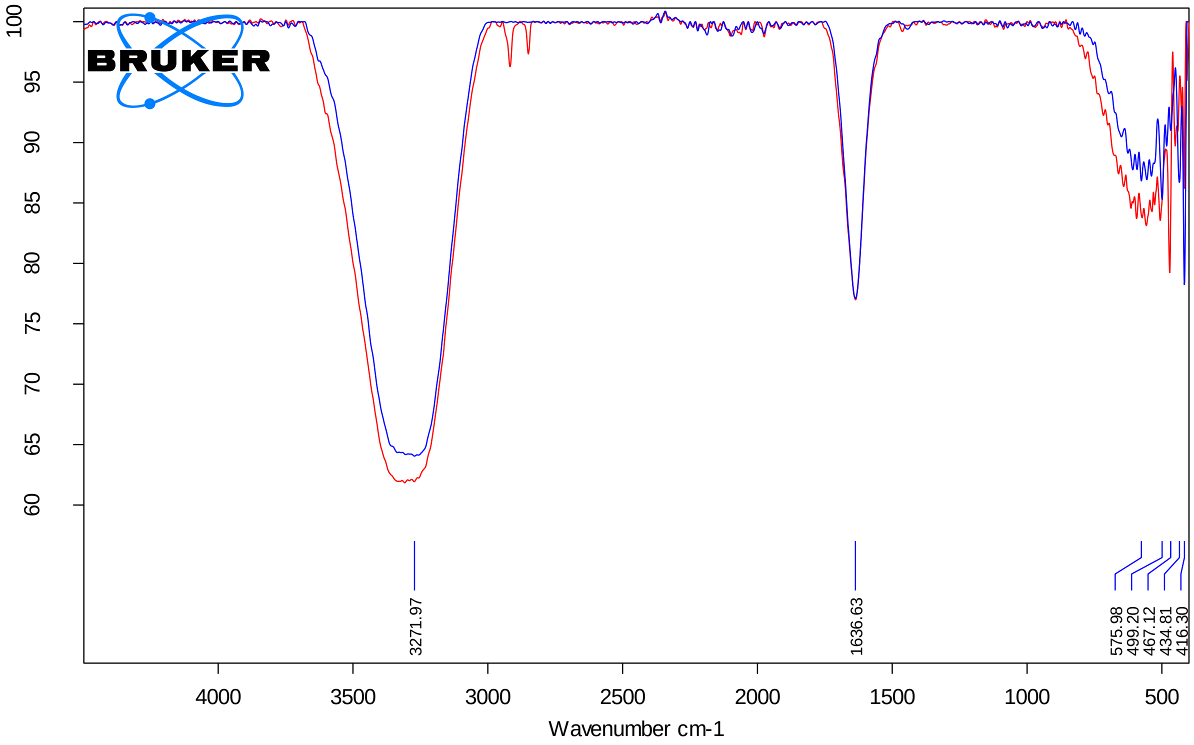

(C)

Figure 17: FTIR chart of pure drug (MOX) (A), F-opt (B) and the overlaid of both charts (A) and (B) in (C)

Conclusion

In conclusion, this research effectively synthesized and optimized MOX-SLNs through a sequential process in line with the Box–Behnken experimental design. The method permitted a detailed assessment of the principal variables in preparation and their impact on performance parameters namely, EE, ZP, and PS. SEM and TEM characterization showed that the uniform morphology and spherical structure of the nanoparticles were exhibited consistently. FTIR and XRD analyses confirmed the physical encapsulation mechanism in the lipid matrix, with no chemical interaction among drug and excipients. The physicochemical properties of optimized SLNs (F-opt) confirm its potential for application as a topical delivery system for the treatment of chronic wounds. Drug release, in vivo potency, and cytotoxicity studies are warranted to determine the stability, biocompatibility, and clinical significance of the SLN preparations.

Funding sources

This research did not receive any specific grant from funding agencies in the public, commercial, or not-for-profit sectors

Credit authorship contribution statement

Esraa M. Elshazly conducted the experimental research, analysed the data and wrote the original draft, **Mona G. Arafa** suggested the research idea**,** contributed to reviewing, editing, supervision and conceptualization, **Samia A. Nour** Participating in reviewing and refine the manuscript and contributed to conceptualization, visualization and supervision

Declaration of Competing Interest

The authors declare that they have no known competing financial interests or personal relationships that could have appeared to influence the work reported in this paper.

References

1. Barnum, L., Samandari, M., Schmidt, T. A. & Tamayol, A. Microneedle arrays for the treatment of chronic wounds. *Expert Opin. Drug Deliv.* **17**, 1767–1780 (2020).
2. Arroyave, F., Montaño, D. & Lizcano, F. Diabetes Mellitus Is a Chronic Disease that Can Benefit from Therapy with Induced Pluripotent Stem Cells. *Int. J. Mol. Sci. 2020, Vol. 21, Page 8685* **21**, 8685 (2020).
3. Martino, G., Caputo, A., Bellone, F., Quattropani, M. C. & Vicario, C. M. Going beyond the visible in type 2 diabetes mellitus: Defense mechanisms and their associations with depression and health-related quality of life. *Front. Psychol.* **11**, 519034 (2020).
4. Lipsky, B. A. *et al.* Guidelines on the diagnosis and treatment of foot infection in persons with diabetes (IWGDF 2019 update). *Diabetes. Metab. Res. Rev.* **36**, e3280 (2020).
5. Robertson, S. M. *et al.* Ocular Pharmacokinetics of Moxifloxacin After Topical Treatment of Animals and Humans. *Surv. Ophthalmol.* **50**, S32–S45 (2005).
6. Ezhilarasu, H., Vishalli, D., Dheen, S. T., Bay, B. H. & Kumar Srinivasan, D. Nanoparticle-based therapeutic approach for diabetic wound healing. *Nanomaterials* **10**, 1–29 (2020).
7. Mihai, M. M., Dima, M. B., Dima, B. & Holban, A. M. Nanomaterials for Wound Healing and Infection Control. *Mater. 2019, Vol. 12, Page 2176* **12**, 2176 (2019).
8. Rajendran, N. K., Kumar, S. S. D., Houreld, N. N. & Abrahamse, H. A review on nanoparticle based treatment for wound healing. *J. Drug Deliv. Sci. Technol.* **44**, 421–430 (2018).
9. Mohammed, H. A. *et al.* Solid lipid nanoparticles for targeted natural and synthetic drugs delivery in high-incidence cancers, and other diseases: Roles of preparation methods, lipid composition, transitional stability, and release profiles in nanocarriers' development. *Nanotechnol. Rev.* **12**, (2023).

10. Abosabaa, S. A., Elmeshad, A. N. & Arafa, M. G. Chitosan nanocarrier entrapping hydrophilic drugs as advanced polymeric system for dual pharmaceutical and cosmeceutical application: A comprehensive analysis using box–behnken design. *Polymers (Basel).* **13**, 1–18 (2021).

11. Ding, S., Serra, C. A., Vandamme, T. F., Yu, W. & Anton, N. Double emulsions prepared by two–step emulsification: History, state-of-the-art and perspective. *J. Control. Release* **295**, 31–49 (2019).

12. Abdel Hady, M., Sayed, O. M. & Akl, M. A. Brain uptake and accumulation of new levofloxacin-doxycycline combination through the use of solid lipid nanoparticles: Formulation; Optimization and in-vivo evaluation. *Colloids Surfaces B Biointerfaces* **193**, 111076 (2020).

13. Chhibber, T., Gondil, V. S. & Sinha, V. R. Development of Chitosan-Based Hydrogel Containing Antibiofilm Agents for the Treatment of Staphylococcus aureus–Infected Burn Wound in Mice. *AAPS PharmSciTech* **21**, 2–13 (2020).

14. Shi, L., Li, Z., Yu, L., Jia, H. & Zheng, L. Effects of Surfactants and Lipids on the Preparation of Solid Lipid Nanoparticles Using Double Emulsion Method. *J. Dispers. Sci. Technol.* **32**, 254–259 (2011).

15. Khairnar, S. V. *et al.* Review on the Scale-Up Methods for the Preparation of Solid Lipid Nanoparticles. *Pharm. 2022, Vol. 14, Page 1886* **14**, 1886 (2022).

16. Nandanwar, M. *et al.* Assessment of wound healing efficacy of Growth Factor Concentrate (GFC) in non-diabetic and diabetic Sprague Dawley rats. *J. Diabetes Metab. Disord.* **20**, 1583–1595 (2021).

17. Becker Peres, L., Becker Peres, L., de Araújo, P. H. H. & Sayer, C. Solid lipid nanoparticles for encapsulation of hydrophilic drugs by an organic solvent free double emulsion technique. *Colloids Surfaces B Biointerfaces* **140**, 317–323 (2016).

18. Hosseini, S. M. *et al.* Doxycycline-encapsulated solid lipid nanoparticles as promising tool against Brucella melitensis enclosed in macrophage: A pharmacodynamics study on J774A.1 cell line. *Antimicrob. Resist. Infect. Control* **8**, 1–12 (2019).

19. Li, Z., Yu, L., Zheng, L. & Geng, F. Studies on crystallinity state of puerarin loaded solid lipid nanoparticles prepared by double emulsion method. *J. Therm. Anal. Calorim.* **99**, 689–693 (2010).

20. Nandini, P. T., Doijad, R. C., Shivakumar, H. N. & Dandagi, P. M. Formulation and evaluation of gemcitabine-loaded solid lipid nanoparticles. *Drug Deliv.* **22**, 647–651 (2015).

21. Dong, Z. *et al.* Preparation and in vitro, in vivo evaluations of norfloxacin-loaded solid lipid nanopartices for oral delivery. *Drug Deliv.* **18**, 441–450 (2011).

22. Radwan, I. T. *et al.* Effect of nanostructure lipid carrier of methylene blue and monoterpenes as enzymes inhibitor for Culex pipiens. *Sci. Rep.* **13**, 1–15 (2023).

23. Bharti Sharma, J. *et al.* Statistical optimization of tetrahydrocurcumin loaded solid lipid

nanoparticles using Box Behnken design in the management of streptozotocin-induced diabetes mellitus. *Saudi Pharm. J.* **31**, 101727 (2023).

24. Peng, X. *et al.* Box–Behnken design based statistical modeling for the extraction and physicochemical properties of pectin from sunflower heads and the comparison with commercial low-methoxyl pectin. *Sci. Rep.* **10**, 1–10 (2020).

25. Nandanwar, M. *et al.* Assessment of wound healing efficacy of Growth Factor Concentrate (GFC) in non-diabetic and diabetic Sprague Dawley rats. *J. Diabetes Metab. Disord.* **20**, 1583–1595 (2021).

26. Motwani, S. K., Chopra, S., Ahmad, F. J. & Khar, R. K. Validated spectrophotometric methods for the estimation of moxifloxacin in bulk and pharmaceutical formulations. *Spectrochim. Acta Part A Mol. Biomol. Spectrosc.* **68**, 250–256 (2007).

27. Akanda, M., Mithu, M. S. H. & Douroumis, D. Solid lipid nanoparticles: An effective lipid-based technology for cancer treatment. *J. Drug Deliv. Sci. Technol.* **86**, 104709 (2023).

28. Abosabaa, S. A., Arafa, M. G. & ElMeshad, A. N. Hybrid chitosan-lipid nanoparticles of green tea extract as natural anti-cellulite agent with superior in vivo potency: full synthesis and analysis. *Drug Deliv.* **28**, 2160–2176 (2021).

29. Sohail, S. *et al.* Melatonin delivered in solid lipid nanoparticles ameliorated its neuroprotective effects in cerebral ischemia. *Heliyon* **9**, e19779 (2023).

30. Martínez-Acevedo, L. *et al.* Effect of magnesium stearate solid lipid nanoparticles as a lubricant on the properties of tablets by direct compression. *Eur. J. Pharm. Biopharm.* **193**, 262–273 (2023).

31. Elgendy, K. H., Zaky, M., altorky, A. E. mohamed M. & Fadel, S. Determination of levofloxacin, norfloxacin, and moxifloxacin in pharmaceutical dosage form or individually using derivative UV spectrophotometry. *BMC Chem.* **18**, 1–21 (2024).

32. Barnum, L., Samandari, M., Schmidt, T. A. & Tamayol, A. Microneedle arrays for the treatment of chronic wounds. *Expert Opin. Drug Deliv.* **17**, 1767–1780 (2020).

33. Peters, E. J. G. *et al.* Interventions in the management of infection in the foot in diabetes: a systematic review. *Diabetes. Metab. Res. Rev.* **36**, e3282 (2020).

34. Singh, S., Dobhal, A. K., Jain, A., Pandit, J. K. & Chakraborty, S. Formulation and Evaluation of Solid Lipid Nanoparticles of a Water Soluble Drug: Zidovudine. *Chem. Pharm. Bull.* **58**, 650–655 (2010).

35. Subroto, E., Andoyo, R., Indiarto, R., Wulandari, E. & Wadhiah, E. F. N. Preparation of Solid Lipid Nanoparticle-Ferrous Sulfate by Double Emulsion Method Based on Fat Rich in Monolaurin and Stearic Acid. *Nanomater. 2022, Vol. 12, Page 3054* **12**, 3054 (2022).

36. Silpa, R., Chakravarthi, N., Chandramouli, Y. & Hemanth pavan kumar, K. Moxifloxacin loaded solid lipid nanoparticles (SLNs): preparation and characterization. *Asian J. Pharm. Res.* **2**, 105–112 (2012).

37. Darsh, G., Himanshu, C. & Ranjit, S. Lomefloxacin loaded solid lipid nanoparticles gel for topical ocular therapy: Optimization, evaluation and ex vivo studies. *Res. J. Chem. Environ.* **26**, 14–22 (2022).

38. Bhatt, S. *et al.* Design and Optimization of Febuxostat-loaded Nano Lipid Carriers Using Full Factorial Design. *Turkish J. Pharm. Sci.* **18**, 61 (2021).

39. Yao, S. *et al.* Size-dependence of the skin penetration of andrographolide nanosuspensions: In vitro release-ex vivo permeation correlation and visualization of the delivery pathway. *Int. J. Pharm.* **641**, 123065 (2023).

40. Xiang, H. *et al.* Skin permeation of curcumin nanocrystals: Effect of particle size, delivery vehicles, and permeation enhancer. *Colloids Surfaces B Biointerfaces* **224**, 113203 (2023).

41. Sainaga Jyothi, V. G. S. *et al.* Lipid nanoparticles in topical dermal drug delivery: Does chemistry of lipid persuade skin penetration? *J. Drug Deliv. Sci. Technol.* **69**, 103176 (2022).

42. Yao, S. *et al.* Size-dependence of the skin penetration of andrographolide nanosuspensions: In vitro release-ex vivo permeation correlation and visualization of the delivery pathway. *Int. J. Pharm.* **641**, 123065 (2023).

43. Shahraeini, S. S. *et al.* Atorvastatin Solid Lipid Nanoparticles as a Promising Approach for Dermal Delivery and an Anti-inflammatory Agent. *AAPS PharmSciTech* **21**, 1–10 (2020).

44. Ekambaram, P. & Abdul Hasan Sathali, A. Formulation and Evaluation of Solid Lipid Nanoparticles of Ramipril. *J. Young Pharm.* **3**, 216–220 (2011).

45. Rostamkalaei, S. S., Akbari, J., Saeedi, M., Morteza-Semnani, K. & Nokhodchi, A. Topical gel of Metformin solid lipid nanoparticles: A hopeful promise as a dermal delivery system. *Colloids Surfaces B Biointerfaces* **175**, 150–157 (2019).

46. Badawi, N. M. *et al.* Pomegranate extract-loaded solid lipid nanoparticles: Design, optimization, and in vitro cytotoxicity study. *Int. J. Nanomedicine* **13**, 1313–1326 (2018).

47. Shah, R. M. *et al.* Transport of stearic acid-based solid lipid nanoparticles (SLNs) into human epithelial cells. *Colloids Surfaces B Biointerfaces* **140**, 204–212 (2016).

48. Shah, R., Eldridge, D. S., Palombo, E. & Harding, I. Optimisation and stability assessment of solid lipid nanoparticles using particle size and zeta potential. (2014).

49. Shah, R. M., Eldridge, D. S., Palombo, E. A. & Harding, I. H. Stability mechanisms for microwave-produced solid lipid nanoparticles. *Colloids Surfaces A Physicochem. Eng. Asp.* **643**, 128774 (2022).

50. Ramadan, S. E., El-Gizawy, S. A., Osman, M. A. & Arafa, M. F. Application of Design of Experiment in the Optimization of Apixaban-Loaded Solid Lipid Nanoparticles: In Vitro and In Vivo Evaluation. *AAPS PharmSciTech* **24**, 1–13 (2023).

51. Cassayre, M. *et al.* Optimization of Solid Lipid Nanoparticle Formulation for Cosmetic Application Using Design of Experiments, PART II: Physical Characterization and In

Vitro Skin Permeation for Sesamol Skin Delivery. *Cosmet. 2024, Vol. 11, Page 120* **11**, 120 (2024).

52. Wang, J. *et al.* Solid lipid nanoparticles as an effective sodium aescinate delivery system: formulation and anti-inflammatory activity. *RSC Adv.* **12**, 6583–6591 (2022).

53. Behbahani, E. S., Ghaedi, M., Abbaspour, M. & Rostamizadeh, K. Optimization and characterization of ultrasound assisted preparation of curcumin-loaded solid lipid nanoparticles: Application of central composite design, thermal analysis and X-ray diffraction techniques. *Ultrason. Sonochem.* **38**, 271–280 (2017).

54. P., G. A., V., M. H. & S., P. C. Effective management of odontogenic infections through controlled fashion by polymeric device containing Moxyfloxacin. *Int. J. Curr. Pharm. Res.* 100–106 (2017) doi:10.22159/ijcpr.2017v9i5.22149.
