# Supplementary material for: Development and optimization of Moxifloxacin solid lipid nanoparticles via double emulsion organic solvent free technique applying Box–Behnken experimental design
Source: Sci Rep. 2025 Nov 26;15:42013. doi: 10.1038/s41598-025-26860-x (PMC12657925; doi:10.1038/s41598-025-26860-x)

Trial 1

System

**Temperature (°C):**	25.0	**Duration Used (s):**	60
**Count Rate (kcps):**	338.7	**Measurement Position (mm):**	5.50
**Cell Description:**	Clear disposable zeta cell	**Attenuator:**	7

Results

			Size (d.nm):	% Intensity:	St Dev (d.n...
**Z-Average (d.nm):**	627.5	**Peak 1:**	493.4	69.8	90.09
**PdI:**	0.577	**Peak 2:**	101.1	30.2	19.65
**Intercept:**	0.976	**Peak 3:**	0.000	0.0	0.000

Result quality : Refer to quality report

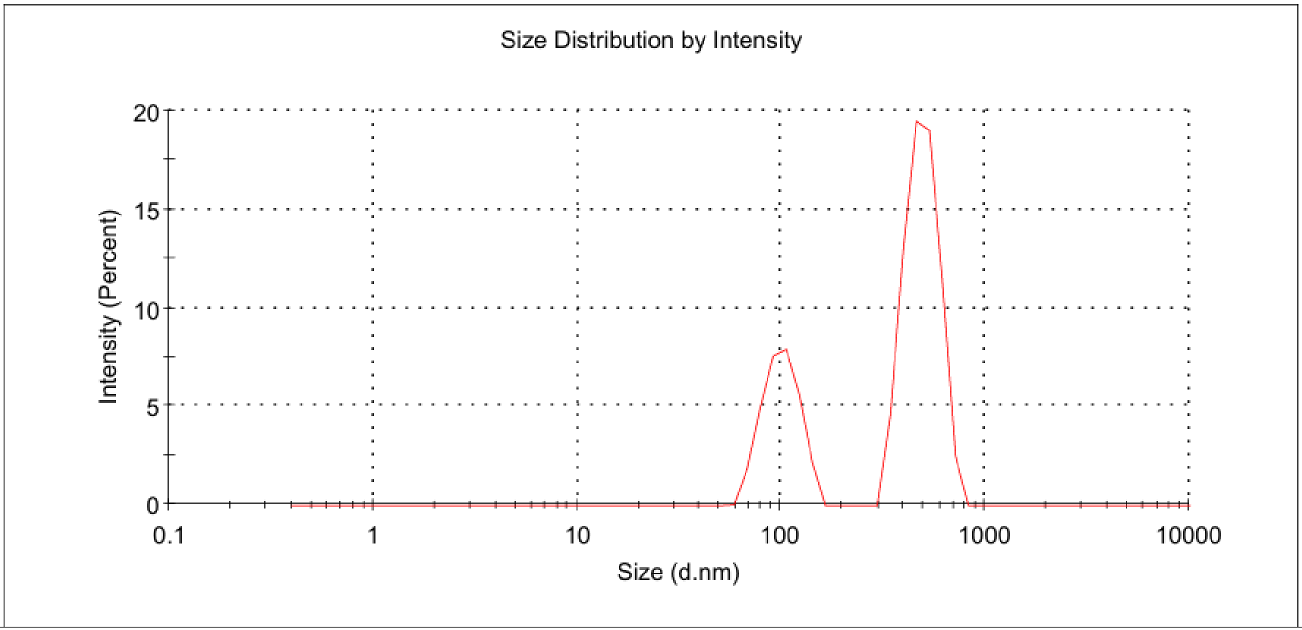

Trial 2

System

**Temperature (°C):**	25.0	**Duration Used (s):**	60
**Count Rate (kcps):**	304.4	**Measurement Position (mm):**	5.50
**Cell Description:**	Clear disposable zeta cell	**Attenuator:**	6

Results

			Size (d.nm):	% Intensity:	St Dev (d.n...
**Z-Average (d.nm):**	182.7	**Peak 1:**	181.4	98.8	86.71
**PdI:**	0.424	**Peak 2:**	5560	1.2	0.000
**Intercept:**	0.967	**Peak 3:**	0.000	0.0	0.000

Result quality : Good

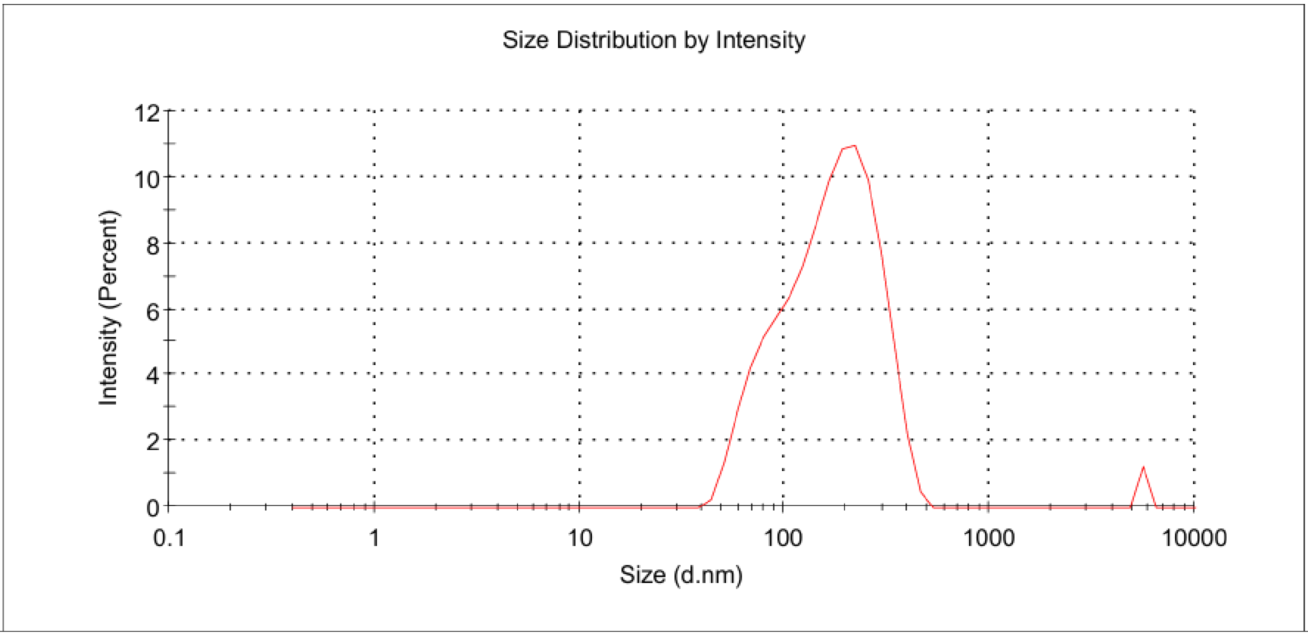

Trial 3

System

**Temperature (°C):**	25.0	**Duration Used (s):**	90
**Count Rate (kcps):**	53.9	**Measurement Position (mm):**	5.50
**Cell Description:**	Clear disposable zeta cell	**Attenuator:**	5

Results

			Size (d.nm):	% Intensity:	St Dev (d.n...
**Z-Average (d.nm):**	360.0	**Peak 1:**	504.3	67.2	133.0
**PdI:**	0.559	**Peak 2:**	103.3	32.8	24.72
**Intercept:**	0.971	**Peak 3:**	0.000	0.0	0.000

Result quality : **Refer to quality report**

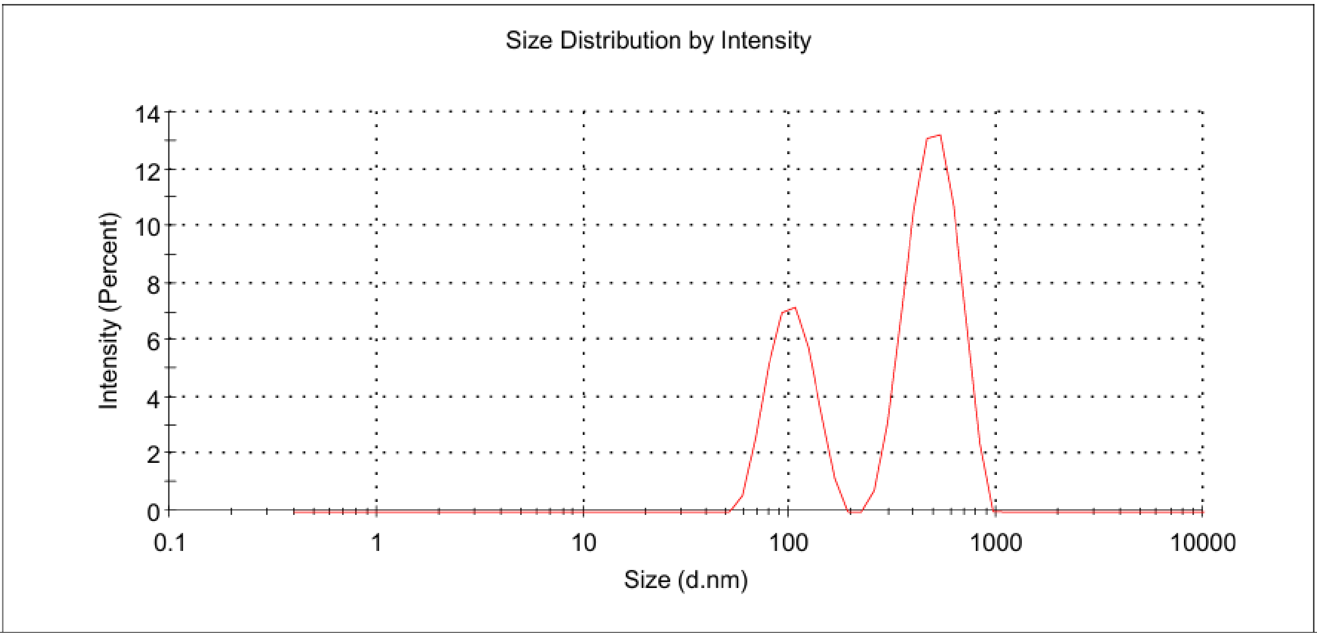

Trial 4

System

**Temperature (°C):**	25.0	**Duration Used (s):**	60
**Count Rate (kcps):**	327.7	**Measurement Position (mm):**	5.50
**Cell Description:**	Clear disposable zeta cell	**Attenuator:**	6

Results

			Size (d.nm):	% Intensity:	St Dev (d.n...
**Z-Average (d.nm):**	1020	**Peak 1:**	821.7	87.8	165.4
**PdI:**	0.556	**Peak 2:**	121.5	12.2	17.74
**Intercept:**	0.774	**Peak 3:**	0.000	0.0	0.000

Result quality : Refer to quality report

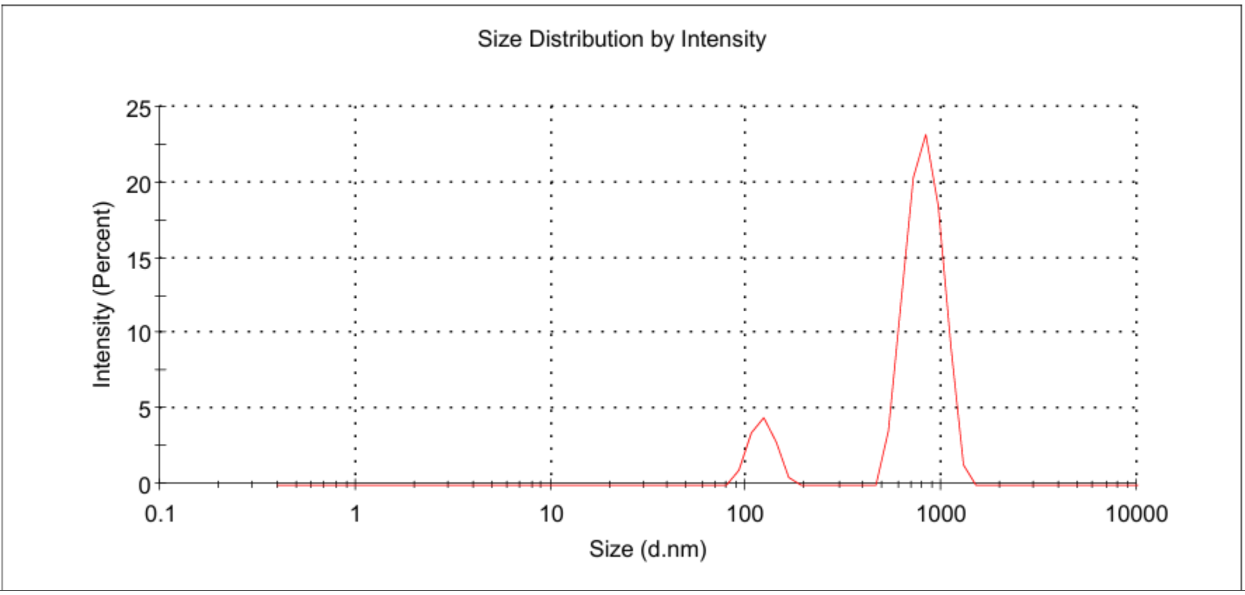

Trial 5

System

**Temperature (°C):**	25.0	**Duration Used (s):**	80
**Count Rate (kcps):**	179.1	**Measurement Position (mm):**	5.50
**Cell Description:**	Clear disposable zeta cell	**Attenuator:**	7

Results

			Size (d.nm):	% Intensity:	St Dev (d.n...
**Z-Average (d.nm):**	912.2	**Peak 1:**	636.1	74.9	135.0
**PdI:**	0.888	**Peak 2:**	129.2	25.1	33.34
**Intercept:**	0.942	**Peak 3:**	0.000	0.0	0.000

Result quality : Refer to quality report

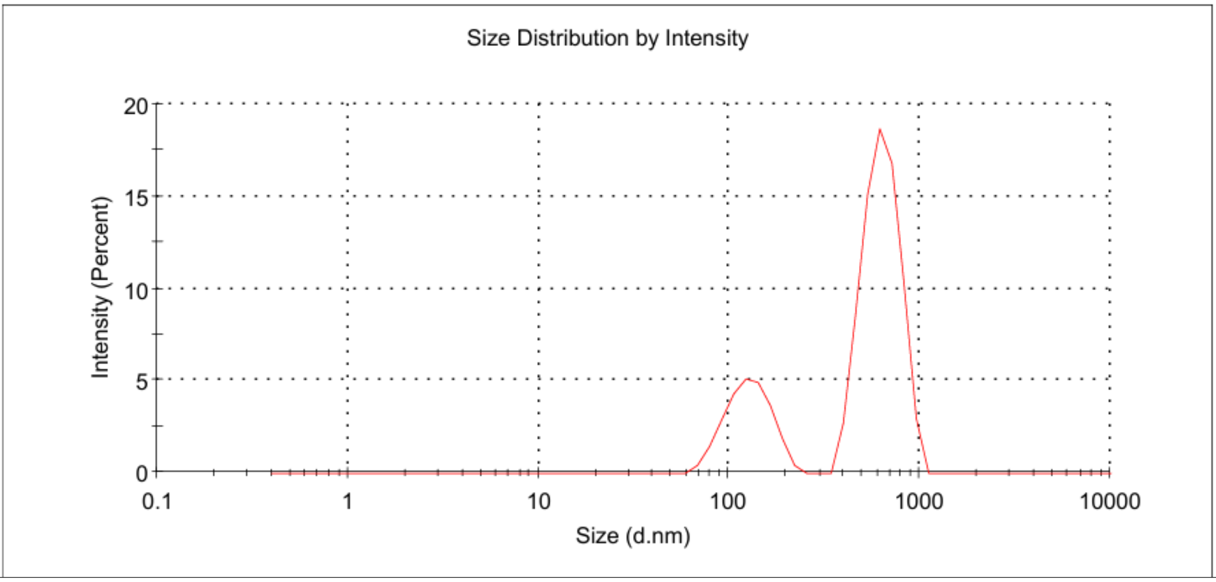

Trial 6

System

**Temperature (°C):**	25.0	**Duration Used (s):**	70
**Count Rate (kcps):**	143.6	**Measurement Position (mm):**	5.50
**Cell Description:**	Clear disposable zeta cell	**Attenuator:**	5

Results

			Size (d.nm):	% Intensity:	St Dev (d.n...
**Z-Average (d.nm):**	1425	**Peak 1:**	445.4	53.0	82.99
**PdI:**	1.000	**Peak 2:**	5371	36.8	328.1
**Intercept:**	0.855	**Peak 3:**	109.1	10.1	14.34

Result quality : **Refer to quality report**

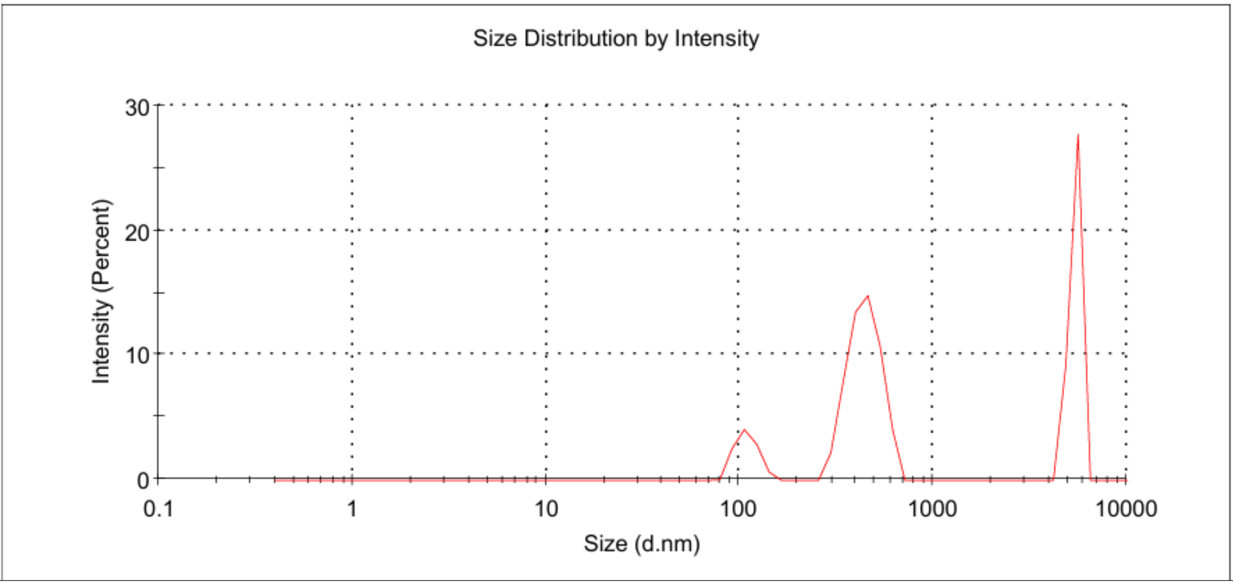

Trial 7

System

**Temperature (°C):**	25.0	**Duration Used (s):**	60
**Count Rate (kcps):**	451.1	**Measurement Position (mm):**	5.50
**Cell Description:**	Clear disposable zeta cell	**Attenuator:**	7

Results

			Size (d.nm):	% Intensity:	St Dev (d.n...
**Z-Average (d.nm):**	272.9	**Peak 1:**	136.1	49.0	55.95
**PdI:**	0.830	**Peak 2:**	4623	26.9	840.5
**Intercept:**	0.837	**Peak 3:**	404.1	24.1	129.1

Result quality : Refer to quality report

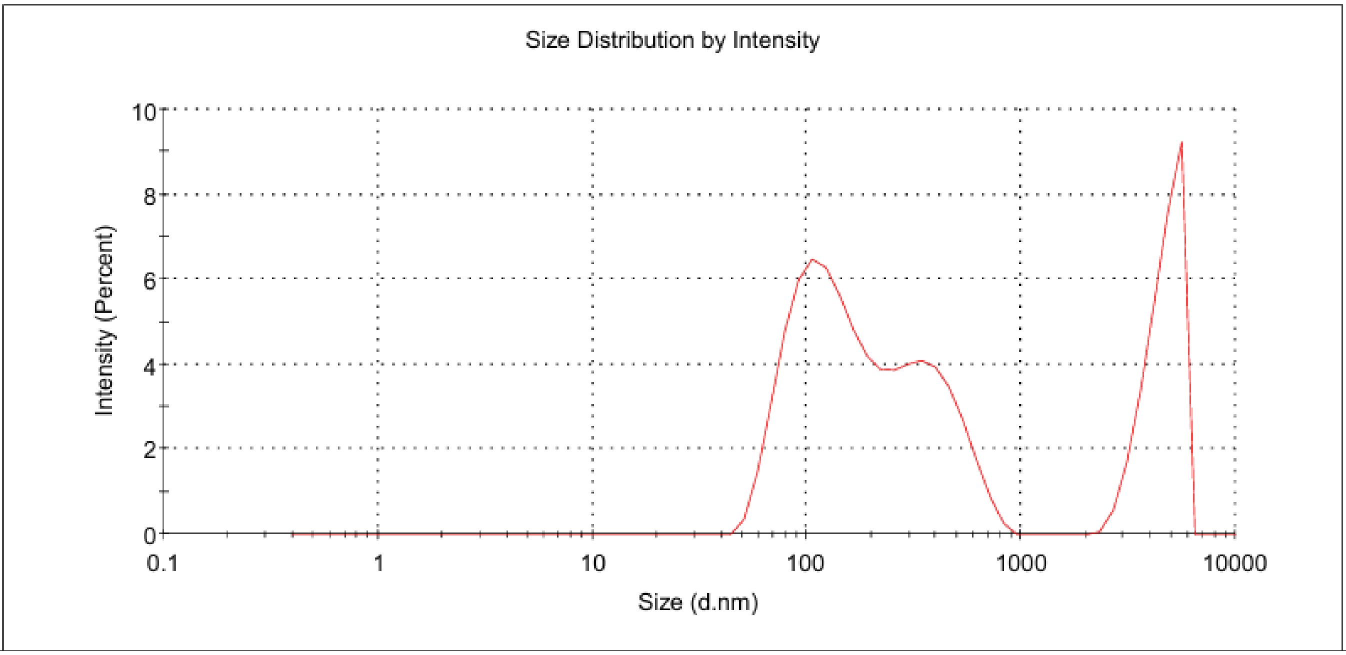

Trial 8

System

**Temperature (°C):**	25.0	**Duration Used (s):**	70
**Count Rate (kcps):**	134.4	**Measurement Position (mm):**	5.50
**Cell Description:**	Clear disposable zeta cell	**Attenuator:**	6

Results

			Size (d.nm):	% Intensity:	St Dev (d.n...
**Z-Average (d.nm):**	638.9	**Peak 1:**	153.0	100.0	21.80
**PdI:**	0.654	**Peak 2:**	0.000	0.0	0.000
**Intercept:**	0.838	**Peak 3:**	0.000	0.0	0.000

Result quality : **Refer to quality report**

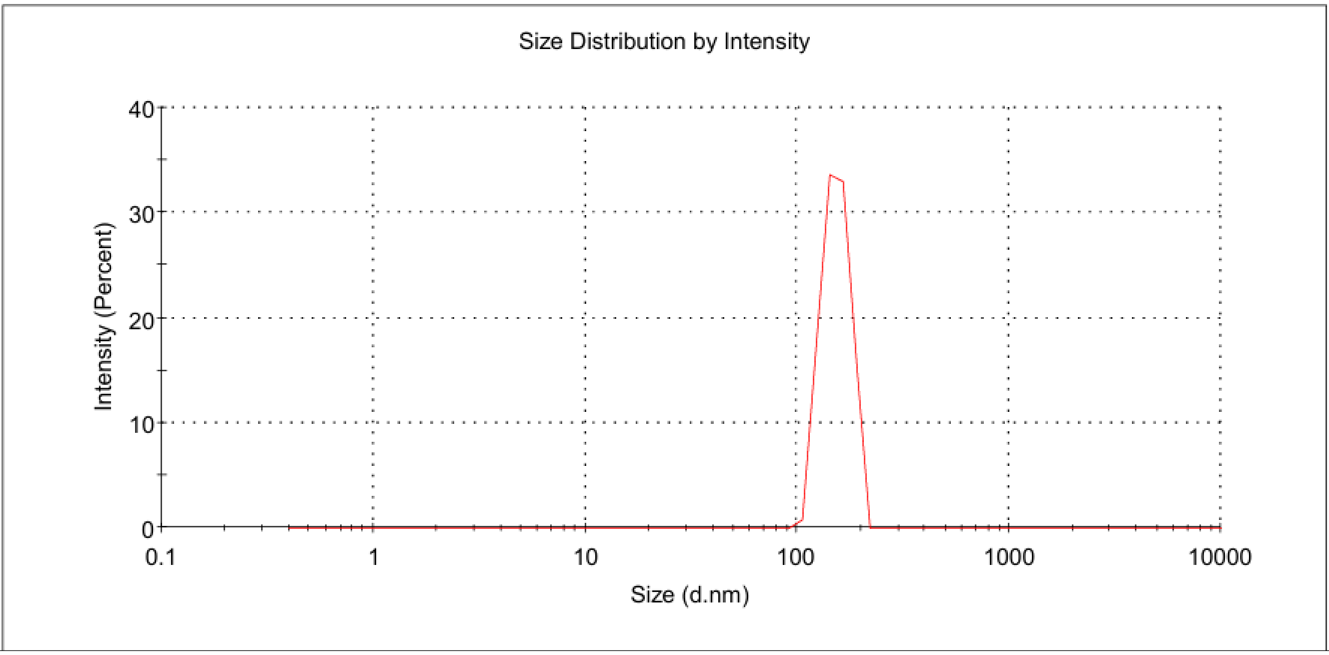

Trial 9

System

**Temperature (°C):**	25.0	**Duration Used (s):**	60
**Count Rate (kcps):**	248.0	**Measurement Position (mm):**	5.50
**Cell Description:**	Clear disposable zeta cell	**Attenuator:**	6

Results

			Size (d.nm):	% Intensity:	St Dev (d.n...
**Z-Average (d.nm):**	694.9	**Peak 1:**	734.6	78.3	239.5
**PdI:**	0.491	**Peak 2:**	127.4	14.0	23.58
**Intercept:**	0.957	**Peak 3:**	5250	7.7	444.8

Result quality : **Refer to quality report**

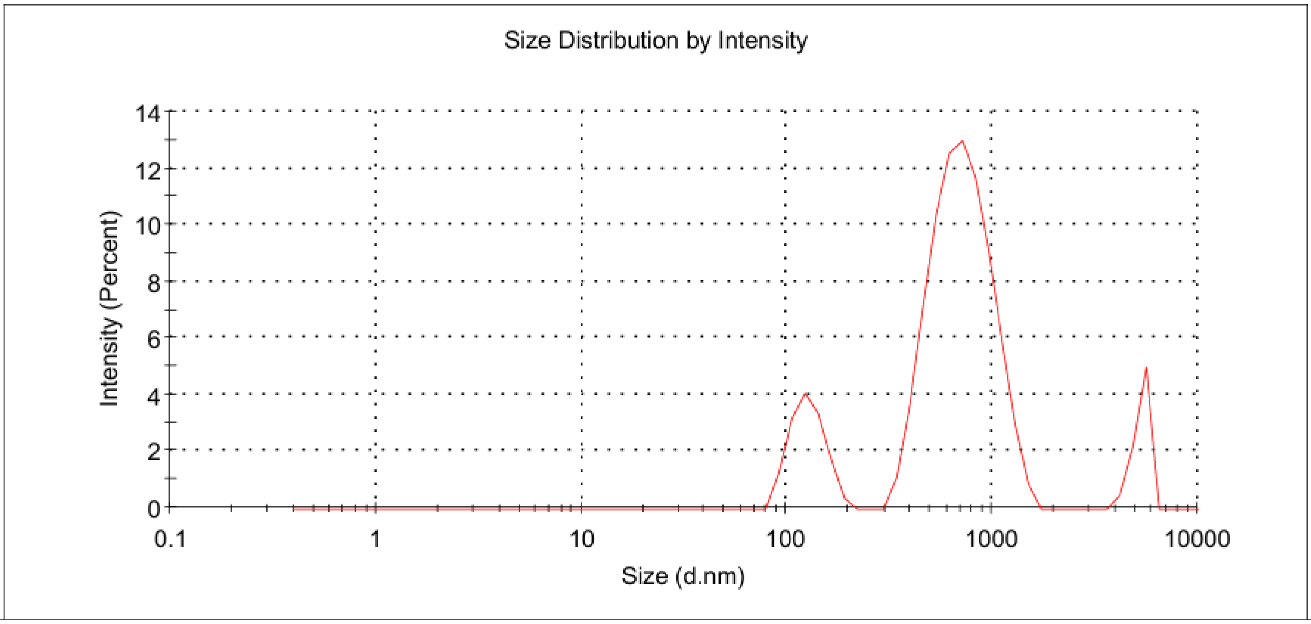

Trial 10

System

**Temperature (°C):**	25.0	**Duration Used (s):**	70
**Count Rate (kcps):**	262.6	**Measurement Position (mm):**	5.50
**Cell Description:**	Clear disposable zeta cell	**Attenuator:**	6

Results

			Size (d.nm):	% Intensity:	St Dev (d.n...
**Z-Average (d.nm):**	741.8	**Peak 1:**	734.6	82.3	199.1
**PdI:**	0.560	**Peak 2:**	109.9	17.7	20.93
**Intercept:**	0.910	**Peak 3:**	0.000	0.0	0.000

Result quality : **Refer to quality report**

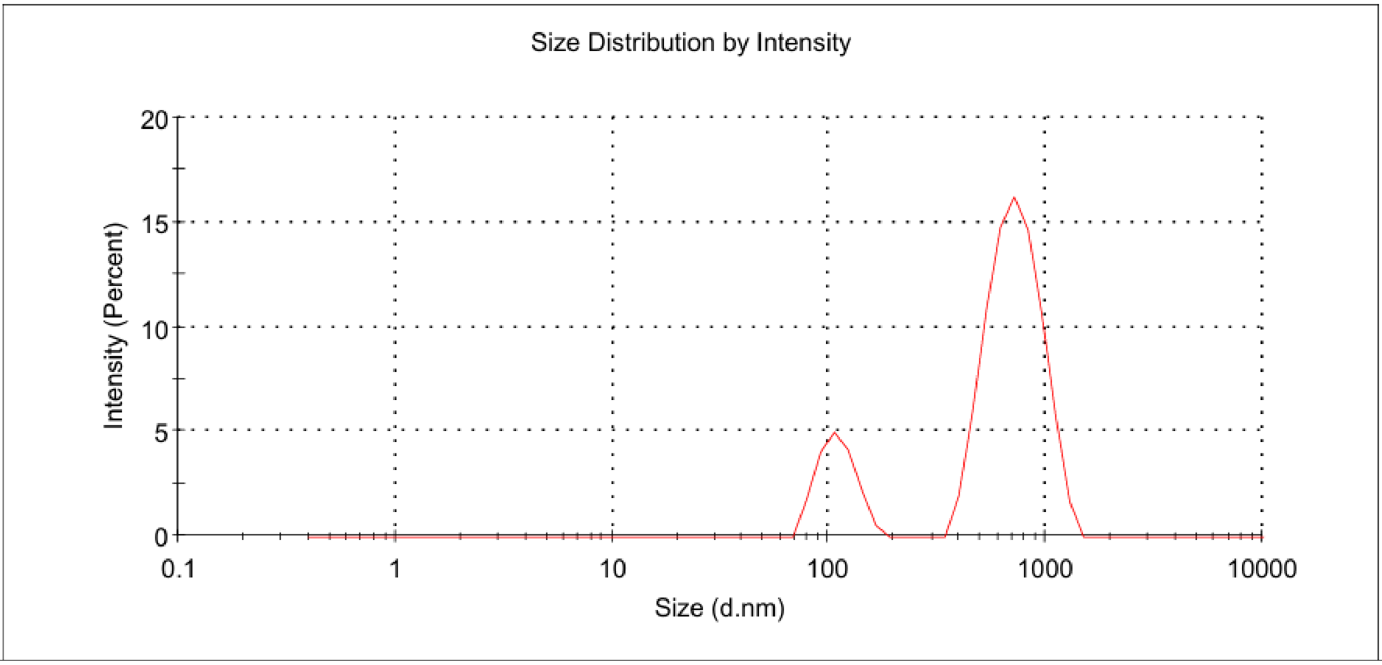

Trial 11

System

**Temperature (°C):**	25.0	**Duration Used (s):**	60
**Count Rate (kcps):**	204.8	**Measurement Position (mm):**	4.65
**Cell Description:**	Glass cuvette with square ap...	**Attenuator:**	8

Results

			Size (d.n...	% Intensity	Width (d.n...
**Z-Average (d.nm):**	374.4	**Peak 1:**	527.8	51.8	94.43
**PdI:**	0.554	**Peak 2:**	105.5	48.2	17.82
**Intercept:**	0.964	**Peak 3:**	0.000	0.0	0.000

Result quality **Refer to quality report**

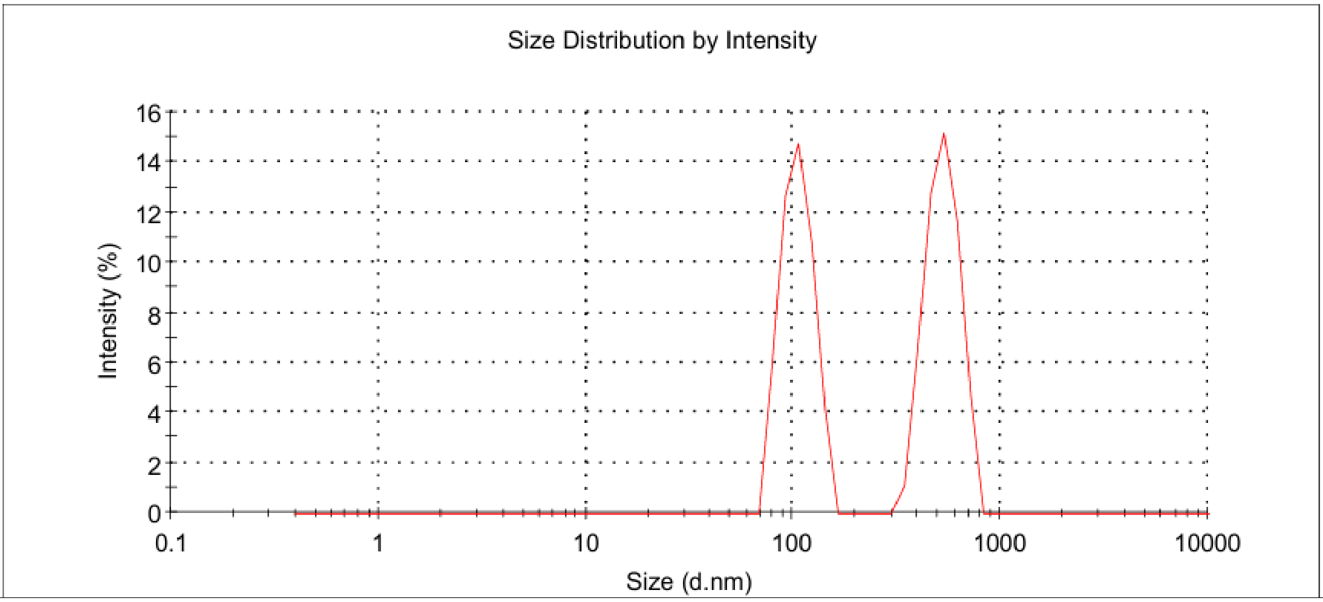

Trial 12

System

**Temperature (°C):**	25.0	**Duration Used (s):**	70
**Count Rate (kcps):**	203.2	**Measurement Position (mm):**	5.50
**Cell Description:**	Clear disposable zeta cell	**Attenuator:**	6

Results

			Size (d.nm):	% Intensity:	St Dev (d.n...
**Z-Average (d.nm):**	406.3	**Peak 1:**	281.8	71.3	55.06
**PdI:**	0.502	**Peak 2:**	87.86	28.7	15.73
**Intercept:**	0.943	**Peak 3:**	0.000	0.0	0.000

Result quality : **Refer to quality report**

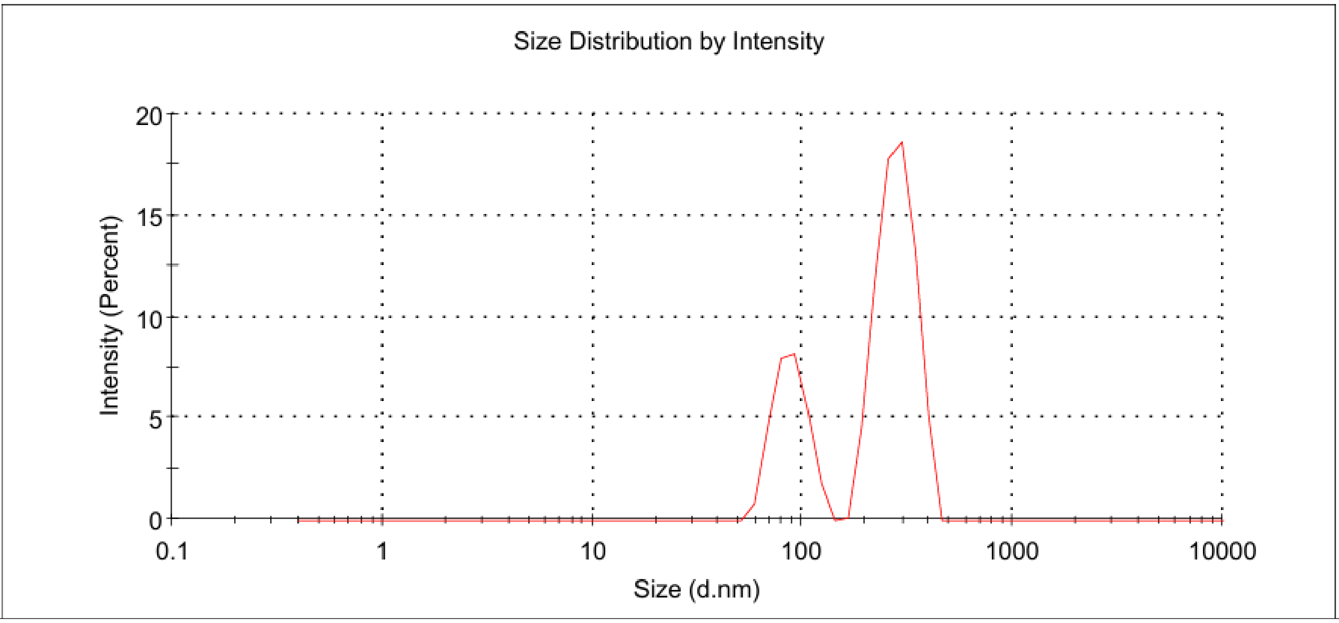

Trial 13

System

**Temperature (°C):**	25.0	**Duration Used (s):**	60
**Count Rate (kcps):**	385.3	**Measurement Position (mm):**	5.50
**Cell Description:**	Clear disposable zeta cell	**Attenuator:**	7

Results

			**Size (d.nm):**	**% Intensity:**	**St Dev (d.n...**
**Z-Average (d.nm):**	224.4	**Peak 1:**	243.9	80.5	89.73
**PdI:**	0.412	**Peak 2:**	81.53	19.5	16.77
**Intercept:**	0.921	**Peak 3:**	0.000	0.0	0.000

Result quality : Good

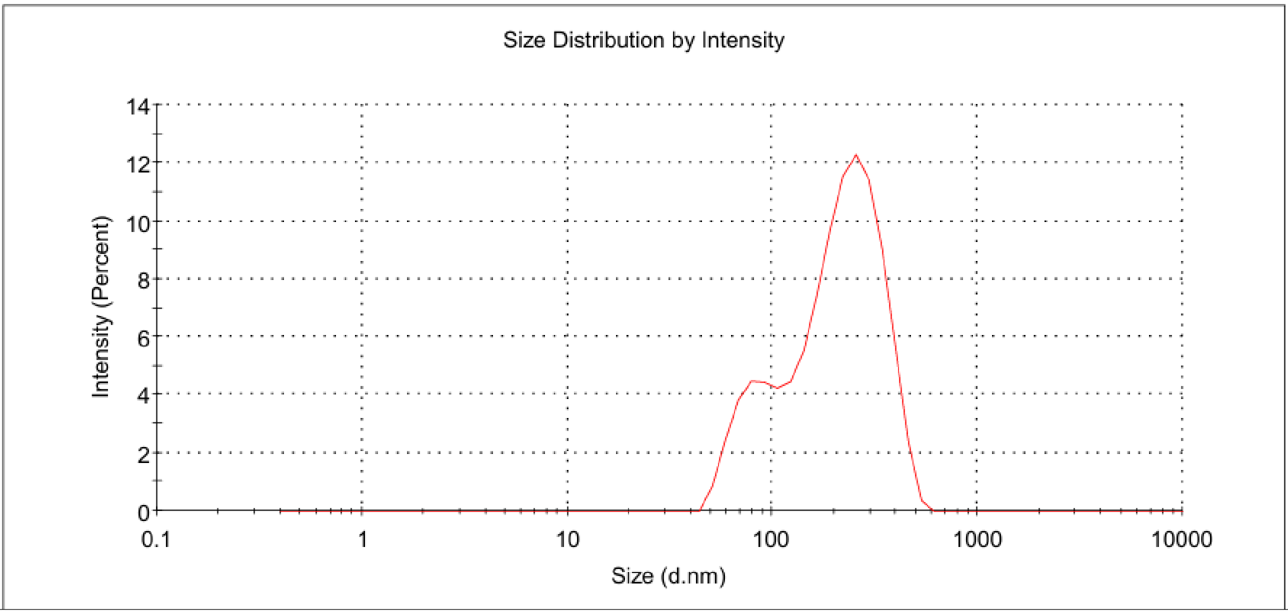

Trial 14

System

**Temperature (°C):**	25.0	**Duration Used (s):**	70
**Count Rate (kcps):**	198.3	**Measurement Position (mm):**	5.50
**Cell Description:**	Clear disposable zeta cell	**Attenuator:**	6

Results

			Size (d.nm):	% Intensity:	St Dev (d.n...
**Z-Average (d.nm):**	420.7	**Peak 1:**	462.7	59.9	110.2
**PdI:**	0.511	**Peak 2:**	102.2	40.1	24.78
**Intercept:**	0.908	**Peak 3:**	0.000	0.0	0.000

Result quality : **Refer to quality report**

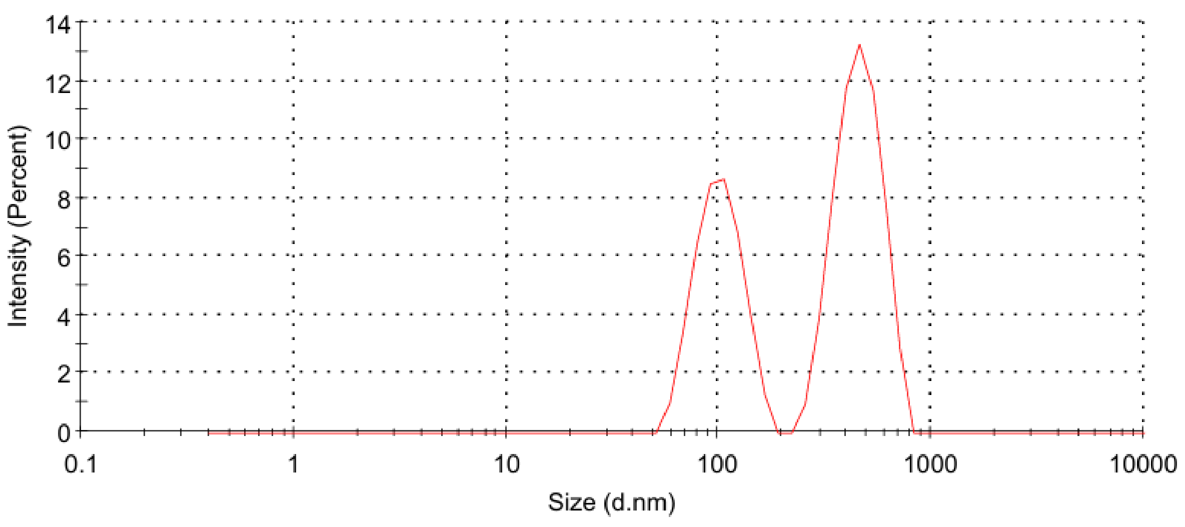

Trial 15

System

**Temperature (°C):**	25.0	**Duration Used (s):**	60
**Count Rate (kcps):**	243.3	**Measurement Position (mm):**	5.50
**Cell Description:**	Clear disposable zeta cell	**Attenuator:**	6

Results

			Size (d.nm):	% Intensity:	St Dev (d.n...
**Z-Average (d.nm):**	207.3	**Peak 1:**	316.9	57.2	102.3
**PdI:**	0.510	**Peak 2:**	101.5	42.8	30.52
**Intercept:**	0.945	**Peak 3:**	0.000	0.0	0.000

Result quality : Good

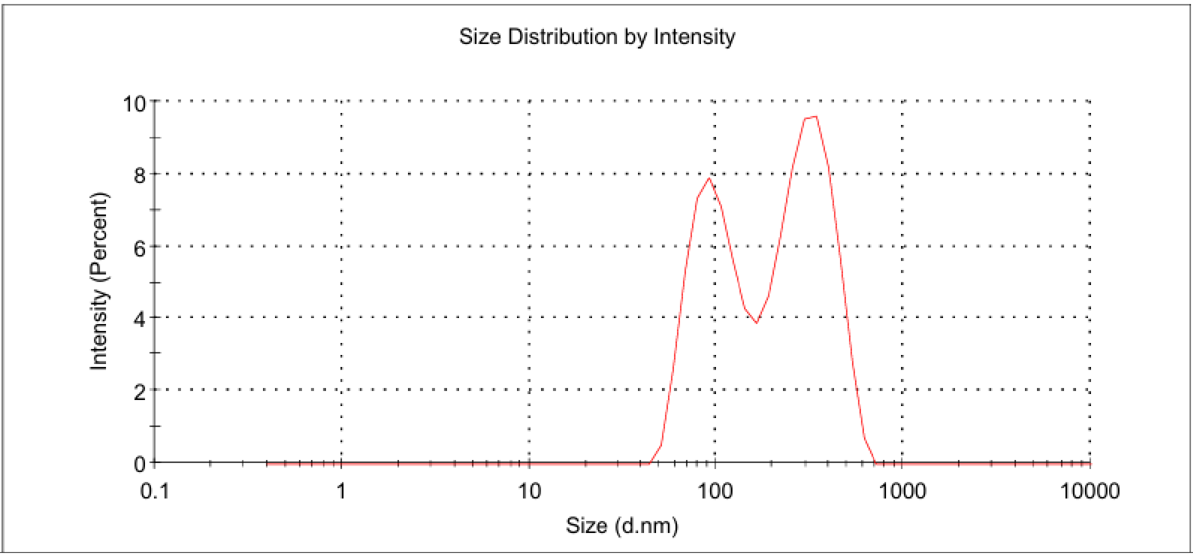

Supplement: Supplementary file 3 — Supplementary Material 3 [file 41598_2025_26860_MOESM3_ESM.pdf]
