# Supplementary material for: Development and optimization of Moxifloxacin solid lipid nanoparticles via double emulsion organic solvent free technique applying Box–Behnken experimental design
Source: Sci Rep. 2025 Nov 26;15:42013. doi: 10.1038/s41598-025-26860-x (PMC12657925; doi:10.1038/s41598-025-26860-x)

Trial 1

System

**Temperature (°C):**	25.0	**Zeta Runs:**	13
**Count Rate (kcps):**	54.7	**Measurement Position (mm):**	2.00
**Cell Description:**	Clear disposable zeta cell	**Attenuator:**	4

Results

			Mean (mV)	Area (%)	St Dev (mV)
**Zeta Potential (mV):**	-57.3	**Peak 1:**	-57.3	100.0	8.67
**Zeta Deviation (mV):**	8.67	**Peak 2:**	0.00	0.0	0.00
**Conductivity (mS/cm):**	0.00689	**Peak 3:**	0.00	0.0	0.00

Result quality : **See result quality report**

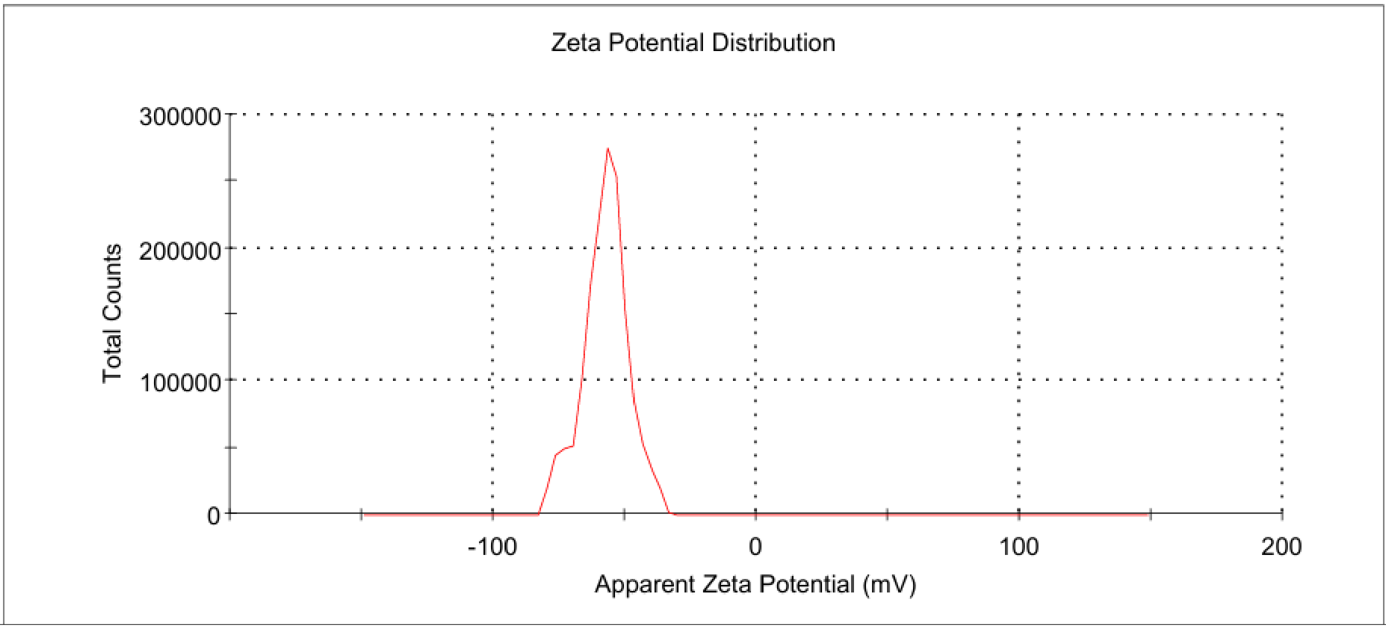

Trial 2

System

**Temperature (°C):**	24.9	**Zeta Runs:**	12
**Count Rate (kcps):**	201.8	**Measurement Position (mm):**	2.00
**Cell Description:**	Clear disposable zeta cell	**Attenuator:**	7

Results

			Mean (mV)	Area (%)	St Dev (mV)
**Zeta Potential (mV):**	-28.7	**Peak 1:**	-28.7	100.0	5.91
**Zeta Deviation (mV):**	5.91	**Peak 2:**	0.00	0.0	0.00
**Conductivity (mS/cm):**	0.0248	**Peak 3:**	0.00	0.0	0.00
**Result quality :**	Good				

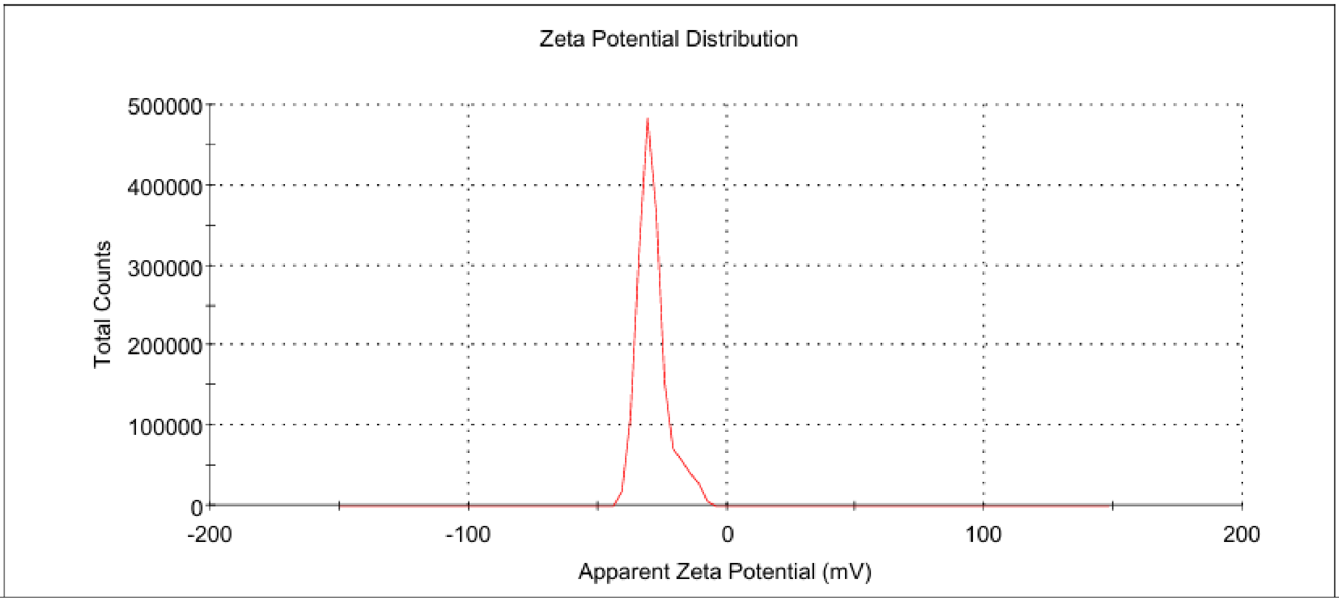

Trial 3

System

**Temperature (°C):**	25.0	**Zeta Runs:**	13
**Count Rate (kcps):**	234.9	**Measurement Position (mm):**	2.00
**Cell Description:**	Clear disposable zeta cell	**Attenuator:**	6

Results

			Mean (mV)	Area (%)	St Dev (mV)
**Zeta Potential (mV):**	-52.4	**Peak 1:**	-48.3	78.2	8.38
**Zeta Deviation (mV):**	11.7	**Peak 2:**	-69.7	21.8	4.38
**Conductivity (mS/cm):**	0.00962	**Peak 3:**	0.00	0.0	0.00
**Result quality :**	Good				

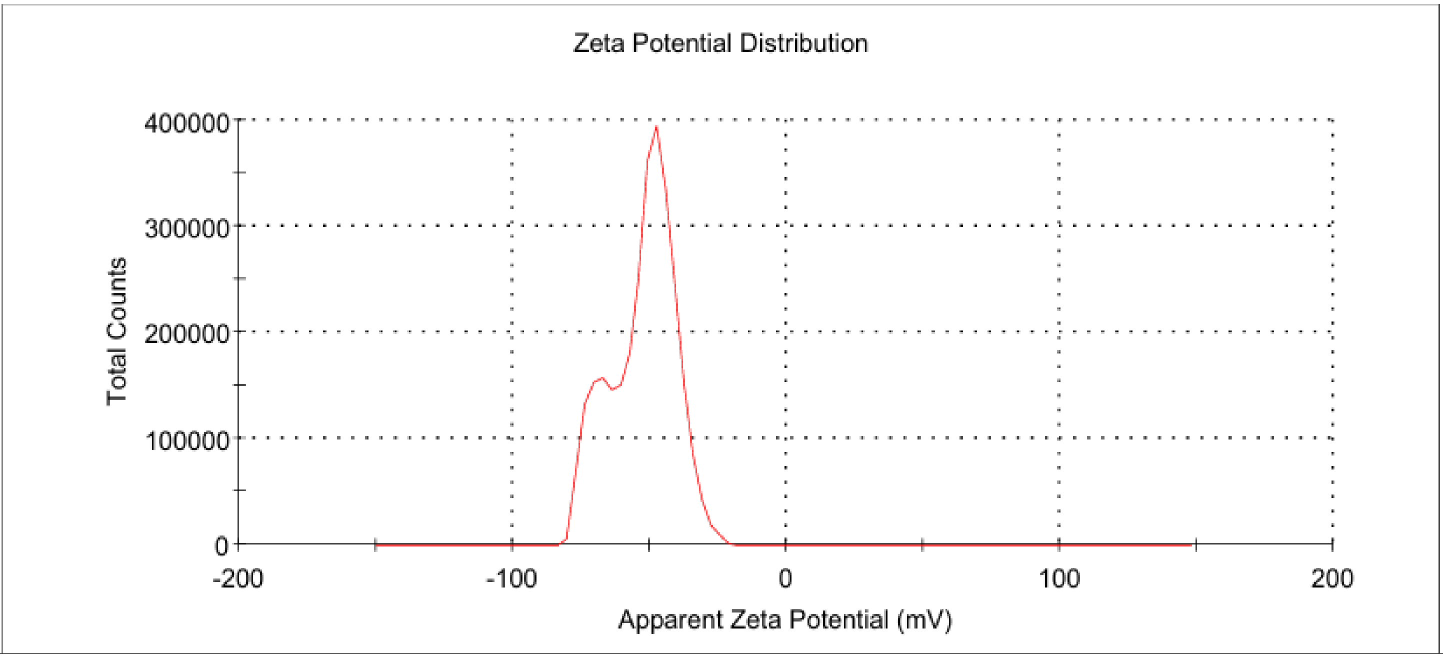

Trial 4

System

**Temperature (°C):**	24.9	**Zeta Runs:**	14
**Count Rate (kcps):**	166.7	**Measurement Position (mm):**	2.00
**Cell Description:**	Clear disposable zeta cell	**Attenuator:**	6

Results

			Mean (mV)	Area (%)	St Dev (mV)
**Zeta Potential (mV):**	-31.1	**Peak 1:**	-31.1	100.0	5.28
**Zeta Deviation (mV):**	5.28	**Peak 2:**	0.00	0.0	0.00
**Conductivity (mS/cm):**	0.00497	**Peak 3:**	0.00	0.0	0.00
**Result quality :**	Good				

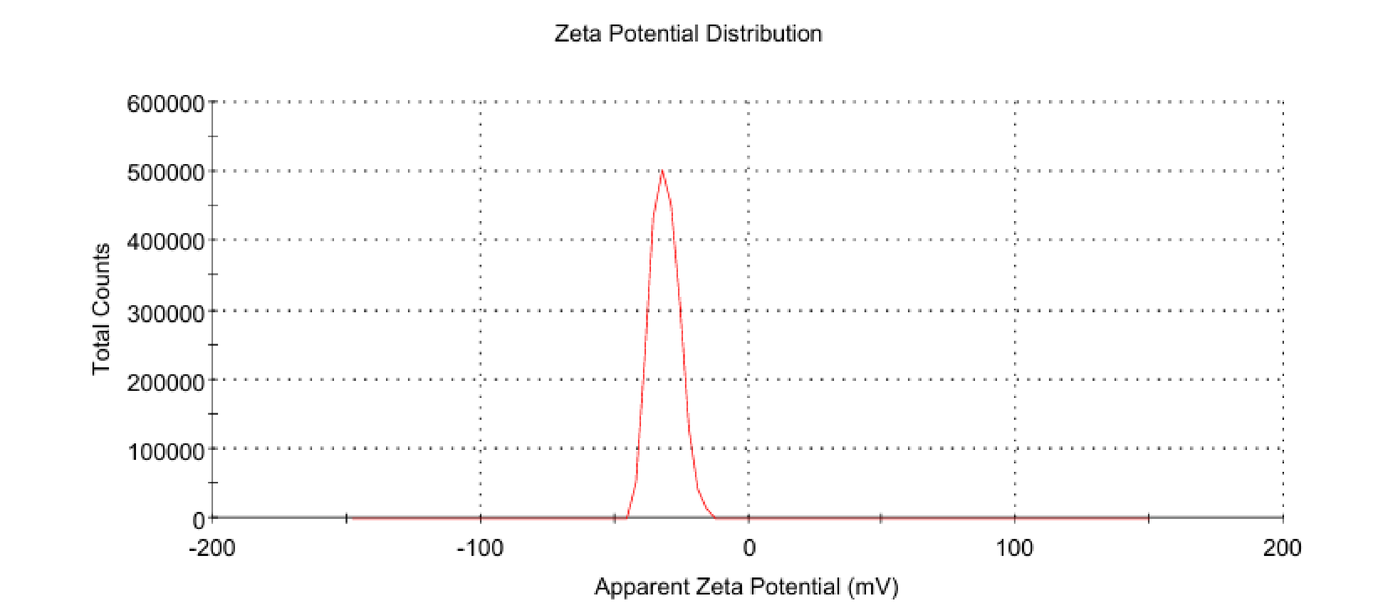

Trial 5

System

**Temperature (°C):**	25.0	**Zeta Runs:**	12
**Count Rate (kcps):**	309.3	**Measurement Position (mm):**	2.00
**Cell Description:**	Clear disposable zeta cell	**Attenuator:**	5

Results

			Mean (mV)	Area (%)	St Dev (mV)
**Zeta Potential (mV):**	-54.4	**Peak 1:**	-54.4	100.0	9.71
**Zeta Deviation (mV):**	9.71	**Peak 2:**	0.00	0.0	0.00
**Conductivity (mS/cm):**	0.0731	**Peak 3:**	0.00	0.0	0.00
**Result quality :**	Good				

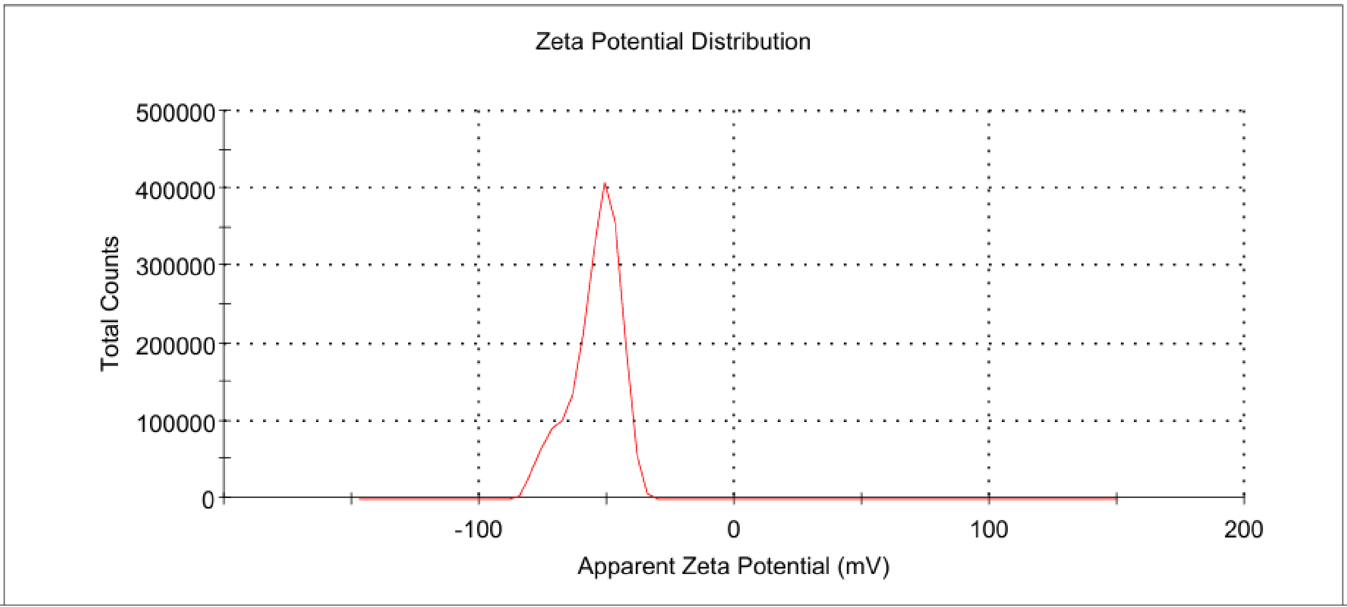

Trial 6

System

**Temperature (°C):**	24.9	**Zeta Runs:**	14
**Count Rate (kcps):**	67.3	**Measurement Position (mm):**	2.00
**Cell Description:**	Clear disposable zeta cell	**Attenuator:**	6

Results

			**Mean (mV)**	**Area (%)**	**St Dev (mV)**
**Zeta Potential (mV):**	-28.6	**Peak 1:**	-28.6	100.0	6.31
**Zeta Deviation (mV):**	6.31	**Peak 2:**	0.00	0.0	0.00
**Conductivity (mS/cm):**	0.00528	**Peak 3:**	0.00	0.0	0.00
**Result quality :**	Good				

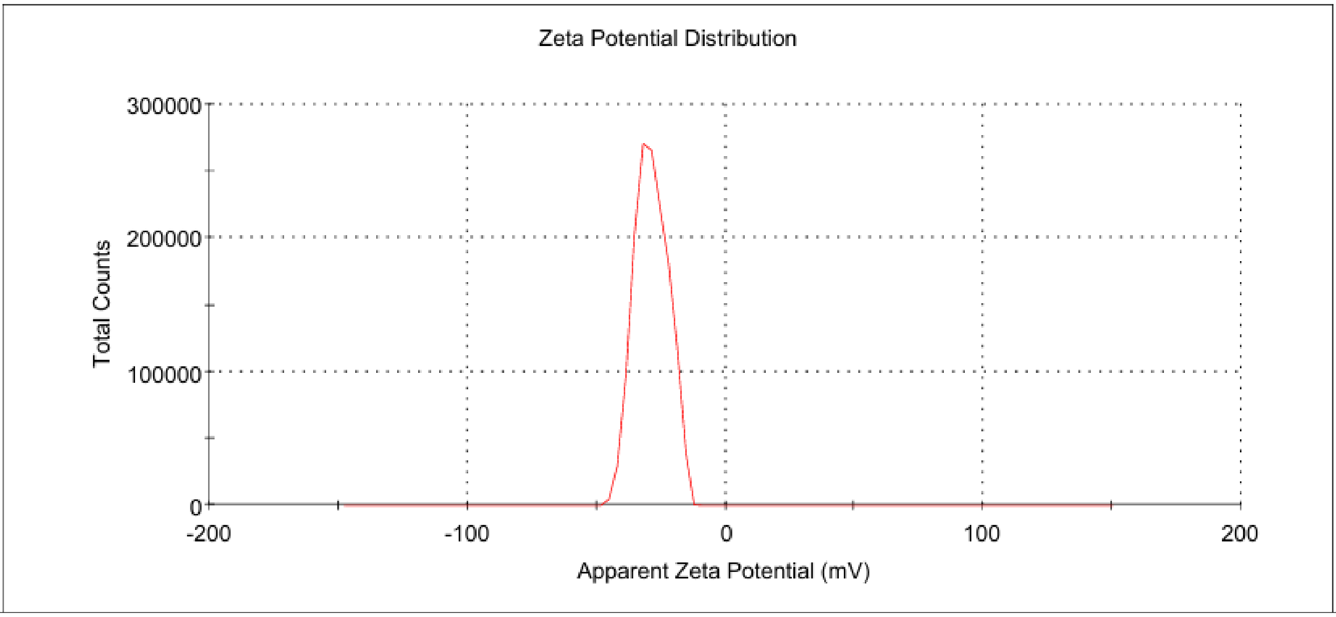

Trial 7

System

**Temperature (°C):**	25.0	**Zeta Runs:**	12
**Count Rate (kcps):**	343.7	**Measurement Position (mm):**	2.00
**Cell Description:**	Clear disposable zeta cell	**Attenuator:**	6

Results

			Mean (mV)	Area (%)	St Dev (mV)
**Zeta Potential (mV):**	-30.6	**Peak 1:**	-31.6	94.7	5.77
**Zeta Deviation (mV):**	7.28	**Peak 2:**	-10.3	5.3	3.14
**Conductivity (mS/cm):**	0.00929	**Peak 3:**	0.00	0.0	0.00
**Result quality :**	Good				

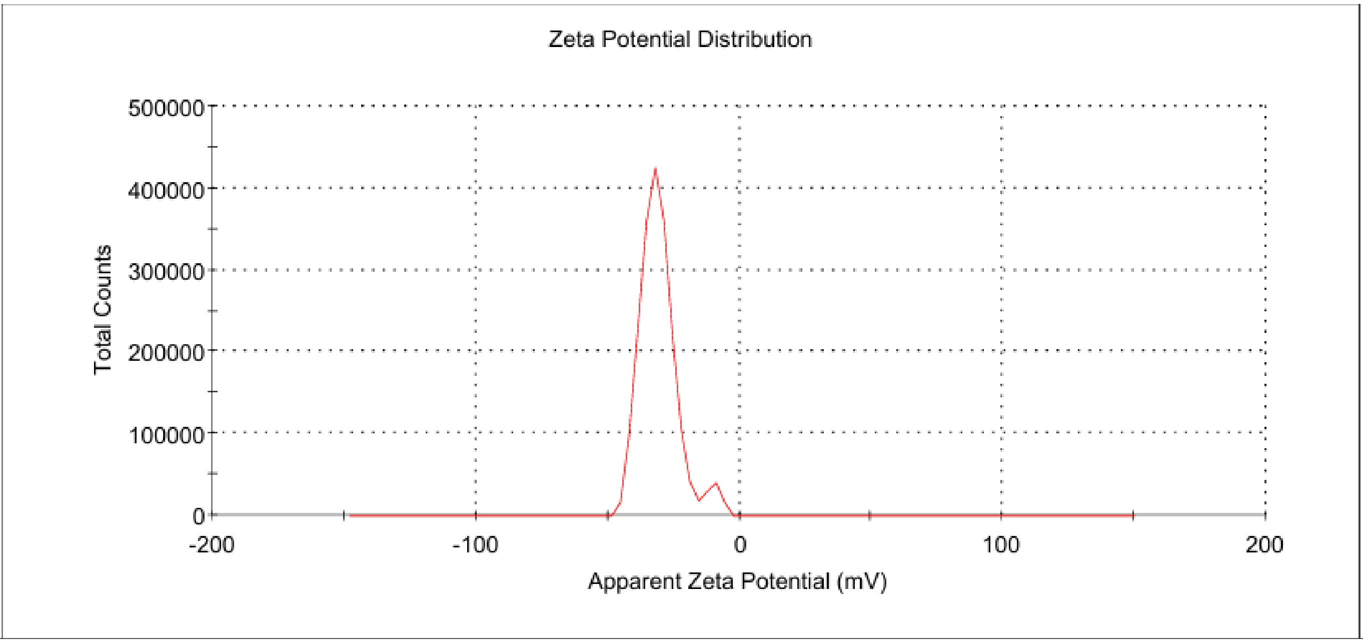

Trial 8

System

**Temperature (°C):**	25.0	**Zeta Runs:**	12
**Count Rate (kcps):**	129.0	**Measurement Position (mm):**	2.00
**Cell Description:**	Clear disposable zeta cell	**Attenuator:**	4

Results

			Mean (mV)	Area (%)	St Dev (mV)
**Zeta Potential (mV):**	-58.1	**Peak 1:**	-58.2	100.0	9.99
**Zeta Deviation (mV):**	11.5	**Peak 2:**	0.00	0.0	0.00
**Conductivity (mS/cm):**	0.0153	**Peak 3:**	0.00	0.0	0.00
**Result quality :**	Good				

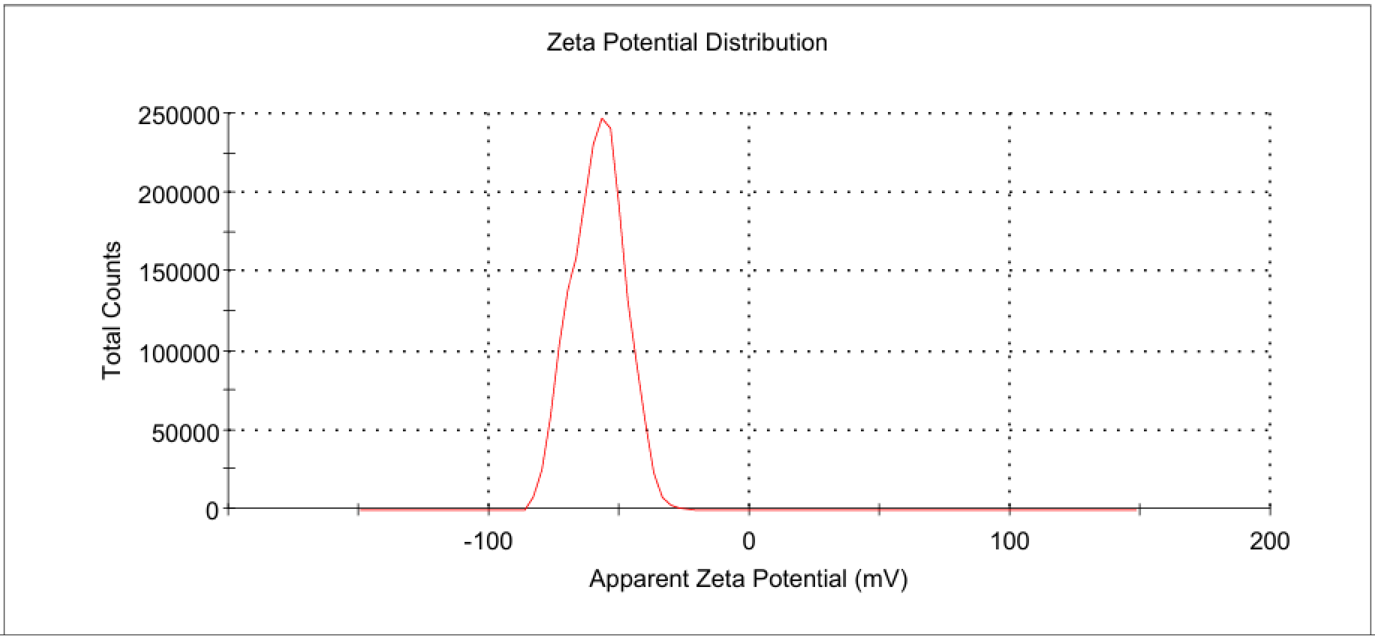

Trial 9

System

**Temperature (°C):**	25.0	**Zeta Runs:**	12
**Count Rate (kcps):**	100.0	**Measurement Position (mm):**	2.00
**Cell Description:**	Clear disposable zeta cell	**Attenuator:**	5

Results

			Mean (mV)	Area (%)	St Dev (mV)
**Zeta Potential (mV):**	-42.6	**Peak 1:**	-45.8	77.0	3.93
**Zeta Deviation (mV):**	7.40	**Peak 2:**	-31.1	23.0	3.94
**Conductivity (mS/cm):**	0.0376	**Peak 3:**	0.00	0.0	0.00
**Result quality :**	Good				

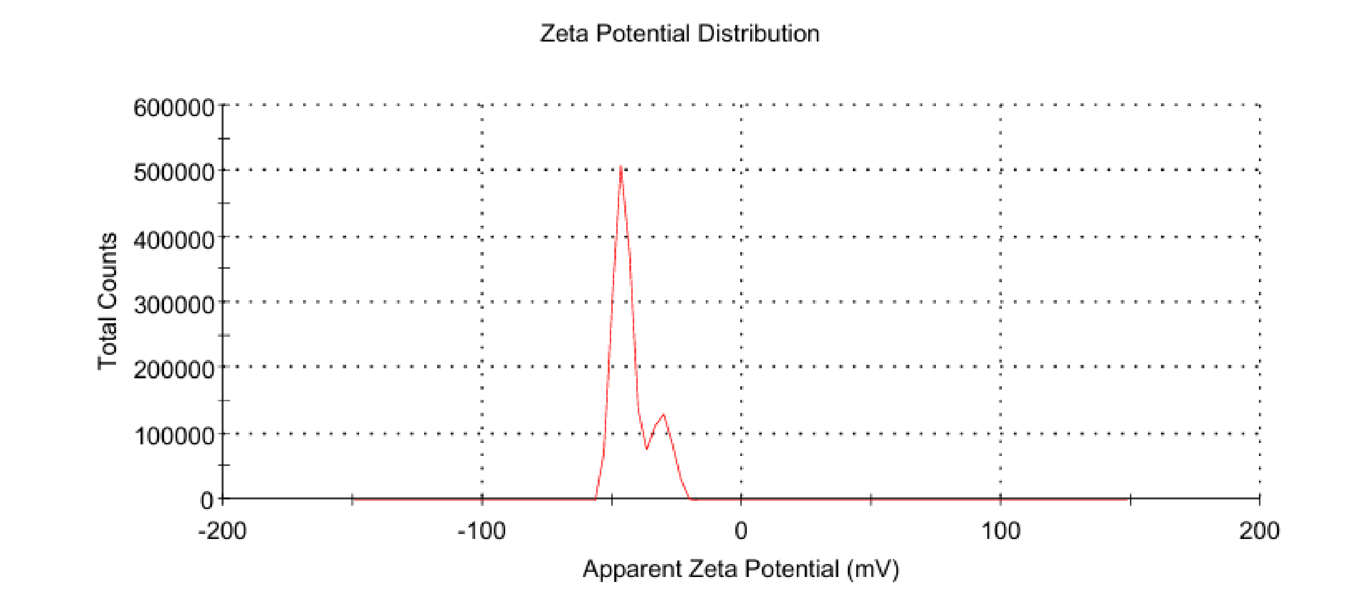

Trial 10

System

**Temperature (°C):**	25.0	**Zeta Runs:**	14
**Count Rate (kcps):**	324.7	**Measurement Position (mm):**	2.00
**Cell Description:**	Clear disposable zeta cell	**Attenuator:**	5

Results

			Mean (mV)	Area (%)	St Dev (mV)
**Zeta Potential (mV):**	-32.9	**Peak 1:**	-36.0	85.1	7.64
**Zeta Deviation (mV):**	12.3	**Peak 2:**	-15.8	12.1	3.30
**Conductivity (mS/cm):**	0.00512	**Peak 3:**	20.7	1.7	2.43

Result quality : **See result quality report**

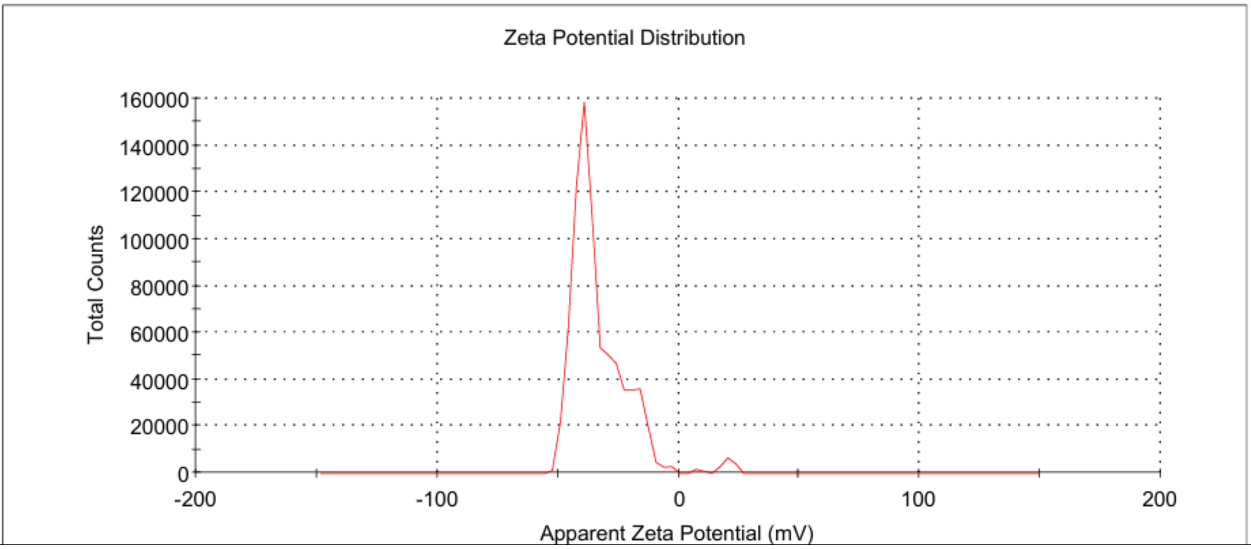

Trial 11

System

**Temperature (°C):**	25.0	**Zeta Runs:**	12
**Count Rate (kcps):**	78.1	**Measurement Position (mm):**	2.00
**Cell Description:**	Clear disposable zeta cell	**Attenuator:**	6

Results

			Mean (mV)	Area (%)	St Dev (mV)
**Zeta Potential (mV):**	-37.4	**Peak 1:**	-37.4	100.0	6.32
**Zeta Deviation (mV):**	6.32	**Peak 2:**	0.00	0.0	0.00
**Conductivity (mS/cm):**	0.00739	**Peak 3:**	0.00	0.0	0.00
**Result quality :**	Good				

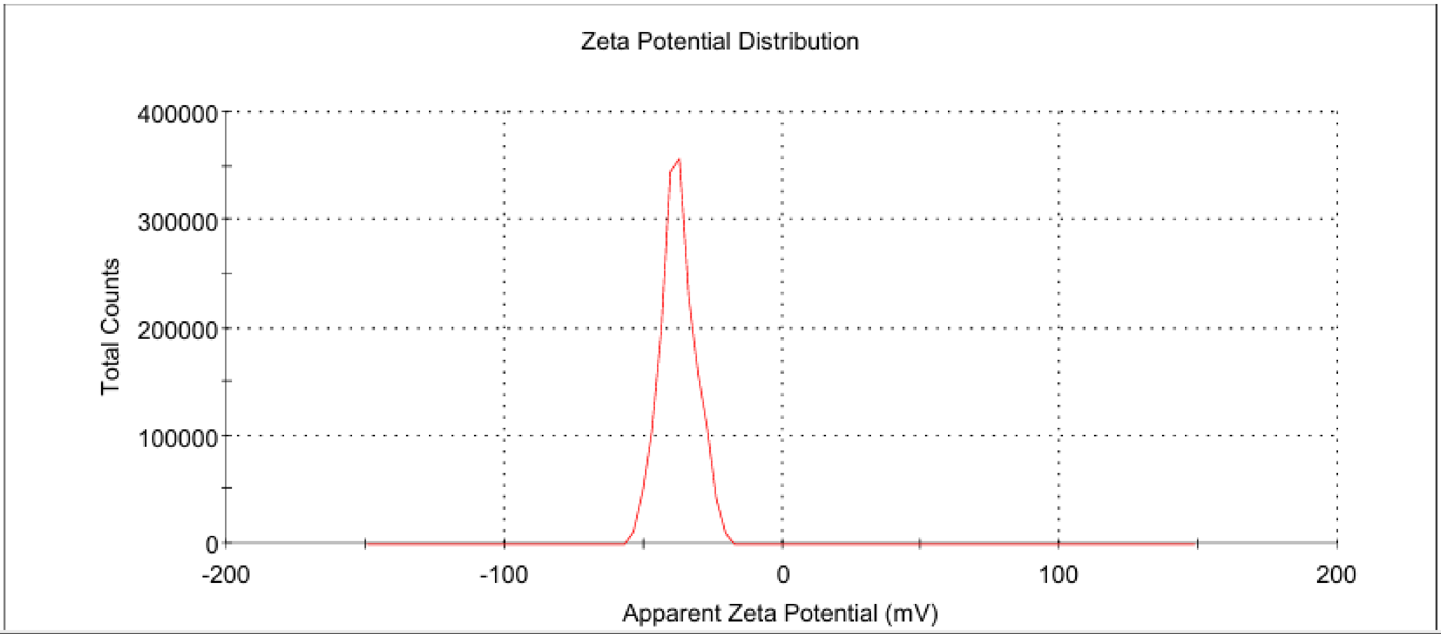

Trial 12

System

**Temperature (°C):**	25.0	**Zeta Runs:**	14
**Count Rate (kcps):**	124.6	**Measurement Position (mm):**	2.00
**Cell Description:**	Clear disposable zeta cell	**Attenuator:**	6

Results

			Mean (mV)	Area (%)	St Dev (mV)
**Zeta Potential (mV):**	-49.2	**Peak 1:**	-49.2	100.0	8.99
**Zeta Deviation (mV):**	8.99	**Peak 2:**	0.00	0.0	0.00
**Conductivity (mS/cm):**	0.0263	**Peak 3:**	0.00	0.0	0.00

Result quality : **See result quality report**

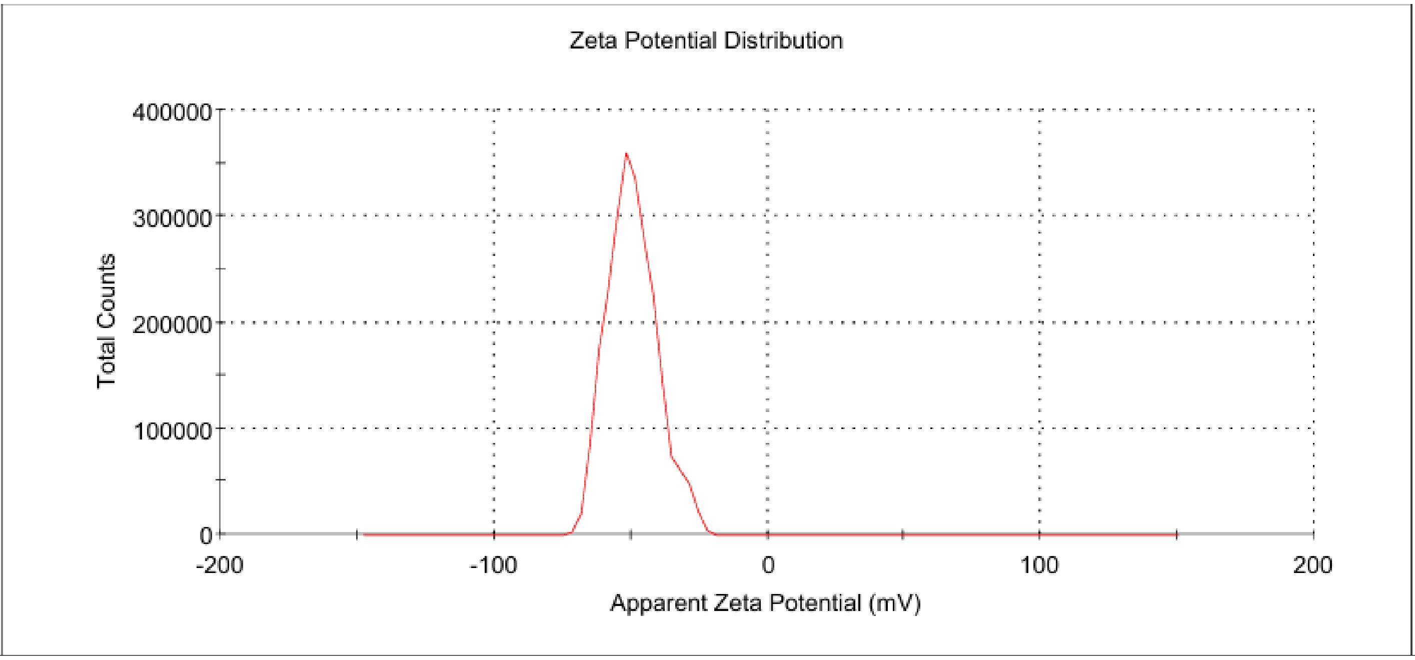

Trial 13

System

**Temperature (°C):**	25.0	**Zeta Runs:**	13
**Count Rate (kcps):**	209.6	**Measurement Position (mm):**	2.00
**Cell Description:**	Clear disposable zeta cell	**Attenuator:**	5

Results

			Mean (mV)	Area (%)	St Dev (mV)
**Zeta Potential (mV):**	-47.4	**Peak 1:**	-52.4	67.4	8.39
**Zeta Deviation (mV):**	11.5	**Peak 2:**	-35.6	31.9	5.52
**Conductivity (mS/cm):**	0.0132	**Peak 3:**	-12.5	0.7	2.15
**Result quality :**	**See result quality report**				

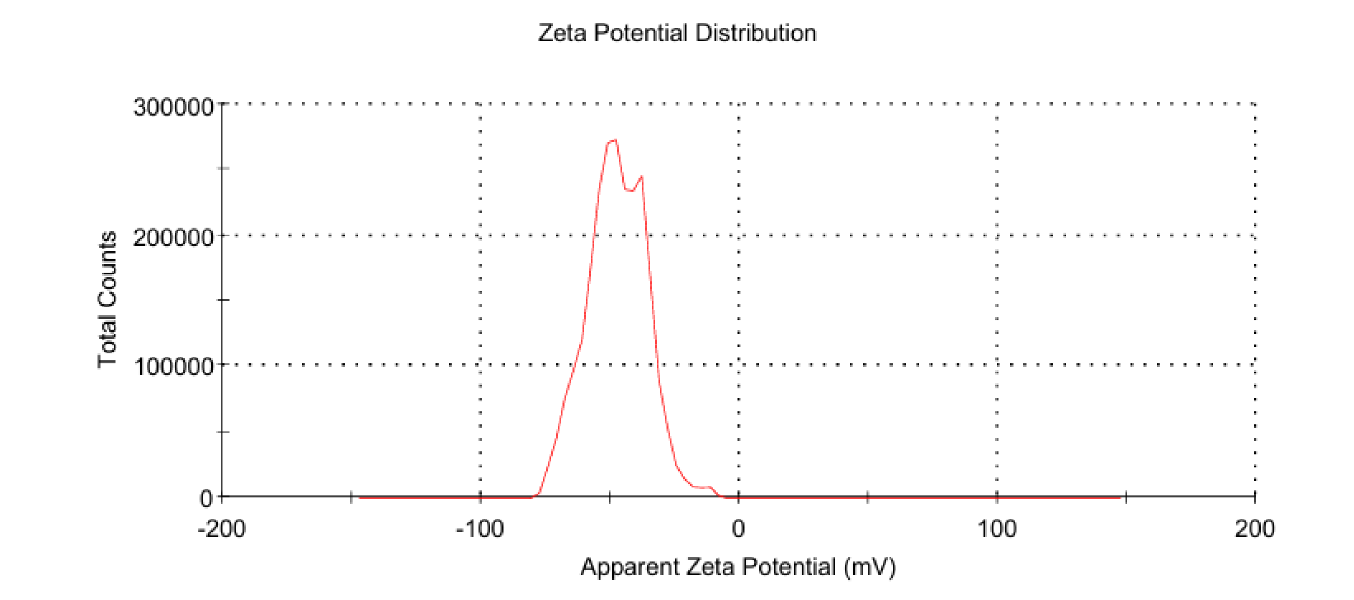

Trial 14

System

**Temperature (°C):**	25.1	**Zeta Runs:**	14
**Count Rate (kcps):**	107.7	**Measurement Position (mm):**	2.00
**Cell Description:**	Clear disposable zeta cell	**Attenuator:**	6

Results

			Mean (mV)	Area (%)	St Dev (mV)
**Zeta Potential (mV):**	-45.2	**Peak 1:**	-45.2	100.0	9.87
**Zeta Deviation (mV):**	9.87	**Peak 2:**	0.00	0.0	0.00
**Conductivity (mS/cm):**	0.0123	**Peak 3:**	0.00	0.0	0.00
**Result quality :**	Good				

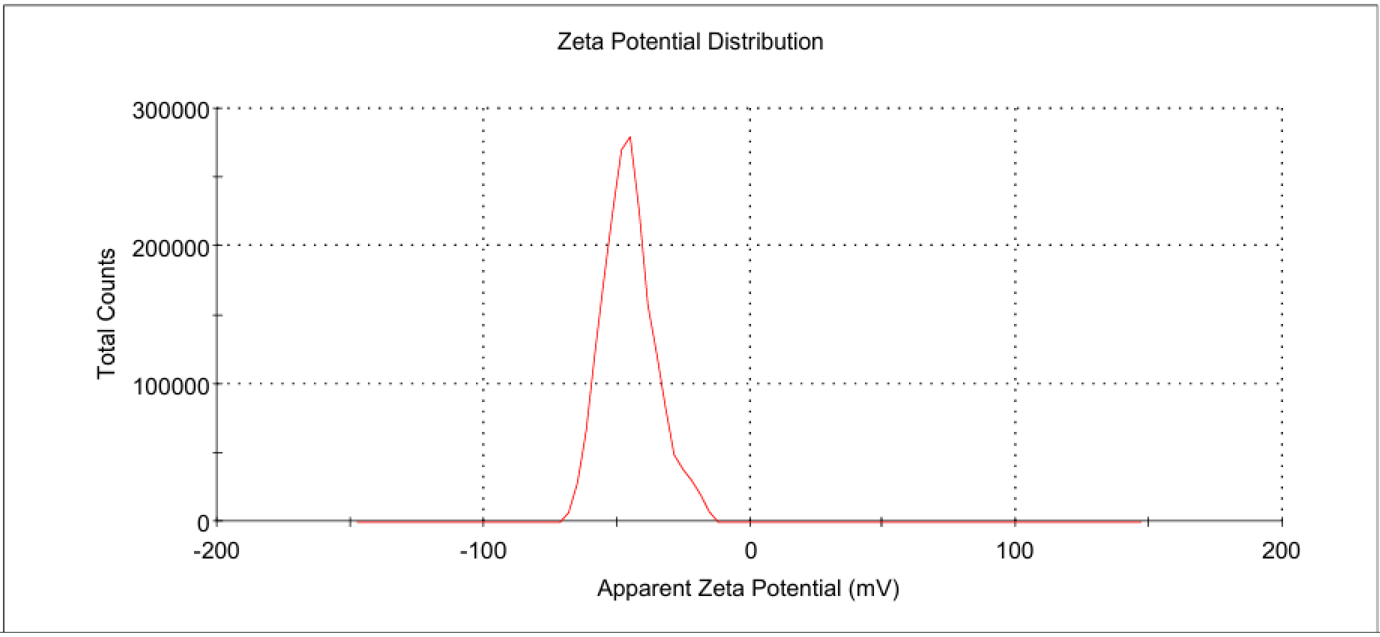

Trial 15

System

**Temperature (°C):**	25.1	**Zeta Runs:**	12
**Count Rate (kcps):**	316.2	**Measurement Position (mm):**	2.00
**Cell Description:**	Clear disposable zeta cell	**Attenuator:**	6

Results

			Mean (mV)	Area (%)	St Dev (mV)
**Zeta Potential (mV):**	-38.0	**Peak 1:**	-38.0	100.0	7.22
**Zeta Deviation (mV):**	7.22	**Peak 2:**	0.00	0.0	0.00
**Conductivity (mS/cm):**	0.00747	**Peak 3:**	0.00	0.0	0.00
**Result quality :**	Good				

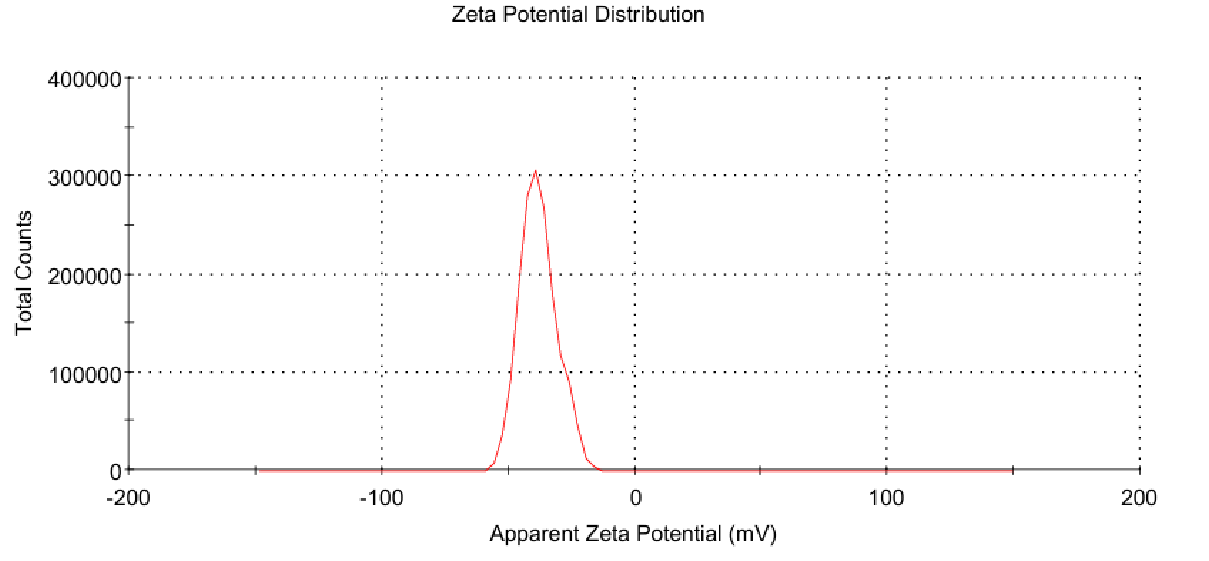

Supplement: Supplementary file 4 — Supplementary Material 4 [file 41598_2025_26860_MOESM4_ESM.pdf]
