# Supplementary material for: Development and optimization of Moxifloxacin solid lipid nanoparticles via double emulsion organic solvent free technique applying Box–Behnken experimental design
Source: Sci Rep. 2025 Nov 26;15:42013. doi: 10.1038/s41598-025-26860-x (PMC12657925; doi:10.1038/s41598-025-26860-x)
Supplement: Supplementary file 5 — Supplementary Material 5 [file 41598_2025_26860_MOESM5_ESM.pdf]

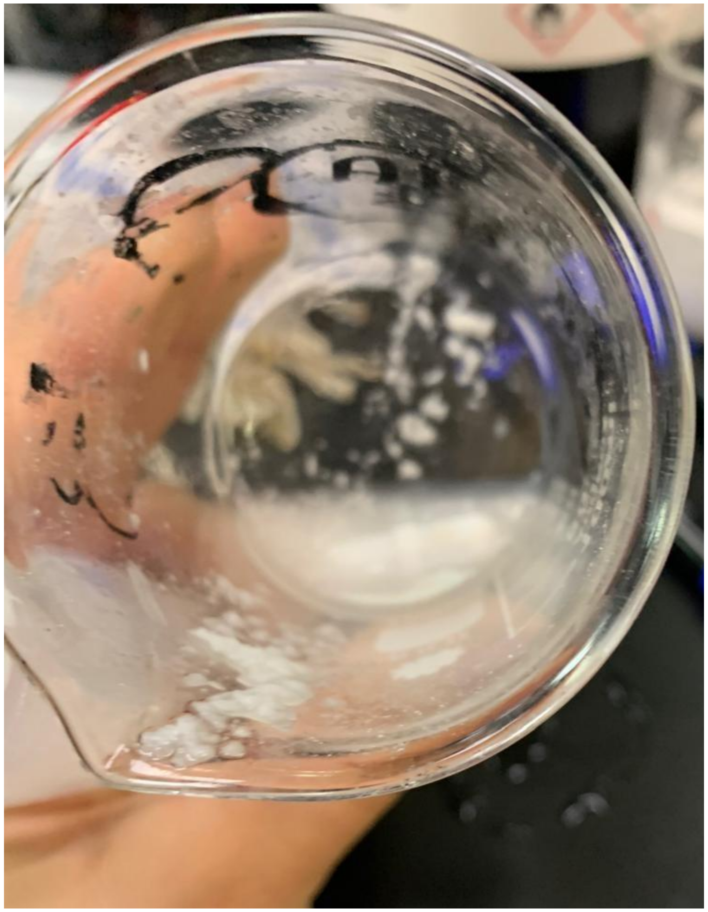

This image corresponds to the clumps formed when using the two types of lecithin

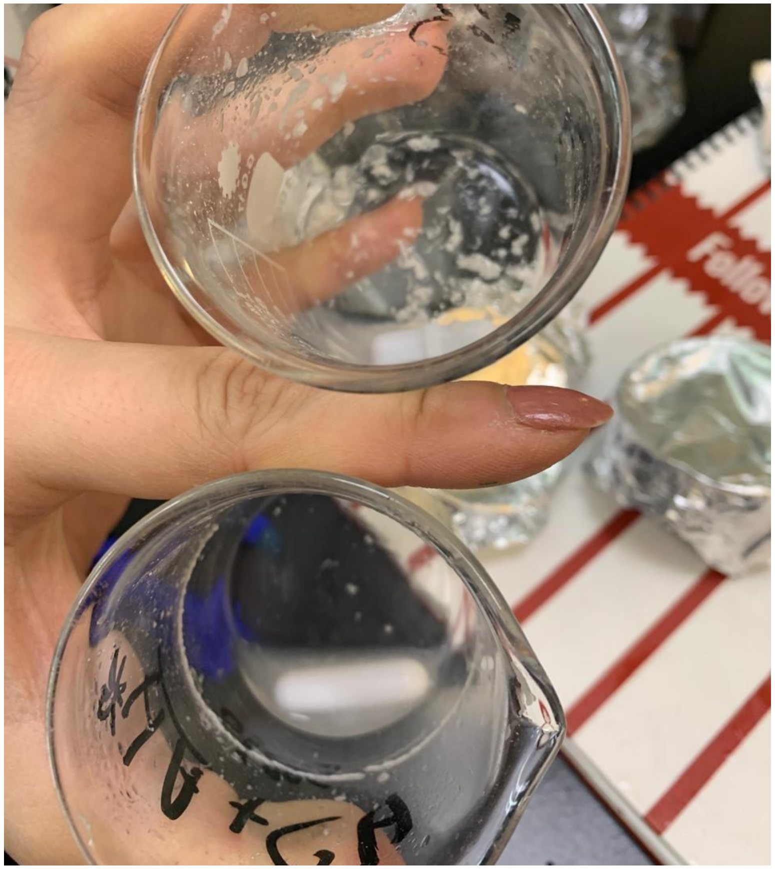

This image shows the comparison between Span 80 and lecthins indicating the difference in the emulsion formation.

as shown Span 80 resulted in a stable emulsion, while the alternative lecithins produced clumps.

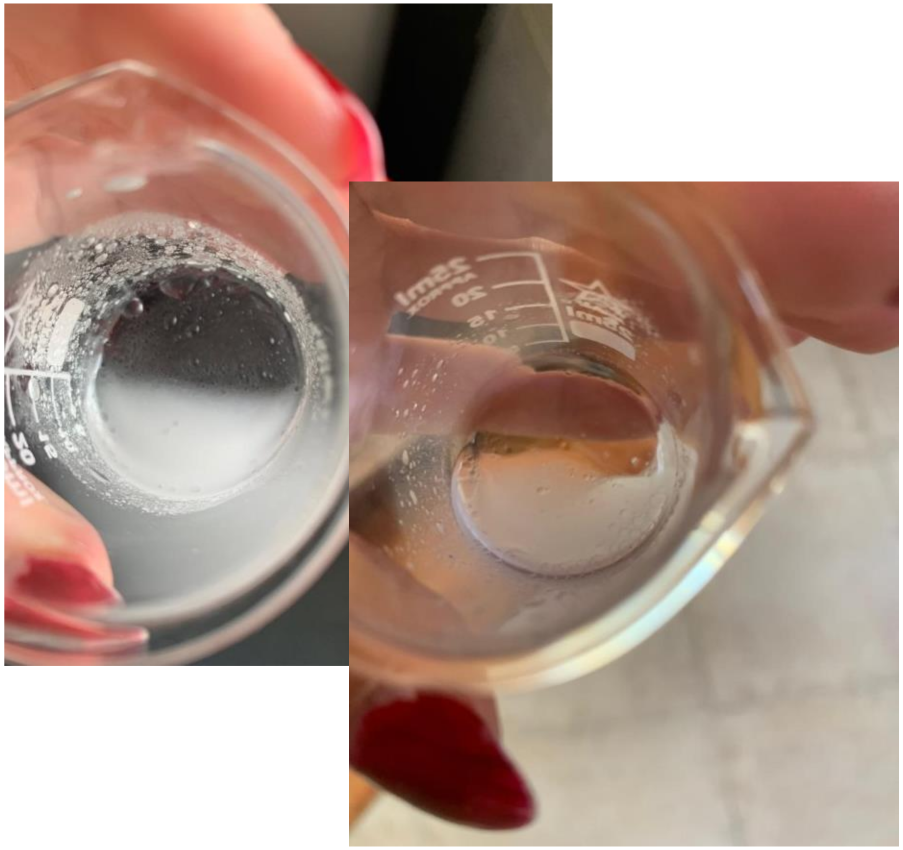

This images corresponds to the Poloxamer 80
Phase-separated emulsion
