# Supplementary material for: Development and optimization of Moxifloxacin solid lipid nanoparticles via double emulsion organic solvent free technique applying Box–Behnken experimental design
Source: Sci Rep. 2025 Nov 26;15:42013. doi: 10.1038/s41598-025-26860-x (PMC12657925; doi:10.1038/s41598-025-26860-x)
Supplement: Supplementary file 6 — Supplementary Material 6 [file 41598_2025_26860_MOESM6_ESM.pdf]

Development and optimization of Moxifloxacin solid lipid nanoparticles via double emulsion organic solvent free technique applying Box-Behnken experimental design

Esraa M.Elshazly[1], Mona G. Arafa[1,3,4*], Samia A. Nour[2]

[1]Department of Pharmaceutics and Pharmaceutical Technology, Faculty of Pharmacy, The British University in Egypt, Cairo, Egypt

[2]Department of Pharmaceutics and Industrial Pharmacy, Faculty of Pharmacy, Cairo University, Cairo, Egypt

[3]Chemotherapeutic Unit, Mansoura University Hospitals, Mansoura, Egypt

[4]Nanotechnology Research Center, The British University in Egypt, Cairo, Egypt

* Corresponding author: Mona G. Arafa (mona.arafa@bue.edu.eg)

1-Calibration curve construction

A stock solution of 100 µg/ml MOX in distilled water was prepared in a 100 ml volumetric flask. Serial dilutions of the stock solution were made in 10 ml volumetric flasks to achieve concentrations of 1, 2, 3, 4, 5, 6, 7, 8, 9, 10, 11, 12 and 13 µg/ml using the UV/ Visible Spectrophotometer the absorption of the solutions was determined at the previously determined (λ_{max})[15]. Mean absorbance of three replicated readings was plotted against corresponding concentrations on the Y and X-axes, respectively, to obtain a calibration curve. Linear regression analysis was performed using Excel Microsoft® software to display the equation of the best-fit line. The procedural constant (K) was then determined as the inverse of the slope obtained from the equation.

Calibration curve results

The UV absorbance of MOX at the predetermined λ_{max} of 293 nm was measured using solutions of serial dilutions prepared in distilled water. The mean values of three independent measurements were calculated and used to construct the calibration curve presented in Figure S1. The regression equation and the coefficient of determination (R^2) (0.9973) are displayed on the graph of Fig S1, this indicating good linearity of the MOX concentration absorbance in accordance with Beer_ Lambert's law. The procedural constant (K) was determined and was found to be 10.

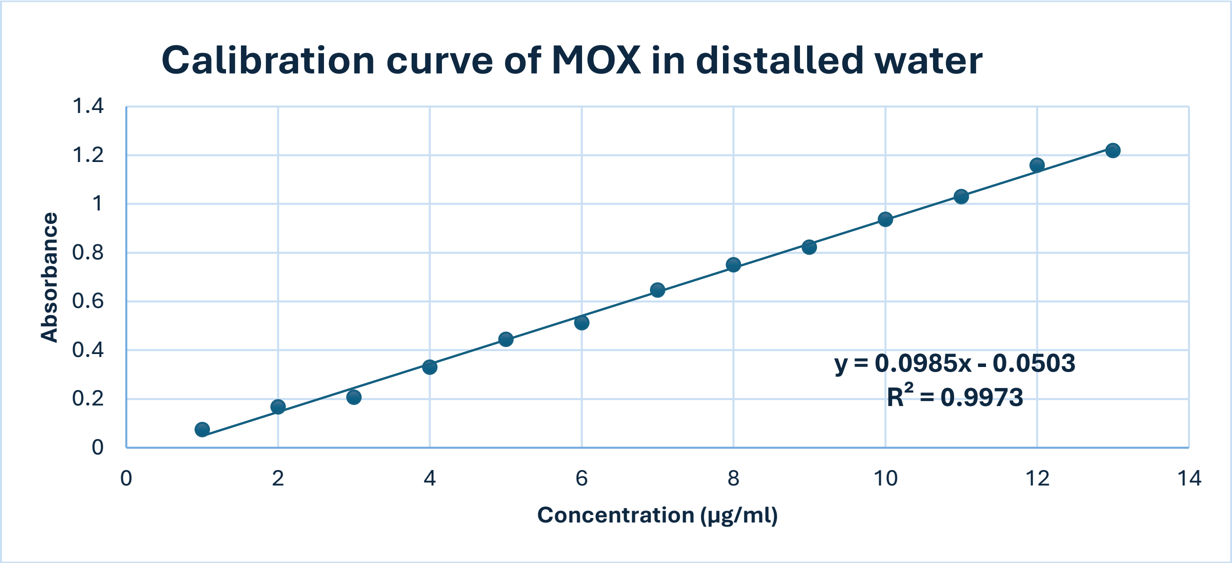

Figure S1: MOX calibration curve in distilled water at λ_{max} 293 nm

Table S1: Raw data of Entrapment Efficiency %

Trial	Raw1	Raw2	Raw3	Mean	SD	±SD
1	88.3	84.22	91.78	88.1	3.8	88.10 ± 3.78
2	80.24	89.29	70.89	80.14	9.2	80.14 ± 9.20
3	90.39	99.69	80.96	90.34667	9.4	90.35 ± 9.36
4	91.88	97.26	86.73	91.95667	5.3	91.96 ± 5.27
5	88.49	77.46	99.13	88.36	10.8	88.36 ± 10.83
6	89.45	83.7	95.95	89.7	6.1	89.70 ± 6.12
7	70.6	56.54	84.43	70.52333	13.9	70.52 ± 13.94
8	75.47	82.22	67.87	75.18667	7.2	75.19 ± 7.17
9	71.35	76.7	65.28	71.11	5.7	71.11 ± 5.72
10	87.96	91.48	82.49	87.31	4.5	87.31 ± 4.53
11	78.25	76.8	57.44	70.83	11.6	70.83 ± 11.62
12	74.27	63.63	84.22	74.04	10.3	74.04 ± 10.30
13	79.1	68.94	89.2	79.08	10.1	79.08 ± 10.12
14	62.24	65.63	59.84	62.57	2.9	62.57 ± 2.91
15	81.27	87.74	74.32	81.11	6.7	81.11 ± 6.70

X-ray diffraction (XRD)

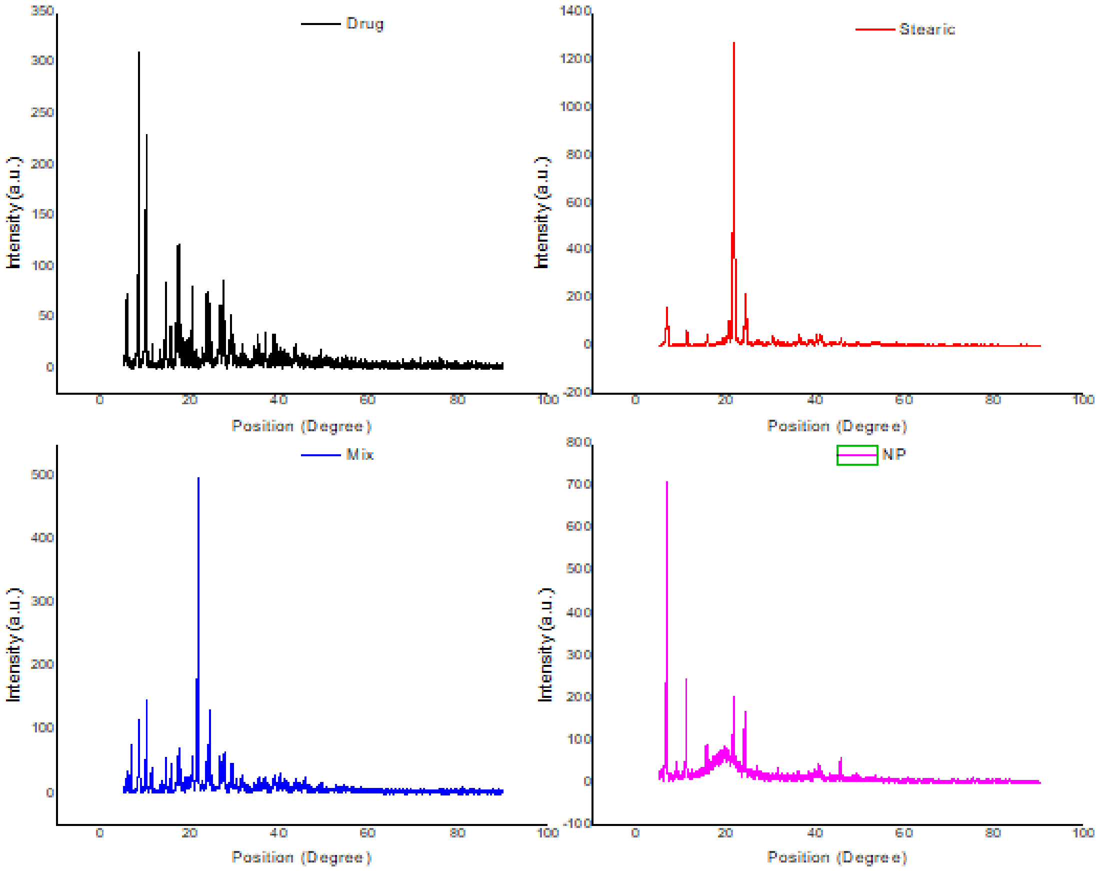

Figure S2: XRD of pure drug, stearic acid, physical mixture of drug: stearic 1:1 and F-opt

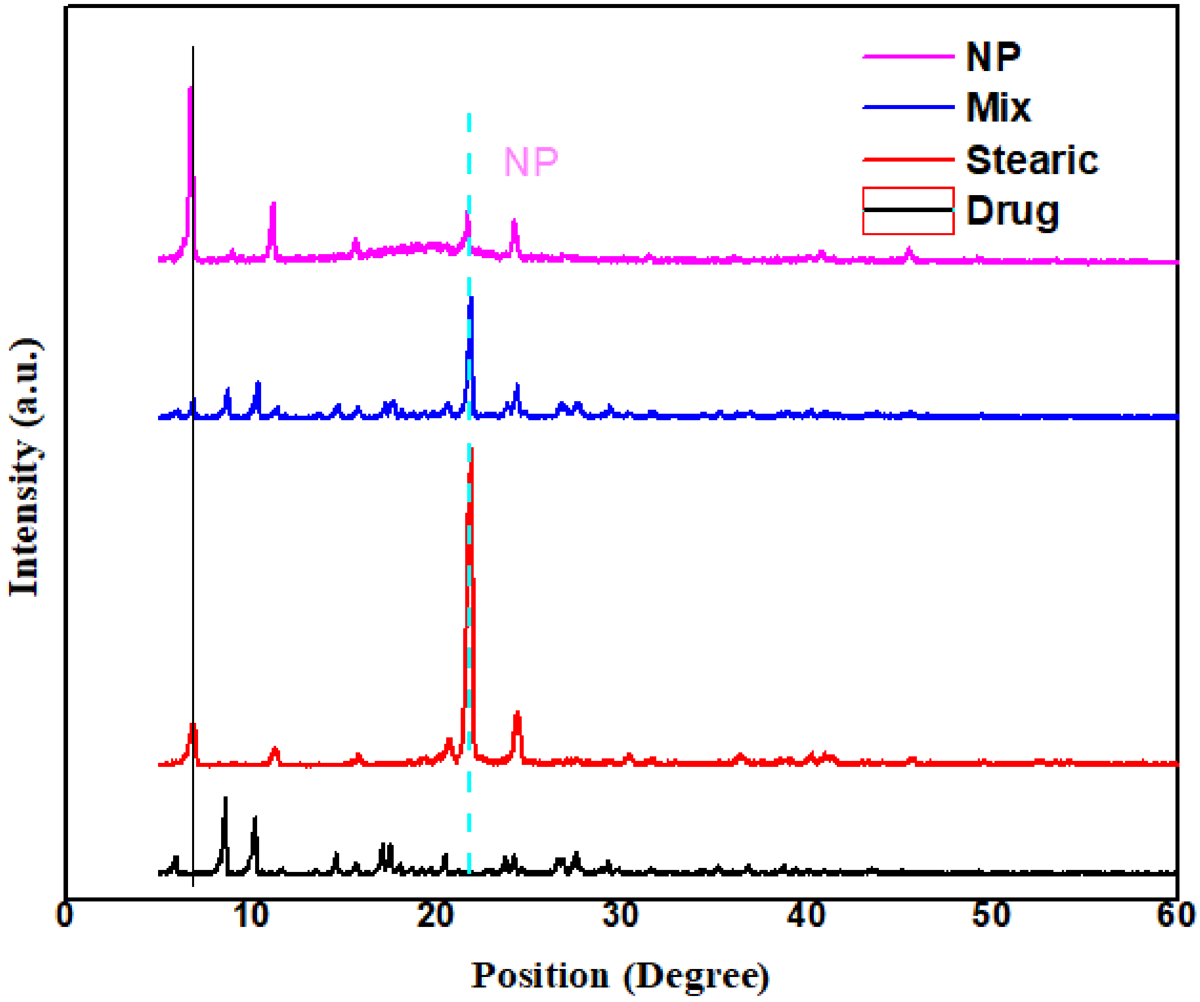

Figure S3: XRD stacked lines for pure drug, stearic acid, physical mixture of drug: stearic 1:1 and F-opt

Fourier transform infrared (FTIR) spectroscopy

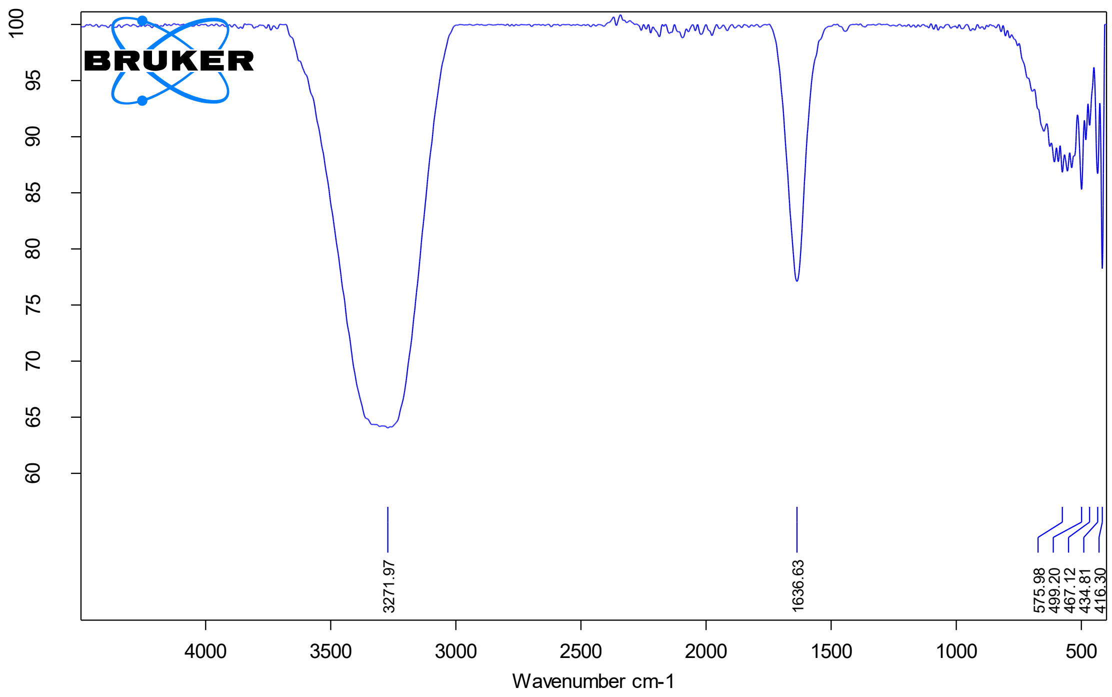

(A)

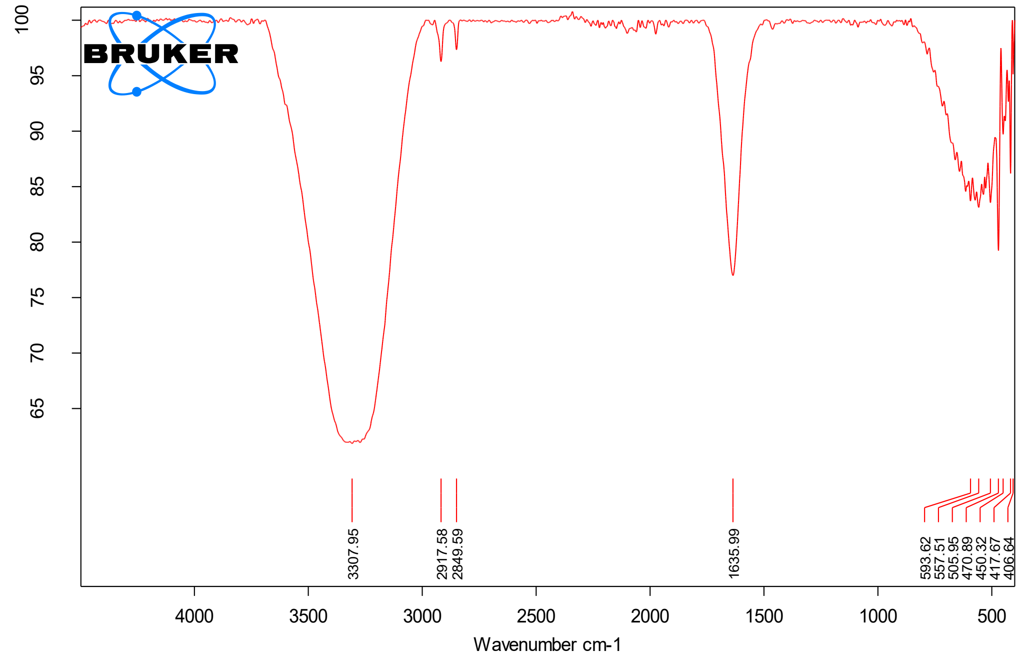

(B)

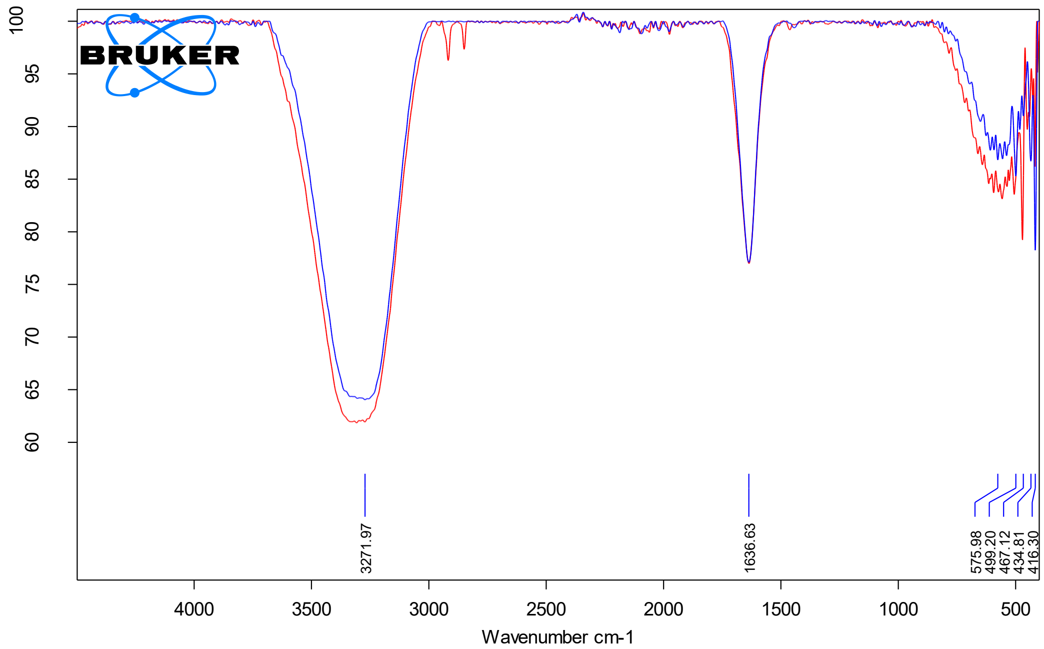

(C)

Figure S4: FTIR chart of pure drug (MOX) (A), F-opt (B) and the overlaid of both charts (A) and (B) in (C)
